# Mathematical analysis of long‐distance polar auxin transport data of pin mutants questions the role of PIN1 as postulated in the chemi‐osmotic theory

**DOI:** 10.1111/ppl.70139

**Published:** 2025-03-13

**Authors:** Kees J. M. Boot, Sander C. Hille, Kees R. Libbenga, Marijke Libbenga‐Nijkamp, Omid Karami, Bert Van Duijn, Remko Offringa

**Affiliations:** ^1^ Plant Biodynamics Laboratory and Department of Plant Developmental Genetics Institute of Biology Leiden, Leiden University Leiden The Netherlands; ^2^ Mathematical Institute Leiden University Leiden The Netherlands; ^3^ Fytagoras Leiden The Netherlands; ^4^ Department of Plant Developmental Genetics Institute of Biology Leiden, Leiden University Leiden The Netherlands; ^5^ Present address: Department of Molecular Biology Institute of Biology and Biochemistry, University of Potsdam Potsdam Germany

## Abstract

The plant hormone auxin (Indole‐3‐Acetic Acid, IAA) is a key player in nearly every aspect of plant growth and development ranging from cell division and cell elongation to embryogenesis and root formation. The IAA level in specific tissues and cells is regulated by synthesis, conjugation, degradation and transport. Especially long‐range polar auxin transport (PAT) has been the subject of numerous studies. The chemi‐osmotic theory predicts that intercellular PAT is caused by an asymmetric distribution of auxin efflux transporters in cell membranes of transporting cells, resulting in increased local membrane permeability for IAA. Members of the *PIN* gene family are generally considered to encode the postulated carriers. The objective of this study was to use the chemi‐osmotic theory in an experimental program aimed at describing and interpreting long‐range PAT data from mutants of the *PIN* gene family *of Arabidopsis thaliana.* Therefore, we put the chemi‐osmotic theory in a broader theoretical framework. We find that the observed decrease in both auxin flux and transport velocity in *pin1* loss‐of‐function mutants is not caused by decreased basal membrane permeability, as would be expected according to the chemi‐osmotic theory, but is an indirect effect caused by a change in the dynamics of auxin transport due to a decrease in the expression of all four AUX1/LAX1‐3 auxin influx carriers in *pin1* mutants. On the basis of our findings, we conclude that the exact role of PIN1 in long‐distance PAT, as postulated in the chemi‐osmotic theory, should be reconsidered.

## 1 INTRODUCTION

Over the past few decades molecular genetics of *Arabidopsis thaliana* (Arabidopsis) have identified a class of proteins, the so‐called pin‐formed (PIN) auxin transport proteins (Adamowski and Friml, 2015; Naramoto, 2017). PIN's molecular structure and asymmetric intracellular localization meet their role as auxin export carriers as predicted by the chemi‐osmotic theory for polar auxin transport (PAT; Rubery and Sheldrake, 1974; Raven, 1975; Su et al., 2022; Ung et al., 2022; Yang et al., 2022). This also correlates well with the direction of observed auxin fluxes (Gälweiler et al., 1998: Benková et al., 2003; Wisniewska et al., 2006). However, the problem with these interesting studies is that they are merely based on correlations and not on auxin transport measurements.

Furthermore, it has still not been resolved in mechanistic terms how auxin transporters, in particular members of the *PIN* gene family, might facilitate PAT (Friml and Palme, 2002; Baluska et al., 2003; Kramer, 2004; Luschnig and Vert, 2014). Recently, cryo‐electron microcopy structures of PIN1, PIN3 and PIN8 were published and shed some light on the transport mechanisms of PINs (Su et al., 2022; Ung et al., 2022; Yang et al., 2022). Concerning auxin transporters of the AUX1/LAX1‐LAX3 family, there is consensus that it consists of auxin‐anion transporters (Yang et al. 2006) that operate as symporters with one or more protons (Hertel 1983, Lomax et al. 1985, Kerr and Bennett 2007, Dindas et al. 2018). Rubery and Sheldrake (1974) assumed an effectively non‐electrogenic transport in their original formulation of the chemi‐osmotic theory. Hertel (1983) suggested a double‐proton symport, which was subsequently experimentally investigated (Lomax et al. 1985).

The complicating factor is that measurements of PAT in organisms, organs, or tissues take place at the macroscopic level, which is, at present, the only level accessible for reliable PAT measurements. We should realize that we are also dealing with underlying processes at the cellular (mesoscopic) and molecular (microscopic) level. In such a situation, with multiple spatial scales, various interacting processes and complicated spatial structures, like those encountered in PAT, logical reasoning alone cannot fully unravel the consequences of changes in the system. In particular, changes in dynamics are difficult to assess in that matter. However, mathematical models of dynamics and their simulations can be very useful. In this article, we will show how the different scales, in our case in long‐distance PAT, can be bridged and the spatial complexity adequately reduced and how we used this in interpreting PAT data from various *pin* mutants of Arabidopsis.

Multiscale mathematical models for auxin transport in various plant organs have been developed by different researchers (e.g. for the root tip: Swarup et al. 2005, Grieneisen et al. 2007, Band and King 2012; see the latter for more examples and an extensive list of references). For long‐distance PAT in inflorescence stems, the seminal work by Mitchison (1980) connects the mesoscopic to the macroscopic scale for PAT cell files, while Chavarría‐Krauser and Ptashnyk (2010) extended this to upscaling from a two‐dimensional multi‐cellular structure consisting of identical PAT cells, using asymptotic analysis.

A quantitative model‐based analysis of measured auxin fluxes during long‐distance PAT at the macroscopic scale of an Arabidopsis inflorescence stem segment under well‐controlled experimental conditions was performed by Boot et al. (2016). They showed that during long‐distance PAT in inflorescence stems, auxin is located within the vascular bundles, both in PAT cell files and surrounding tissues. It is not yet clear in all detail where these files are located within the vascular bundle, both in phloem and xylem. Contrary to the root tip, in the case of PAT in inflorescence stems this uncertainty hampers a bottom‐up upscaling approach by asymptotic analysis that starts from a detailed cellular model.

Hence, instead of investigating and comparing a multitude of possible cellular models in their effects at macroscopic scale through asymptotic analysis, in the currently discussed work we took a top‐down approach starting from the experimentally validated macroscopic model. In this model, auxin content throughout the stem is lumped into a limited number of compartments, each having particular spatial extension, specific auxin transport characteristics in longitudinal direction within them and having exchange of auxin between them in transversal direction. Model parameters obtained from fitting the model to observed PAT data sets characterize the overall auxin transport behavior. Data sets from different well‐defined experimental settings showed relevant changes in particular (‘key’) parameters. The strategy was then to interpret these changes by means of upscaling only specific submodels at a smaller scale that are relevant to these parameters.

To realize this, we started by studying the major theories/models of PAT and finally selected the following three:

1. The chemi‐osmotic theory, as originally put forward by Rubery and Sheldrake (1974) and Raven (1975). This theory describes and explains the unidirectional transport of auxin across interfaces of cells in PAT channels, using microscopic parameters such as the postulated auxin export carriers and where the driving force is the proton‐motive force. However, although this theory is the best known, it falls short in explaining the dynamics of PAT at the macroscopic scale.
2. The models published by Mitchison (1980) and Kramer (2002), which we will call the MK‐model, describe PAT in multicellular PAT channels effectively as an advection–diffusion process. That is, the transport of a solute is driven by the random collision of solvent molecules (diffusion) combined with a directed movement caused by a lasting force on the solute molecule (advection). They derived equations for the advection velocity and diffusion constant as function of the effective permeability constants (denoted as p and q), however, without linking them to the chemi‐osmotic theory.
3. The model published by Boot et al. (2016), in which the authors took the suggestion from the MK‐model to describe the dynamics of long‐range PAT basically by an advection–diffusion process in a continuum approach at the macroscopic level. The parameters of this model were estimated empirically by optimal data fitting. In this article we will call this model the BHL‐model (Boot/Hille/Libbenga) for simplicity.

During the course of the present study, we integrated these theories/models stepwise into abroader Long‐range PAT Theoretical Framework (LPTF). The first step is outlined in Section  3.1, where we used the BHL‐model to interpret long‐range PAT data from a broad range of *pin* mutants of Arabidopsis, using the standard PAT assay as described in Boot et al. (2016). We summarize the anatomical and mathematical structure of the BHL‐model in Box 1, because it formed the background of the advection velocities as key attributes of PAT presented in this section. We found that only PAT data from *pin1* mutants showed lower advection velocities as compared to wild‐type plants and also a substantial reduction of the flux density of PAT. Therefore, these *pin1* mutants were singled out for a closer investigation. We reasoned that we probably could only understand these aberrant transport profiles of the *pin1* mutants, if we should approach them from a broader theoretical framework. Hence, the next step was to integrate the three selected theories of PAT to get the LPTF (Box 2) as described in Section 3.2. The LPTF turned out to be very useful for interpreting PAT data. If supplied with a particular set of microscopic parameters, the LPTF generates a set of macroscopic parameters, among others, the advection velocity, which gives, using the BHL model, good simulations of the PAT data. In Section 3.3 we describe a reassessment of PAT data from *pin1* mutants using the LPTF. This provided an unexpected explanation for the aberrant PAT characteristics of the *pin1* mutants. Instead of a drastic decrease of the microscopic parameter *V* representing the auxin‐anion carrier in the assumed shape of PIN1 proteins, we see a drastic decrease of just the microscopic parameter representing auxin‐anion/H^+^ symporters coded by the *AUX1/LAX1‐3* genes (Yang et al., 2006; Carrier et al., 2008; Péret et al., 2012; Singh et al., 2018), which are generally believed to prevent auxin from leaking out of the PAT channels. This remarkable finding was supported by PAT data from an *aux1/lax1‐3* quadruple mutant, in which all four auxin import carrier genes were knocked out. This quadruple mutant showed PAT characteristics which were quantitatively similar to those of the *pin1* mutant.

**BOX 1:**
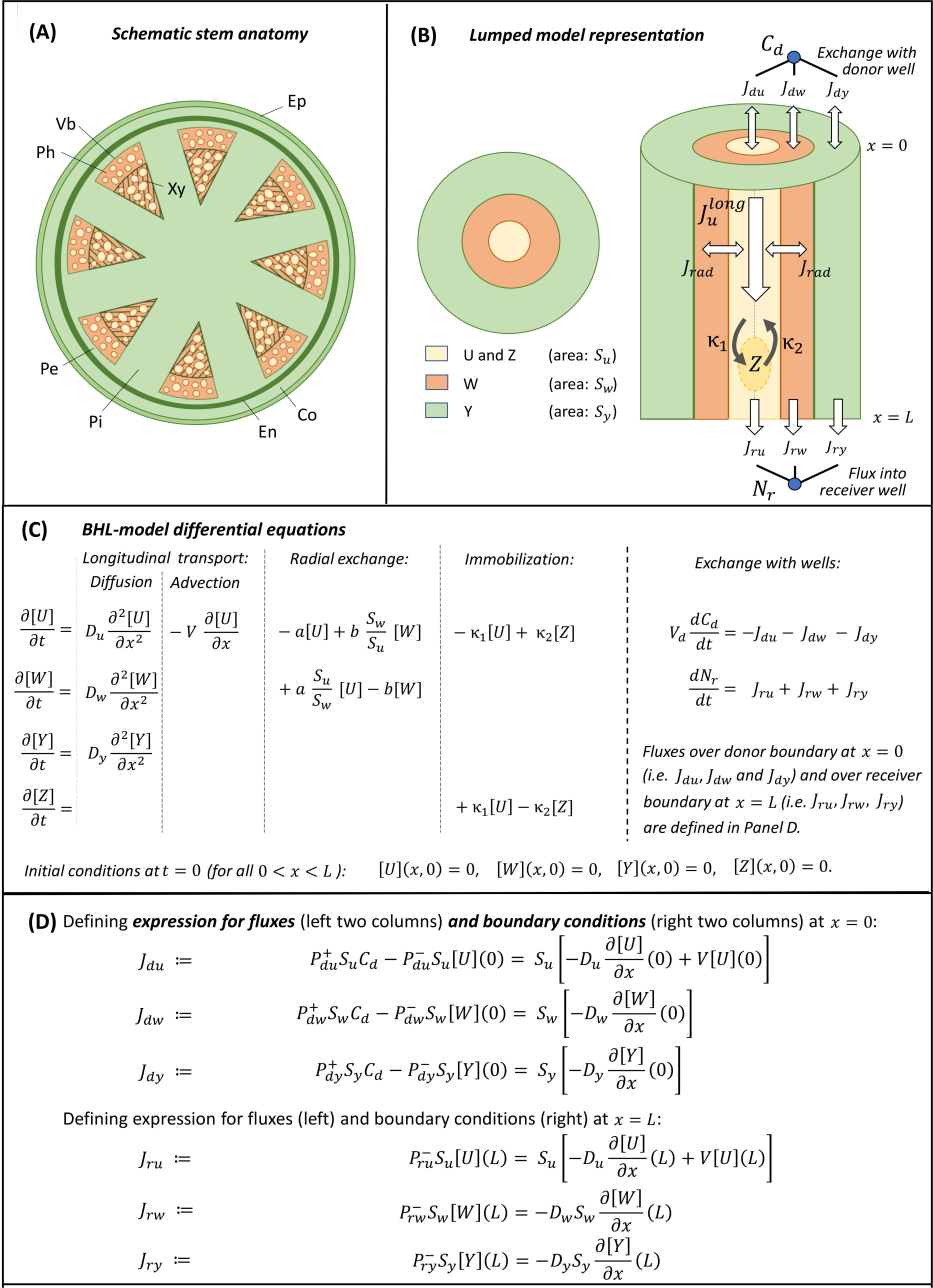
**Summary of the BHL model** (Boot et al., 2016) for long range polar auxin transport as a classical mathematical initial value ‐ boundary value problem in terms of a system of coupled partial differential equations (PDEs). **(Panel A) Schematic presentation of the anatomical structure** of the stem segment (Co, Cortex; Ep, Epidermis; En, Endodermis; Pe, Pericycle; Ph, Phloem; Pi, Pith; Vb, Vascular bundle; Xy, Xylem). See supplemental files for microscopy images of cross sections of a wild type and *pin1* mutant plant. **(Panel B) Lumped anatomy as employed in the BHL‐model**. Four compartments are identified (U, W, Y and Z), where U is the totality of PAT cell files in which PAT occurs within the vascular bundles. W is the complement of U in the vascular bundles. Y aggregates all parts outside the vascular bundles. These have cross sectional areas $S_{u},S_{w}$ and $S_{y}$, which are assumed to be the same at each location of a cross section, which is described by a coordinate x, with x=0 corresponding to the part of the stem segment that is in contact with the medium in the donor well and x=L with that in the receiver well. Z is contained within U and represents immobilized auxin. White arrows indicate fluxes of auxin: between donor and receiver well and the stem segment ($J_{\mathrm{du}},$ $J_{\mathrm{dw}},J_{\mathrm{dy}}$ and $J_{\mathrm{ru}},$ $J_{\mathrm{rw}},J_{\mathrm{ry}},$ respectively) and within the stem segment. Positive values refer to flux in the direction of increasing position x. The exchange flux with the receiver well is taken as unidirectional (positive) from stem segment into the well, because experimentally, this well is frequently sampled and refilled with neutral medium. Reflux from the well into the stem segment can hence be ignored. Within the stem segment, we distinguish transport in longitudinal direction in U (indicated by$\;J_{u}^{\text{long}}$), in W (by diffusion, not shown) and in Y (also by diffusion, not indicated) and transport in radial direction, between the U and W compartments ($\;J_{\mathrm{rad}}$). The longitudinal flux contains the key parameter of the advective velocity V, which is the auxin transport velocity. The radial exchange flux is represented in first‐order approximation by key parameters a and b that represent the exchange rates from compartment U to W and from W to U (see main text, and Panel C). Immobilization and remobilization of auxin within the U compartment is described by rates $\kappa_{1}$ and $\kappa_{2}$, respectively. **(Panel C) The BHL model** is mathematically structured into four PDEs for the variables $\left[U\right]$, $\left[W\right]$, $\left[Y\right]$ and $\left[Z\right]$. These represent the average total concentration of (labelled) auxin (i.e. that of auxin in anion and protonated form combined) in the respective compartments in a cross section at location x and time t: $\left[U\right]=\left[U\right]\left(x,t\right),$ etc. $D_{u},$ $D_{w}$ and $D_{y}$ denote the effective diffusion constants of auxin in the compartments with diffusive transport. There are two additional ordinary differential equations for the concentration of auxin in the donor well ($C_{d}$) and the total amount of IAA accumulated in the receiver well ($N_{r}$). At the start of the experiment, t=0, no labelled auxin is present in the stem segment yet. So, initial conditions for all four variables were set to zero. PDEs must be complemented by boundary conditions (BCs, **panel D**) at the two boundary points x=0 and x=L of the spatial domain 0<x<L, since different BCs typically yield different solutions. The BCs are formulated in such a way that the total flux over the boundary (the $J_{\mathrm{du}},J_{\mathrm{dw}},$ etc., defined by the expressions in the middle column) equals the mathematical expression for the (advective‐diffusive) flux in the spatial domain as given by the PDE at the boundary point (right‐hand column). The boundary fluxes $J_{\mathrm{du}},J_{\mathrm{dw}},$ etc., are expressed in first‐order approximation in their dependence on auxin concentrations. Thus, they are characterized by permeability constants $P_{\mathrm{du}}^{+}$ (etc.) from well into stem segment and $P_{\mathrm{du}}^{-}$ (etc.) from segment into well. $V_{d}$ is the volume of the donor well, which is 1 x 10^−6^ m^3^ in all experimental set‐ups (measured).

**BOX 2:**
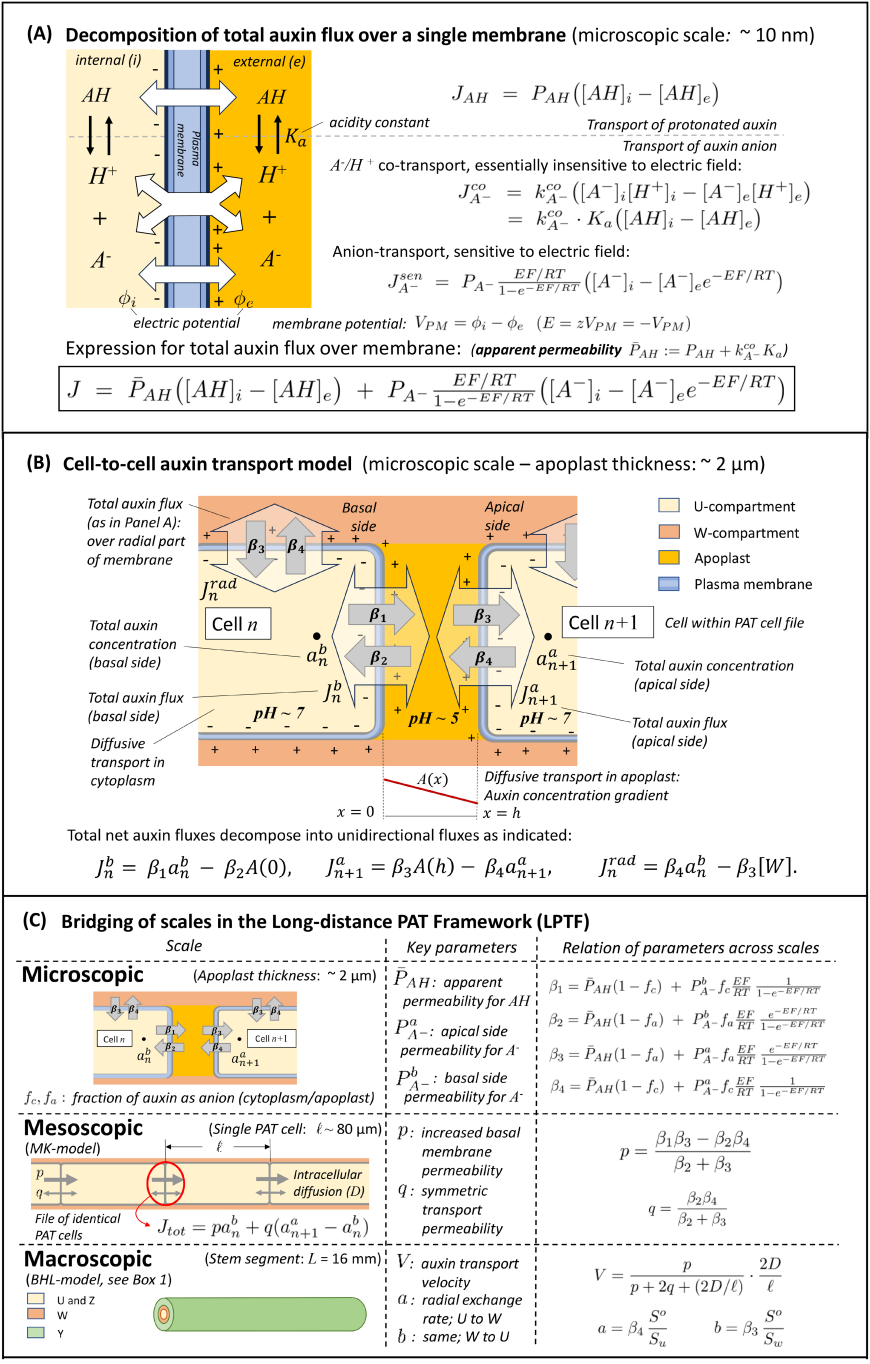
**Structure of the multi‐scale Long‐distance PAT Theoretical Framework (LPTF)**. Models of auxin transport at various spatial scales are combined in the LPTF: that of plasma membrane (PM) transport, cell‐to‐cell transport through the apoplast, PAT through the multi‐cellular cell files of the Mitchison‐Kramer (MK) model, and finally PAT through the inflorescence stem segment in the donor/receiver well experimental configuration as represented by the BHL‐model, summarized in Box 1. Central in LPTF is the principle that auxin fluxes over membranes must be decomposed in the transport of the auxin anion and that of the protonated form **(Panel A)**, because of the presence of an electric field over the PM that influences transport characteristics of the anion, and the existence of different types of auxin anion carriers. For example, the AUX1/LAX1‐LAX3 carriers, evenly distributed over the membrane, are supposed to be anion/*H* ^+^ co‐transporters, while the PIN1 proteins, concentrated at the basal PM, are possibly anion/*Na* ^+^ antiporters (Yang et al., 2022). The total flux density J (mol/m^2^.s) of IAA (anion and protonated form together) is decomposed into the flux density $J_{\mathrm{AH}}$ of the protonated form and that of the anion form. The latter is split into a part $J_{A^{-}}^{\mathrm{sen}}$ that is sensitive to the electric field related to the membrane potential, $V_{\mathrm{PM}}$ (~ 120 mV; Raven 1967), and a part that is effectively insensitive due to electrically neutral symport, $J_{A^{-}}^{\mathrm{co}}$. Each of these flux densities has a different expression as function of the concentration of the various auxin species on either side of the PM (**Panel A**). The expression for $J_{A^{-}}^{\mathrm{sen}}$ follows Raven (1975). That for $J_{A^{-}}^{\mathrm{co}}$ is a first order approximation of a generic expression for symporters, derived in Sanders et al. (1984), in which, in its second formulation, the assumption of fast balancing between anion and protonated form according to the acidity constant $K_{a}$ and local pH is used (see supplemental files for details). A key observation is, that the second expression for $J_{A^{-}}^{\mathrm{co}}$ is apparently that of transport of the protonated form. Thus, the total auxin flux density over the PM is characterized by an apparent permeability ${\bar{P}}_{\mathrm{AH}}$ (m/s) for the protonated form as indicated, and a permeability $P_{A^{-}}$ for the auxin anion. According to the chemo‐osmotic theory for PAT, IAA is transported from the n‐th cell in the PAT cell file to the next cell (numbered n+1) through the intermediate apoplast (**Panel B**). The totality of PAT cell files in the vascular bundles form the U‐compartment in the BHL‐model (recall Box 1). Total net transport fluxes $J_{n}^{b}$ and $J_{n+1}^{a}$ of IAA through the basal and apical PMs are each modelled as in **Panel A**. Their expression can be split, conceptually, as indicated in **Panel B**, as a difference of two unidirectional fluxes that are proportional to the total auxin concentration close to the PM at the side of departure of the flow, with proportionality constants $\beta_{1}$ to $\beta_{4}$. A similar decomposition is used for the total radial flux of auxin from the cytoplasm of the PAT cell file to the neighboring tissue, the W‐compartment in the BHL‐model. **Panel C** provides the explicit expressions for the $\beta_{i}$, which include the physical universal gas constant R, Farday's constant F, absolute temperature T and the fractions $f_{c}$ and $f_{a}$ of auxin in anion form in cytoplasm and apoplast, respectively. The apical side permeability of the PM for the anion, $P_{A^{-}}^{a}$ (1.5x10^−8^ m/s), is taken as a fixed basal level, applying to the radial part of the PM too. The basal side permeability $P_{A^{-}}^{b}$ is assumed larger than $P_{A^{-}}^{a}$, due to the presence of PIN1 proteins. The apparent permeability for the protonated form, ${\bar{P}}_{\mathrm{AH}}$, is assumed the same for all parts of the plasma membrane, corresponding to the homogenous distribution of AUX1/LAX1‐LAX3 carriers. The threefold PM‐apoplast‐PM barrier can be reduced to an effective single barrier as in the MK‐model, which has key parameter p for the effective increased basal permeability in terms of total IAA concentration and a diffusion‐like non‐polar effective transport characterized by a parameter q. They can be expressed in $\beta_{1}$ to $\beta_{4}$ of the microscopic model as indicated (see SM for details). The key parameters in the BHL‐model, auxin transport velocity V and the total auxin exchange rates a and b from U‐ to W‐compartment and vice versa, can then be expressed in the $\beta_{1}$ to $\beta_{4}$ as well, using the auxiliary mesoscopic parameters p and q, as indicated (again, see SM for details). The LPTF consists of the thus obtained totality of relationships and dynamic models at different scales.

In addition, in Section 3.4 we describe that the expression levels of the *AUX1/LAX1‐3* genes are substantially lower in *pin1* mutants than in wild‐type plants.

The results section gives a more elaborate substantiation of the successive steps as described in the above introduction. In this, we could not avoid some applied mathematics, but the equations are explained, while in most cases their derivation is given in the supplementary material (SM). In the discussion, we will comment upon the remarkable outcome of the present study. It shows that a deeper understanding of the dynamics of PAT in the shape of the multiscale LPT, on the one hand provided a ready explanation for the characteristically aberrant PAT profiles of *pin1* mutants, while on the other hand it raised a serious question as to the exact role of in particular PIN1 proteins in long‐range PAT.

## 2 MATERIALS AND METHODS

### 2.1 Plant material and growth conditions

For all experiments, *Arabidopsis thaliana* ecotype Col‐0 or mutant lines in that same background were used. Plants were grown on a mixture of 9:1 substrate soil and sand (Holland Potgrond) at 21°C, a 16‐hour photoperiod, and 70% relative humidity. Seeds from *pPIN1::PIN1:GFP* in *pin1* mutant background and two different *pML1::PIN1:GFP* in the *pin1* background (line A and B) or one line in the *pin1, pin4* background and the *pin1* mutant were provided by C. Kuhlemeier (Kierzowski et al., 2013). The *aux1, lax1,2*,3 quadruple mutant was described previously (Boot et al., 2016). *pin3,4,7* triple‐mutant seeds were obtained from O. Leyser (Bennett et al., 2016; Waldie and Leyser, 2018) and *pgp1,19* double‐mutant seeds were obtained from A. Murphey (Blakeslee et al., 2007). *pin5,6,8* seeds were provided by E. Scarpella (Sawchuk et al., 2012).

### 2.2 Polar auxin transport measurements

The first set of PAT measurements were performed as described earlier (Boot et al., 2016). In short: the donor and receiver wells were filled with MA medium supplemented with 10 mM MES (pH = 4.8). Routinely, the donor wells contained 10^−4^ mol/m^3^ (10^−7^ M) ^3^H‐labelled IAA 3‐[5(n)‐^3^H] IAA, specific activity 25 Ci mmol^−1^ (Scopus Research BV). At regular time intervals receiver wells were emptied and replenished by fresh medium. The incubation time was usually 300 min. For the extended PAT measurements, the donor wells containing ^3^H‐IAA were replenished after 300 min with MA buffer without ^3^H‐IAA. Samples were drawn from the receiver wells for an additional 300 min. Radioactivity of the samples was measured in an LKB liquid‐scintillation counter. For determining tissue profiles, segments were cut into 4‐mm‐long pieces and transferred to scintillation liquid.

For the second set of PAT measurements a parallel batch of stem segments was run alongside the experimental set. The incubation time was standard 300 min. One part of the two sets was used for determining the tissue profile, the other part was used for determining the amount of immobilized IAA.

### 2.3 TLC analysis of immobilized IAA

The middle 4–12 mm parts of the parallel batch of stem segments were ground in liquid N_2_ and extracted twice with 100% ethanol. After centrifugation (10 minutes at 20.000 g) the supernatants were dried in a speedvac and resolved in a small volume of ethanol. Samples were spotted on silica gel60 F254 fluorescent aluminum TLC plates (Merck) and separated in a solvent containing n‐hexane:ethylacetate:isopropanol:acetic acid (40:20:5:1, v/v). After running, the TLC plate was divided into nine 1.5‐cm sections, which were cut off and added to liquid‐scintillation vials and counted in an LKB liquid‐scintillation counter.

### 2.4 Anatomy of the inflorescence stem

To determine the parameters S (the cross‐sectional area of a stem segment) and $S_{\mathrm{vb}}$ (sum of cross‐sectional areas of vascular bundles), transverse sections from the basal part of inflorescences of Arabidopsis were made with a table‐bench microtome at a thickness of 150 μm and mounted on a glass slide in water. The sections were then photographed using a Zeiss Axioplan Imaging upright light microscope (Carl Zeiss), equipped with a Zeiss Axiocam MRC 5 digital camera. By cutting and weighing images of the sections the percentage of $S_{\mathrm{vb}}$ per stem segment was determined.

### 2.5 RNA isolation and RT‐qPCR analysis

RNA was isolated from basal inflorescence stem segments using the RNEasy© kit (Qiagen). First‐strand cDNA was synthesized using the RevertAid RT Reverse Transcription kit (Thermo Fischer Scientific). Quantitative PCR was performed on three biological replicates along with three technical replicates using the SYBR‐green dye premixed master‐mix (Thermo Fischer Scientific) in a C1000 Touch© thermal cycler (BIO‐RAD). CT values were obtained using Bio‐Rad CFX manager 3.1. The relative expression level of genes was calculated according to the 2^−ΔΔCt^ method (Livak and Schmittgen, 2001).

Expression was normalized using the *β‐TUBULIN‐6* gene. Three biological replicates were performed, with three technical replicates each. The primers used are described in Table 5 of the SM.

### 2.6 Mathematical model

For the interpretation of the data, we used the mathematical BHL‐model that we developed in our previous paper (Boot et al., 2016). We identified four modes of behaviour for the labeled IAA and distinguished equally many tissue regions within the inflorescence stem segment for the location of IAA (called ‘compartments’): the U‐compartment contains the polarly transported IAA; the W‐compartment contains the free IAA that is transported simply by diffusion; the Z‐compartment immobilized IAA (no transport) and the Y‐compartment contains any IAA that is isolated from the transport system and located outside of the vascular bundles (see Box 1 for a summary and supplemental material for details). The model describes how the average concentrations [*U*], [*W*], [*Y*] and [*Z*] of labelled IAA in each of the corresponding compartments change in time t along the stem segment of length L at position x from the end of the segment that is in the donor well (0≤x≤L).

### 2.7 Numerical simulation and data fitting

Numerical simulation of the partial differential equations was performed in the COMSOL Multiphysics 4.2a finite‐element package (version 4.2.1.110) using the generalized‐alpha time‐dependent solver. For adequate resolution of the dynamics near the boundaries, the finite‐element mesh was manually refined at both boundaries. As a linear solver we used Direct1 (PARDISO). Computations ran in COMSOL Server 4.2a, coupled to MATLAB R2008b (version 7.7.0.471) through the ‘LiveLink for MATLAB’ interface. Parameter optimization algorithms were implemented in MATLAB, using COMSOL as solver for the system of partial differential equations.

The total quality of fit for a simulation in comparison to all different types of observations made (transport profile, tissue profile and IAA immobilization) has been expressed in terms of relative quantities for the quality of fit for each of these three types separately, because the corresponding observables have different ‘nature’, hence physical dimension, and order of magnitude. Fair comparison can then be made only through relative quantities.

The comparison of the transport profile in PAT or extended PAT experiment to simulation was quantified as the square root of the sum over all measurement time points of the squared difference between observed and simulated amount, divided by the total amount of auxin transported at the end of the experiment. Similarly, the quantifier of fit for the tissue profile is the square root of the sum of squared differences between simulation and observation of the total amount of auxin in the 4‐mm‐long sub‐segments used in the determination of tissue profiles at the end of an experiment, divided by the total amount of auxin present in the tissue. The first 4‐mm segment was ignored, because the amount of auxin that ends up outside the transport system (in the so‐called ‘Y‐compartment’) is considered an artifact of the way the donor side of the stem segment is exposed to auxin, rather than a characteristic of PAT, in which we are interested. Finally, the amount of immobilized IAA in the middle 4‐12‐mm part of the stem segments as obtained through the additional TLC analysis in the second set of PAT measurements is considered relative to the total amount of IAA in this middle part, resulting in the immobilization ratio. The total quality of fit is then the sum of the indicated relative quantities for transport and tissue profile in the first set of PAT measurements and the sum of all three in the second set of measurements. The resulting function we call the *cost function*.

After manually initializing the fitting, automated optimization of the cost function was done on the COMSOL‐MATLAB‐coupled software platform. We used the Gradient Descent Method, where the step size is determined by linear search in negative gradient direction to increase rate of convergence. The Golden Ratio method was used to find an approximation of a minimum of the cost function during linear search. Optimization was stopped when the cost function dropped below the threshold of acceptability of 6%. The resulting fit we call optimal.

### 2.8 Statistical analysis

Statistical computations on the first set of PAT measurements were made in RStudio, version 1.4.1106. For all tests a standard confidence level of 5% was taken. Clustering of the various wild‐type and mutant batches of plants was done on the hypothesis of having the same mean value for the log‐transformed values of the fitted transport velocity V. It was made by inspection of a boxplot of the log‐transformed values for each individually examined stem segment per batch, assisted by the results of pair‐wise Tukey post‐hoc analysis on the batches. For each visually defined cluster the hypothesis of having the same mean logV‐value was tested by means of one‐way ANOVA. Then, for each visually defined cluster any single batch outside this cluster was added and the one‐way ANOVA test was applied to the thus extended cluster. If all the extended clusters could not confidently be considered to be a cluster of batches with the same mean log‐velocity value as the original cluster, then the latter was considered definitively to be a cluster.

## 3 RESULTS

### 3.1 Interpretation of PAT data from a broad range of *pin* mutants within the context of the BHL‐model

The family of PIN proteins in Arabidopsis consists of eight members, which can be divided into two groups: PIN1, PIN2, PIN3, PIN4 and PIN7 having a long central cytosolic loop, which are located at the plasma membrane (PM), and PIN5, PIN6 and PIN8 with a short(er) central cytosolic loop, which are located at the endoplasmic reticulum (ER), although PIN6 is also present at the PM (Křeček et al., 2009; Ditengou et al., 2018; Abdollahi Sisi and Růžička, 2020). Since *PIN2* expression is regarded as mainly root‐specific (Li et al., 2015), we excluded it from our analysis and restricted our experiments to the other seven shoot‐expressed *PIN* genes. Even though PIN5,6 and 8 are located at the ER and are assumed to play a role in intracellular auxin regulation, they were included in our analysis as well.

In addition to the wild‐type controls, we analyzed PAT in inflorescence stems of the *pin1* and *pin3* single mutants, and of the *pin3,4,7* (Bennett et al., 2016; Waldie and Leyser, 2018) and pin*5,6,8* (Sawchuk et al., 2013) triple mutants. The *pin1* mutant has an aberrant needle‐like inflorescence (Okada et al., 1991) and in this respect is different from the other mutants, which all have a more or less wild‐type inflorescence phenotype. We also included inflorescence stem segments (ISS) from the following complemented *pin* mutants with a wild‐type inflorescence phenotype: 1) *pPIN::PIN1:GFP*/*pin1*, where a functional PIN1:GFP fusion is expressed under the PIN1 promoter in the *pin1* mutant background, 2) *pML1::PIN1:GFP/pin1*, where the PIN1‐GFP protein is expressed in the *pin1* mutant background under the L1 layer‐specific pML1 promoter, and 3) *pML1::PIN1:GFP* in the *pin1, pin4* double mutant background. Especially the latter two lines are interesting, as they do develop normal inflorescences, whereas PIN1 is not expressed in the vascular bundles of the inflorescence stems, where the long‐distance PAT takes place (Kierzkowski et al., 2013). In addition, we included the *pgp1,19* double mutant in our analysis. Both PGP1 and PGP19 belong to the P‐glycoprotein multidrug resistance and ATP‐binding cassette subfamily B of putative auxin transport proteins (Geisler and Murphy, 2006). This *pgp1,19* mutant line has strongly reduced inflorescences.

We made full model‐based simulations of efflux and tissue profiles from either standard or extended PAT donor‐receiver assays. The simulations were performed using the transport profiles of each individual ISS in a batch, which usually contained nine ISS.

**FIGURE 1:**
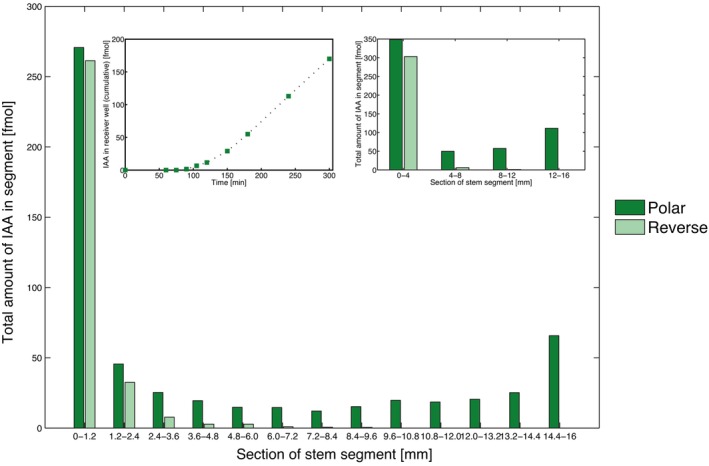
**Example of a tissue profile of a wild‐type stem segment**. The graph shows the IAA tissue profile of a 16 mm long basical part of an Arabidopsis inflorescence stem, after 300 min of exposure to tritium‐labelled IAA, cut into 1.2 mm sections. Placement of segment in polar orientation (dark green) and reverse (light green). The inset shows the efflux profile of this stem segment (left). Routinely, in standard essays, we use a tissue profile consisting of four 4 mm sections. An example of such a profile is shown in the inset (right), which is computed from the detailed profile.

Figure 1 shows an example of an efflux and tissue profile of an individually measured ISS from a wild‐type reference batch of Arabidopsis plants. For PAT measurements, these stem segments were placed in the assay in the polar (apical to basal) orientation. As expected, no PAT could be measured when stem segments were placed in the reverse orientation (Figure 1) or when the PAT inhibitor NPA was added to the donor well (Boot et al., 2016).

Since all simulations were performed within the context of the BHL‐model, we give a summary of that model in Box 1. It shows both a presentation of the anatomical context (Panel A and B) and the mathematical structure (Panel C and D) of the BHL‐model. A full account of the development of the BHL‐model and its experimental validation is given in Boot et al. (2016). See also supplemental material, Section 1. With respect to PAT, the most fundamental part of the BHL‐model is the longitudinal flux of auxin through the *U*‐compartment, which represents the tissue region of the stem segment that contains IAA that is polarly transported (see Box 1, Panel B). This polar transport flux in longitudinal direction may be written as:

(1) $$J_{u}^{\text{long}}\left(x_{,}t\right)=-D_{u}\frac{\partial \left[U\right]}{\partial x}\left(x_{,}t\right)\;S_{u}+V\left[U\right]\left(x_{,}t\right)\;S_{u},\;0<x<L,$$

where $D_{u}$ is the diffusion coefficient of auxin in the U‐compartment, $\;S_{u}$ is the cross‐sectional area of the U‐compartment and V is the advection velocity. This equation is related to the first terms in the partial differential equation (PDE) in Box 1, panel C, describing the change over time of the concentration [U] of auxin in the U‐compartment, and which represents the suggestion made by Mitchison (1980) and Kramer (2002) that PAT may be best described by an advection–diffusion equation in a continuum approach at the macroscopic level. Another important flux equation describes the radial exchange of auxin between the U‐ and the W‐compartment of the BHL‐model (see Box 1, panel B and C). However, its importance can be best explained in the next section.

**FIGURE 2:**
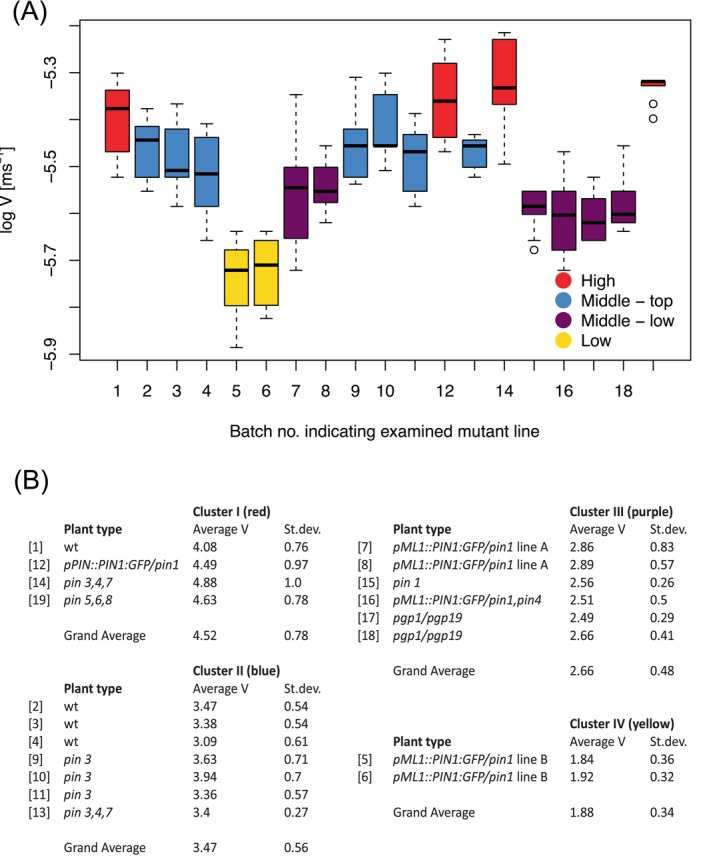
**Clustering of mutants according to auxin transport velocity. A** Boxplot of the four groups subdivided according to the 10‐base logarithm of their velocity V (expressed in ms^−1^) after simulation of their PAT data with the BHL model. Small circles represent weak outliers. Typically, 8–9 individual ISS have been measured once per batch (supplementary files, Table 4 for further details). (B) Summary statistic of the four different clusters with different advection velocities V, expressed in 1x10^−6^ ms^−1^, that could be identified based on statistical analysis as described in the Main Text. Batch numbers are indicated in brackets.

The key attribute to PAT in Equation (1) is the advection velocity. We reasoned that if the family of PIN proteins represent the specific auxin‐anion carriers, as postulated in the chemi‐osmotic theory, then we should expect that in the selected knock‐out *pin* mutants the longitudinal transport of auxin would relapse into simple diffusion so that in principle in such mutants no PAT will be detected in the experimental bioassay. Therefore, in the interpretation of the experimental PAT data, we focused on the advection velocities. In total 19 batches, each containing typically 8–9 ISS individually measured once, from either wild‐type or mutant plants were analysed using the BHL‐model (Figure 2, further details in supplemental material, Table 4).

We took the advection velocities for statistical analysis from the parameter list of each of such batches. In the fitting procedure for the BHL‐model we have experienced that the quality of fit is sensitive to the value for V. Thus, auxin transport velocity is well‐determined by the PAT data, with an estimated relative error of about 5% (Section 3.3 for further discussion). Our objective was to test the null hypothesis that plants in all batches have the same auxin transport velocity, or – phrased more formally – that the mean advection velocities V in all 19 experimental batches are the same.

To this end we performed a one‐way ANOVA. This test assumes, in addition to the assumption that all observations are independent, which was readily satisfied, the following: the values are normally distributed within each group (in our case within each batch) and the variances in each batch are equal, and there are no outliers in any batch. We found, using a Shapiro–Wilk test for normality on each of the batches and a Levene's Median Test (following Hines and O'Hara Hines (2000)) for equal variances among the batches, that both conditions could be satisfied if we used log‐transformed V values. This result was supported by a one‐way ANOVA on the log‐transformed V parameter values to evaluate if the mean is the same among all batches, consisting of ISS from wild‐type and mutant plants. It turned out that mean advection velocities were significantly different between the batches (F(18,138) = 19.18, *p* = 4.12 x 10^−29^, generalized eta squared = 0.71). A visual inspection of the boxplot in Figure 2A confirms this.

The next step was to examine whether the batches may be clustered such that the log‐transformed V values associated with each batch have the same mean. To this end, we used a Tukey post‐hoc analysis. Inspection of its output indicated four potential clusters of batches on each of which we performed a one‐way ANOVA to test the hypothesis that within the potential clusters the batches may be considered to have the same mean (log‐)advection velocity. There was no reason to reject this hypothesis. In all the statistical analysis tests, we used a confidence level of 5%.

In Figure 2B we show that we could distinguish four clusters: Clusters I and II comprise plants that have a wild‐type phenotype and a wild‐type advection velocity, with an average of 4.5 x 10^−6^ ms^−1^ and 3.5 x 10^−6^ ms^−1^ (1.6 and 1.3 cm.hr.^−1^), respectively. Besides true wild‐type plants used in this study, Cluster I also includes the *pPIN::PIN1:GFP/pin1*, which can be considered to be a control wild‐type line for all the other single and multiple *pin1* mutants containing the *pML1::PIN1:GFP* construct. Mutants that left the *PIN1* gene intact, such as *pin3*, *pin3,4,7* and *pin5,6,8* also belong to the wild‐type Clusters I and II. Interestingly, Clusters III and IV with significantly lower advection velocities, with an average of 2.7 x 10^−6^ ms^−1^ and 1.9 x 10^−6^ ms^−1^ (1.0 to 0.7 cm.hr.^−1^) respectively, were exclusively occupied by all the single and multiple *pin1* mutants consisting of *pML1::PIN1:GFP/pin1, pML1::PIN1:GFP/pin1, pin4* mutant lines and the *pin1* single mutant and the *pgp1/pgp19* double mutant lines. Although the advection velocities in Clusters III and IV are somewhat lower than those of Clusters I and II, they are still well within the range of transport velocities of 1.4 x 10^−6^ ms^−1^ to 5.5 x 10^−6^ ms^−1^ (0.5 to 2.0 cm.hr.^−1^) that were found by Kramer et al. (2011) after compiling data from 119 (non‐model based) estimates of PAT velocities from 35 different plant species resulting in a median velocity value of 1.75 x 10^−6^ ms^−1^ (0.63 cm.hr.^−1^).

The conclusion we were compelled to draw from the above results is that none of the *pin1* mutants have advection velocities near zero. Therefore, in view of the chemi‐osmotic theory, there should still be a basal increased membrane permeability for the auxin anion in order to have PAT. However, the average auxin transport velocity is significantly lower in the Clusters III and IV, which exclusively contain plants with mutant *pin1* genes. A model is needed to quantify and understand the possible origins of this reduction in velocity. Therefore, we singled out these *pin1* mutants for a further study aimed at a better understanding of the relationship of PIN1 proteins in the chemi‐osmotic theory and the nature of advection velocities as key attribute in the BHL‐model. This led to the development of the multi‐scale Long‐distance PAT Framework (LPTF), which integrates the BHL‐model, MK‐model and the chemi‐osmotic theory and which includes microscopic parameters that can be related to mutations. This framework and a multiscale analysis of experimental PAT data within the LPTF will be presented in the next two sections.

### 3.2 The multi‐scale Long‐distance PAT Framework (LPTF)

The experimental PAT data sets are obtained for 16‐mm long inflorescence stem segments in the controlled set‐up of an IAA donor ‐ receiver well configuration in a Petri dish (see M&M). The BHL‐model has been developed and experimentally validated to identify a minimal number of regions with the stem segment (the four model‐compartments *U, W, Y* and *Z*) and associated auxin transport characteristics for the IAA within, to describe adequately the measured auxin flux through the segment (Boot et al. 2016). Key parameters in this macroscopic model are the auxin transport velocity, visible as the advection speed V in the model, and the parameters a and b that control the rate of exchange of IAA between the PAT files (U‐compartment) and the surrounding tissue in the vascular bundles (W ‐compartment), see Box 1 and supplemental material for details.

Comparison of fitted parameters sets for PAT data of wild type plants with those of *pin1* mutants yields shifts in the key parameter values. In order to interpret these shifts in macro‐level parameters, we need to relate these parameters to physiological processes at the spatial scale at which the PIN1 protein functions. This scale is that of transport over the plasma membrane, roughly 10 nm thick, in the PAT cell files.

In‐between these scales is that of cell‐to‐cell transport of IAA. This `mesoscopic scale’ has been captured in the well‐established MK‐model (Mitchison 1980, Kramer 2002), to which we want to relate. The purpose of the LPTF is to provide a mathematical modelling framework that bridges these scales with a minimal – but adequate – level of complexity of the model description. In this the key parameters at the macroscopic level of the stem segment are related explicitly, mathematically, to the key parameters of auxin transport at the microscopic level of parts of the plasma membrane. Key parameters are the auxin transport velocity V and radial exchange rates a and b that characterize the exchange of auxin from U‐ to W‐compartment and vice versa. Thus, the obtained expressions allow to interpret changes in macroscopic parameters as changes in microscopic parameters, which in turn correspond to physiological changes caused by the mutations.

IAA is a weak acid. Therefore, the acidity on either side of the membrane determines the fraction of IAA that is in anion form. It can be computed through the acidity constant of IAA (Mitchison, 1980):

(2) $$K_{a}=\frac{\left[A^{-}\right]\left[H^{+}\right]}{\left[\mathrm{AH}\right]}=1.58\times 10^{-5}\;M.$$

Central in the framework at microscopic level is the systematic treatment of auxin fluxes over membranes as being decomposed in the transport of the auxin anion $A^{-}$, and that of the protonated form AH (Box 2, Panel A). These have different transport characteristics. The presence of an electric field over the cell membrane, related to the membrane potential $V_{\mathrm{PM}}$ (~ −120 mV; Raven, 1967), influences the transport of the auxin anion. Moreover, there are various types of auxin anion carriers located at different locations in the membrane. For example, the AUX1/LAX1‐LAX3 carriers, evenly distributed over the membrane, are supposed to be $A^{-}$/$H^{+}$ symporters that transport the anion together with one or more protons in the same direction (Hertel 1983, Lomax et al. 1985, Yang et al., 2006; Carrier et al., 2008; Péret et al., 2012; Sing et al., 2018), while the PIN1 proteins, concentrated at the basal cell membrane, are thought to be anion carriers, possibly anion antiporters (Yang et al., 2022).

Close inspection of the experimental evidence brought forward for a two‐proton stoichiometry for the AUX1/LAX1‐LAX3 carriers (Hertel 1983, Lomax et al., 1985; conducted in zucchini membrane vesicles) is insufficiently conclusive to support the two‐proton assumption. We deem the experimental system not sufficiently well‐defined and intrinsically complex in its auxin and proton transport dynamics. Although Lomax et al. (1985) argue for a two‐proton symport in the discussion, they do so with some reservations.

Rubery and Sheldrake (1974) take a non‐electrogenic transport as assumption. In accordance with our objective of putting the chemi‐osmotic theory in a broader theoretical framework and the reservations for the two‐proton symport, we follow Rubery and Sheldrake in the simpler assumption of an auxin‐anion / single‐proton symport model for the AUX1/LAX1‐LAX3 carriers.

Auxin in protonated form is electrically neutral and can diffuse through the membrane, insensitive to the electric field (with flux density $J_{\mathrm{AH}}$). The co‐transport of anions and protons, e.g. by proteins of the AUX1/LAX1‐3 family, is electrically neutral and therefore may be considered essentially insensitive to the electric field (with flux density $J_{A^{-}}^{\mathrm{co}}$). Instead, the transport of IAA anions by PIN1 proteins, is sensitive to electrical fields (with flux density $J_{A^{-}}^{\mathrm{sen}}$).

We use a first‐order description for each of these three fluxes in terms of the concentrations of protons and IAA species on either side of the membrane (Box 2, Panel A). Accordingly, $J_{\mathrm{AH}}$ is characterized by the membrane permeability $P_{\mathrm{AH}}$ (m/s) for IAA in protonated form. The expression for $J_{A^{-}}^{\mathrm{sen}}$ involves factors depending on the membrane potential. It follows Raven (1975) and is essentially the celebrated Goldman‐Hodgkin‐Katz current equation from electrophysiology (Goldman, 1943; Hodgkin & Katz, 1949). The key parameter for this type of transport is the membrane permeability $P_{A^{-}}$ for the IAA anion in absence of the electric field (when $V_{\mathrm{PM}}=0$). The expression for the proton‐co‐transport of the IAA anion, $J_{A^{-}}^{\mathrm{co}}$, is a first order approximation of the expression for symporter transport obtained by Sanders et al. (1984; supplemental material, Section 3).

Under the assumption that equilibration of the IAA anion and its protonated form is fast compared to the membrane transport processes, Equation (2) in the form of

$${\left[A^{-}\right]}_{i,e}=K_{a}\frac{{\left[\mathrm{AH}\right]}_{i,e}}{{\left[H^{+}\right]}_{i,e}}$$

can be used to rewrite the expression for $J_{A^{-}}^{\mathrm{co}}$ to a form that resembles that for $J_{\mathrm{AH}}$. That is,

$$J_{A^{-}}^{\mathrm{co}}\;{=k}_{A^{-}}^{\mathrm{co}}K_{a}\left({\left[\mathrm{AH}\right]}_{i}-{\left[\mathrm{AH}\right]}_{e}\right)$$

(Box 2, Panel A). Thus, model‐wise, the $A^{-}$/$H^{+}$ co‐transport may be viewed as an apparent transport of the protonated form. Hence, the total flux density of IAA over the membrane,

$$J=J_{\mathrm{AH}}+J_{A^{-}}^{\mathrm{co}}+J_{A^{-}}^{\mathrm{sen}},$$

can be written as $J={\bar{J}}_{\mathrm{AH}}+J_{A^{-}}^{\mathrm{sen}}$, where

$${\bar{J}}_{\mathrm{AH}}={\bar{P}}_{\mathrm{AH}}\left({\left[\mathrm{AH}\right]}_{i}-{\left[\mathrm{AH}\right]}_{e}\right),\text{with}\;{\bar{P}}_{\mathrm{AH}}=P_{\mathrm{AH}}+k_{A^{-}}^{\mathrm{co}}K_{a},$$

is the total flux density of non‐electrogenic transport of IAA, characterized by an *apparent permeability* ${\bar{P}}_{\mathrm{AH}}$, visible in the equations as the sum of transport of the ‘true’ protonated form of auxin and the auxin‐anion/single‐proton symport that appears as transport of the protonated form. The permeability $P_{A^{-}}$ determines the flux of the auxin anion form ($J_{A^{-}}^{\mathrm{sen}}$) that is sensitive to the electric field (Box 2, Panel A). We shall assume that the membrane potential remains constant. That is, fluctuations on a time scale faster than the membrane transport processes are ignored.

At cellular level, we identify three particular parts of the plasma membrane of a cell in a PAT cell file, according to their orientation in the inflorescence stem segment: the apical side, the basal side and radial side, transversal to the direction of PAT, which is from apical to basal (Box 2, Panel B). Superscripts a, b and rad are used to distinguish fluxes and permeability constants at these parts. Thus, following the chemi‐osmotic theory, PAT occurs because the acidity of the apoplast (pH ~ 5) is higher than that of the cytoplasm (pH ~ 7) and the basal side has a higher permeability for the IAA anion than the apical side:

$$P_{A^{-}}^{b}>P_{A^{-}}^{a}.$$

We assume that the permeability for the anion at the radial part of the membrane is not different from that at the apical side:

$$P_{A^{-}}^{\mathrm{rad}}=P_{A^{-}}^{a}.$$

Since the AUX1/LAX1‐3 proteins are evenly distributed over the three parts of the membrane and the permeability of the membrane for the protonated form is considered the same everywhere, the apparent permeabilities are assumed to satisfy

$${\bar{P}}_{\mathrm{AH}}^{a}={\bar{P}}_{\mathrm{AH}}^{b}={\bar{P}}_{\mathrm{AH}}^{\mathrm{rad}}.$$

We shall denote this common value by ${\bar{P}}_{\mathrm{AH}}$.

The expressions for the fluxes distinguish the auxin species and their concentrations. Experimentally, we cannot measure the concentrations of the different auxin species. Therefore, the BHL‐model was described in terms of total auxin concentrations in the various compartments. Thus, in order to bridge the scales to the level of the BHL‐model, it is convenient to express the fluxes in terms of total auxin concentrations.

To do so, we consider a single file of PAT cells. Each cell in the file can be given a number according to its position (order) in the file. Cell 1 starts the file at the apical side, while cell N ends the file at the basal side of the stem segment. The internal total auxin concentration in an intermediate cell n is denoted by $a_{n}\left(x\right)$ at internal position x from the cell's apical side at x=0. Its basal side is located at x=l. We assume, as in the MK‐model, that all PAT cells in the model have equal length l (~ 80 μm). The total auxin flux density $J_{n}^{b}$ at the basal side of the membrane of cell n into the apoplast connecting to the next, cell *n*+1, depends on the total auxin concentration $a_{n}^{b}=$ $a_{n}\left(l\right)$ in the cytoplasm at the basal side and the total auxin concentration $A\left(0\right)$, say, in the apoplast at the other side of this membrane part (Box 2, Panel B). One may write, for technical convenience,

(3) $$J_{n}^{b}=\beta_{1}a_{n}^{b}-\beta_{2}\;A\left(0\right),$$

where $\beta_{1}$ and $\beta_{2}$ can be interpreted as unidirectional (‘total’) permeability constants. Since the fraction of IAA in anion form in cytoplasm, $f_{c}$, and apoplast, $f_{a}$, can be computed from the acidity constant as

(4) $$f_{c}=\frac{K_{a}}{h_{c}+K_{a}},f_{a}=\frac{K_{a}}{h_{a}+K_{a}},$$

where $h_{c}$ and $h_{a}$ are the corresponding proton concentrations in these parts, $\beta_{1}$ and $\beta_{2}$ can be expressed in terms of $f_{c}$, $f_{a}$, ${\bar{P}}_{\mathrm{AH}}$ and $P_{A^{-}}^{b}$ and the membrane potential $V_{\mathrm{PM}}$ by comparison of Equation (3) with the expression for the total flux density J in Box 2, Panel A. The results are presented in Box 2, Panel C (top row, right column). Similar results are obtained for the flux density from apoplast into cell *n*+1, described by permeabilities $\beta_{3}$ and $\beta_{4}$. These permeabilities also characterize the flux of auxin from a cell in the PAT file (viewed as part of the U‐compartment in the BHL‐model) into the surrounding tissue (viewed as part of the W‐compartment), since we consider the apical and radial part of the plasma membrane as similar when auxin transport is concerned.

The link between the microscopic model at the scale of the apoplastic interface that we described above and the BHL‐model is provided by the MK‐model. It gives a description of PAT at the intermediate (‘mesoscopic’) scale of single PAT cells. Instead of considering an intermediate apoplast, Mitchison (1980) ignores the apoplast and describes the total resulting auxin flux $J_{\mathrm{tot}}$ from cell n to the next in a PAT file immediately in terms of the total auxin concentrations $a_{n}^{b}$ at the basal end of cell n and $a_{n+1}^{a}$ at the apical side of cell *n*+1:

(5) $$J_{\mathrm{tot}}=pa_{n}^{b}+q\left(a_{n+1}^{a}-a_{n}^{b}\right).$$

The key MK‐model parameters p and q can be interpreted as follows: p represents the increased permeability of the basal membrane, supposedly by the presence of PIN1 proteins, while q represents the ‘diffusive’, non‐polar part of the transport. Mitchison (1980) derived an expression for the resulting effective transport velocity, $V_{\mathrm{eff}}$, and the p and q parameters, supposing that all PAT cells in the file have equal length l and that auxin is transported within each cell by diffusion, with diffusion constant D. Kramer (2002) computed an expression for the effective diffusion at macroscopic scale, $D_{\mathrm{eff}}$. $V_{\mathrm{eff}}$ and $D_{\mathrm{eff}}$ correspond with the auxin transport velocity V and diffusion constant $D_{u}$ in the U‐compartment, respectively. So, these expressions allow us to bridge the scale from mesoscopic to macroscopic.

We recomputed these expressions by different arguments to double‐check their validity as they are key in bridging scales. We arrived at the same expression for $V_{\mathrm{eff}}$, while a slightly modified expression was obtained for $D_{\mathrm{eff}}$ compared to Kramer's result (see supplemental material, Section 4.1, for derivation and details):

(6) $$V=V_{\mathrm{eff}}=\frac{p}{p+2q+\frac{2D}{l}}∙\frac{2D}{l},D_{u}=D_{\mathrm{eff}}=\frac{q}{\frac{1}{2}p+q+\frac{D}{l}}∙D.$$

The BHL‐model parameters V and $D_{u}$ are equated to these effective velocity and diffusion constants computed from the mesoscopic MK‐model, as indicated. The expressions have been written in such a mathematical form, that the first factor in both is at most 1. Thus, the maximal transport velocity is 2D/l, while the maximal diffusivity in the U‐compartment is that inside the PAT cells, D. Since a clear view on the detailed tissue structure of the part of the vascular bundle that constitutes the W‐compartment is lacking, no upscaling has been attempted towards exhibiting the BHL‐model parameters $D_{w}$ and $D_{y}$ in terms of lower scale parameters.

Assuming that auxin diffuses within the apoplast and that a steady gradient is established there during PAT, we derived expressions for p and q in terms of the microscopic unidirectional permeability constants $\beta_{1}$, $\beta_{2},\beta_{3}$ and $\beta_{4}$ that are key to our further results:

(7) $$p=\frac{\beta_{1}\beta_{3}-\beta_{2}\beta_{4}}{\beta_{2}+\beta_{3}},q=\frac{\beta_{2}\beta_{4}}{\beta_{2}+\beta_{3}}.$$

(See supplemental material for derivation of these expressions). After some cumbersome algebra (supplementary material), an interesting expression for p, i.e. the increased permeability for auxin at the basal side, is obtained that opens opportunities for interpretation of changes in terms of microscopic physical and physiological processes:

(8) $$p=p_{\max}∙\frac{\left(\;P_{A^{-}}^{b}/P_{A^{-}}^{a}\right)-1}{\left(\;P_{A^{-}}^{b}/P_{A^{-}}^{a}\right)+1}∙\frac{\varepsilon }{M+\varepsilon }.$$

It expresses p as a maximal value $p_{\max}$ that is modulated by the two other factors, which are at most 1, and which shall be described in more detail below. In Equation (8) – notably – the maximal increased permeability

(9) $$p_{\max}={\bar{P}}_{\mathrm{AH}}\;\left[e^{\mathrm{EF}/\mathrm{RT}}-\frac{h_{c}}{h_{a}}\right]\;\frac{h_{a}}{h_{c}+K_{a}}$$

is controlled by three factors. The first is the apparent permeability for the protonated form, which – recall – contains the contribution of the symporters of the AUX1/LAX1‐3 family. The second factor at the right‐hand side of Equation (9) is related to the total proton motive force (PMF). It is positive when $e^{\mathrm{EF}/\mathrm{RT}}>h_{c}/h_{a}$. This is equivalent to the condition:

(10) $$\frac{\mathrm{RT}}{F}\ln\left(\frac{h_{a}}{h_{c}}\right)-V_{\mathrm{PM}}>0$$

The expression on the left‐hand side of Equation (10) is that of the PMF. It is the driving force of the unidirectional transport term governed by p. The third factor represents the effect of the difference in acidity between cytoplasm and apoplast.

The two factors in Equation (8) that further determine p, hence the auxin transport velocity V through Equation (6), refer to the effect of increased basal membrane permeability $P_{A^{-}}^{b}$ relative to the apical and radial permeability for the auxin anion. If there is no such increase, i.e. when $P_{A^{-}}^{b}=P_{A^{-}}^{a}$, this middle factor is zero, and consequently also p. Then, there is no polar auxin transport, consistent with the chemi‐osmotic theory. The third factor in Equation (8), of Monod type in ε, defined by:

(11) $$\varepsilon =\frac{\mathrm{EF}}{\mathrm{RT}}\;\frac{e^{-\mathrm{EF}/\mathrm{RT}}}{1-e^{-\mathrm{EF}/\mathrm{RT}}},$$

expresses the modulating effect of the membrane potential on p, hence V. The Monod constant M is given by:

(12) $$M=\frac{h_{a}}{K_{a}}\;\frac{2{\bar{P}}_{\mathrm{AH}}}{P_{A^{-}}^{b}+P_{A^{-}}^{a}}=\frac{h_{a}}{K_{a}}\;\frac{2\left({\bar{P}}_{\mathrm{AH}}/P_{A^{-}}^{a}\right)}{\left(P_{A^{-}}^{b}/P_{A^{-}}^{a}\right)+1\;}.$$

It is the value for ε at which the Monod function takes the value ½.

**FIGURE 3:**
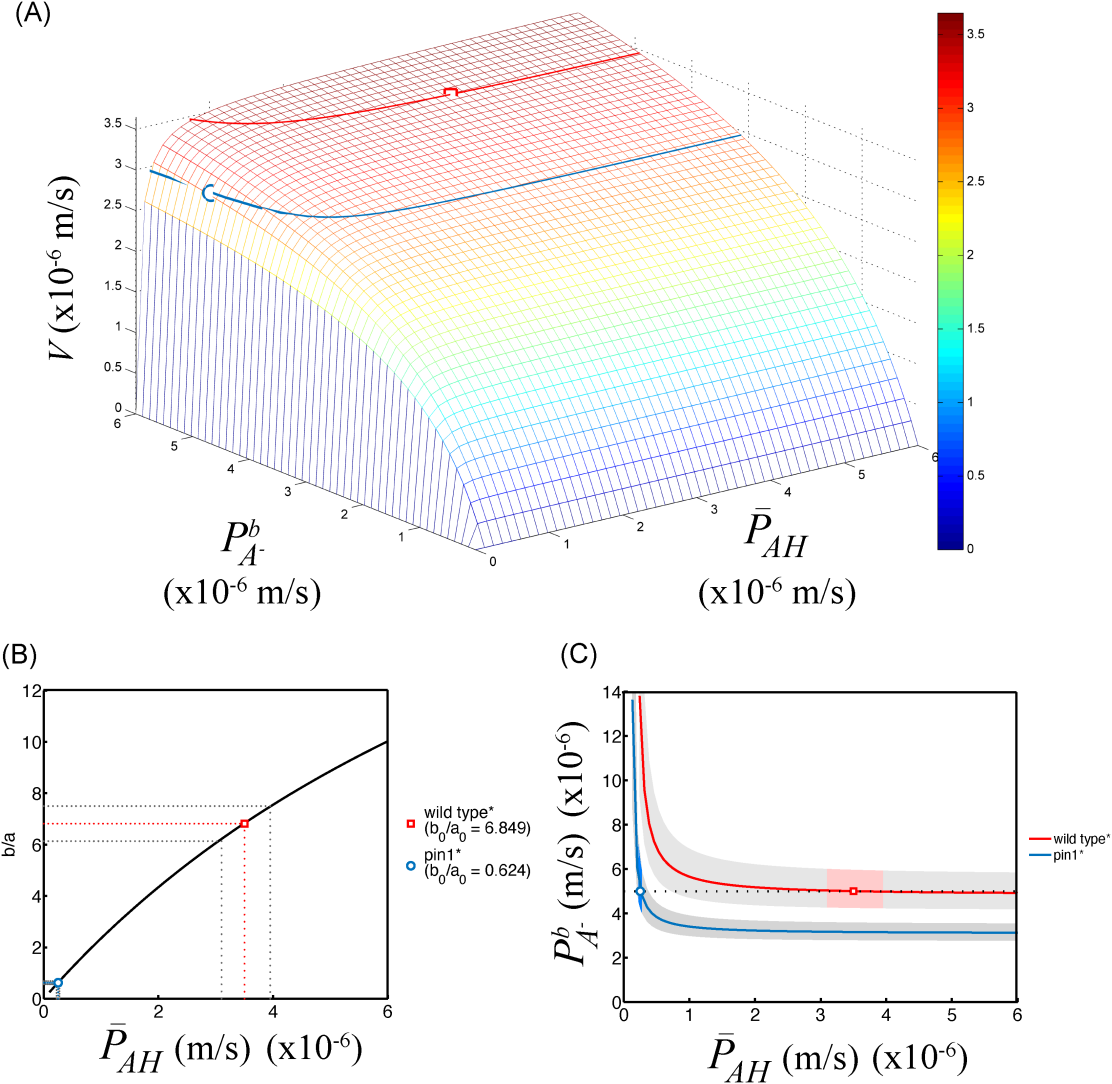
**Graphical representations of parameter relationships between the LPTF and BHL model**. The microscopic parameters ${\bar{P}}_{\mathrm{AH}}$ and $P_{A^{-}}^{b}$ in the LPTF model map to the macroscopic parameters V, a, b and $D_{u}$ in the BHL model by means of the equations exhibited in Box 2, Panel C. Parameters a and b depend both simply linearly on ${\bar{P}}_{\mathrm{AH}}$ only. Therefore, their dependence is not shown. (A) depicts the dependence of V on both ${\bar{P}}_{\mathrm{AH}}$ and $P_{A^{-}}^{b}$. Note the monotonic and saturating behavior. The fit for the selected average wild‐type plant is indicated with a red square. The one for the *pin1* mutant by a blue circle. The corresponding parameter values are listed in Table 2 (indicated with *). For each, the V‐level set, i.e. the curve of all triples $\left({\bar{P}}_{\mathrm{AH}},P_{A^{-}}^{b},V\right)$ that have the same V‐value as these two fits, is indicated by the same color (wild type: $V_{0}$ = 3.45 x 10^−6^ ms^−1^; *pin1*: $V_{0}$ = 2.92 x 10^−6^ ms^−1^). (B) The ratio b/a depends on ${\bar{P}}_{\mathrm{AH}}$ only, not on $P_{A^{-}}^{b}$. This dependence, shown as black curve, is the same for wild type and *pin1* mutant plant, because the auxiliary parameters are assumed the same for both types. It is approximately a Monod function, in the range where it is almost linear and not yet saturated. The uncertainty in the ratio b/a is that of a and b added together, i.e. 10%. These uncertainty boundaries are indicated by grey dotted lines around the fitted values $b_{0}/a_{0}$ for the wild type plant (red) and the *pin1* mutant (blue). For wild type, ${\bar{P}}_{\mathrm{AH}}$ is in the range 3.1–3.95 x 10^−6^ ms^−1^. For the *pin1* mutant this range is 2.2–2.7 x 10^−7^ ms^−1^. **C** shows the V‐level sets of **A** in a two‐dimensional graph, i.e. the collection of $\left({\bar{P}}_{\mathrm{AH}},P_{A^{-}}^{b}\right)$ value‐pairs that yield the same V‐value. In grey the zone is indicated that corresponds to 5% uncertainty in the V‐value. The possible $\left({\bar{P}}_{\mathrm{AH}},P_{A^{-}}^{b}\right)$ value‐pairs in the LPTF that correspond to the fitted $V_{0}$, $a_{0}$ and $b_{0}$ in the BHL‐model within the 5% uncertainty domain for each of V, a and b – the so‐called *solution space* – is indicated for wild type (light red) and *pin1* mutant plant (light blue). Notice that the apparent permeability ${\bar{P}}_{\mathrm{AH}}$ must have dropped in value by an order of magnitude, while $P_{A^{-}}^{b}$ remains in the same range, centered at 5 x 10^−6^ ms^−1^, when wild type is compared to *pin1* mutant.

Equations (6), (8), (9), (11) and (12) taken together (Box 2, Panel C), show the complex way in which the various constituents of the chemi‐osmotic theory, i.e. modified membrane permeabilities, acidity differences and membrane potential, determine quantitatively the observable polar auxin transport velocity at the macroscopic scale of the stem segment. In Figure 3A, the dependence of V on the membrane permeabilities $P_{A^{-}}^{b}$ and ${\bar{P}}_{\mathrm{AH}}$ has been illustrated. In contrast to the complex expressions presented above, the shape of the graph is strikingly simple.

The key permeabilities $P_{A^{-}}^{a}$, $P_{A^{-}}^{b}$ and ${\bar{P}}_{\mathrm{AH}}$ can be interpreted in terms of abundance of auxin carriers of different types at the distinguished parts of the membrane. Note that only the relative values of permeabilities matter, relative to $P_{A^{-}}^{a}$, except for the absolute value of the apparent permeability ${\bar{P}}_{\mathrm{AH}}$ for the protonated form in Equation (9). Also, the acidity in apoplast and cytoplasm only matters through their values relative to the IAA acidity constant $K_{a}$, i.e. through $h_{a}/K_{a}$ and $h_{c}/K_{a}$.

So far, we have focused on the description of the bridging‐of‐scales as far as the longitudinal flux of auxin is concerned. The radial flow of auxin, between U‐ and W‐compartment in the BHL‐model now requires attention (Box 1, Panel B). In that model it is characterized by exchange rate parameters a and b (Box 1, Panel C). In the LPTF, the radial flux *density* $J^{\mathrm{rad}}$ (mol/m^2^s) at cellular level in a PAT cell file is determined by the total cytoplasmic auxin concentration and total apoplastic concentration by the unidirectional total permeability constants $\beta_{3}$ and $\beta_{4}$ as presented in Box 2, Panel B. This ‘lifts’ directly to the scale of the macroscopic BHL‐model (supplementary material), with the U‐compartment being the collective of PAT cell files and the W‐compartment their complement within the vascular bundles (recall Box 1, Panel A and B):

$$J^{\mathrm{rad}}=\beta_{4}\;\left[U\right]-\beta_{3}\left[W\right].$$

The total amount of auxin that crosses the U/W‐barrier per unit time (mol/s) at position x from the donor side of the stem segment in a very thin slice of thickness Δx then equals $J^{\mathrm{rad}}∙S^{o}\Delta x$, where $S^{o}$ is the length of the contour in the cross section that separates the two compartments. The U‐compartment in this slice has volume $S_{u}\Delta x$, while the W‐compartment has volume $S_{w}\Delta x$. Thus, the net change per unit time in concentration in the U‐compartment in the slice equals

(13) $$\frac{J^{\mathrm{rad}}∙S^{o}\Delta x}{S_{u}\Delta x}=\beta_{4}\;\frac{S^{o}}{S_{u}}\left[U\right]-\beta_{3}\frac{S^{o}}{S_{u}}\left[W\right].$$

Comparison of Equation (13) with the radial exchange part of the equation for $\left[U\right]$ in the BHL model (see Box 1, Panel B) yields

(14) $$a=\beta_{4}\;\frac{S^{o}}{S_{u}},b=\beta_{3}\frac{S^{o}}{S_{w}}$$

(see Box 2, Panel C). Thus, through Equation (14) the LPTF links the macroscopic key model parameters a and b to the microscopic key parameters ${\bar{P}}_{\mathrm{AH}}$ and $P_{A^{-}}^{a}$, through the expressions for $\beta_{3}$ and $\beta_{4}$ in the latter parameters. (Note that radial transport is governed by the same parameter $P_{A^{-}}^{a}$)

### 3.3 Interpretation of new PAT data from *pin1* mutants in view of the multiscale LPTF

In order to put the multiscale LPTF to use, we slightly modified the experimental design and fitting protocol as compared to the previous experimental set up and analysis as described in Boot et al. (2016). This was done to increase the estimation accuracy while increasing the computational efficiency by reducing the number of experiments that must be fitted to the LPTF. The modification entailed three changes:

1. We now routinely estimated the relative amount of immobilized auxin in an ISS experimentally, by running in each PAT assay experiment parallel batches of comparable ISS, which at the end of the incubation were used for IAA extraction and thin‐layer chromatography (TLC) analysis (see M&M). This procedure provided extra data of a type different from that of transport and tissue profile, enforcing an additional constraint on the quality of fit. This enabled a better estimation of model parameters, e.g. that of auxin retention due to immobilization (i.e. immobilization rates $\kappa_{1}$ and $\kappa_{2}$).
2. The statistical analysis performed previously (Section 3.1) used model‐based parameter estimates from individual ISSs. It was found empirically by Boot et al. (2016), that these parameter values, if averaged over the stem segments of a batch, were close to the corresponding values obtained from simulations of so‐called pooled data, defined as the anatomical and transport data summed over all stem segments in a batch. We found that this form of data reduction was very useful for comparing PAT data, since it at once produced parameter values that are representative for all (usually nine) stem segments in a batch. Therefore, we made it an integral part of data fitting.
3. The fitting procedure of data in the LPTF was modified slightly with respect to that of the BHL‐model. Starting point is a set of parameter values in the BHL‐model that are characteristic of a wild type plant, for example those found in Boot et al. (2016). These were then manually tuned to provide a visually good fit to the data. Thus, practically, we implemented in the procedure the underlying idea that a mutant is a deviation of some sort from the wild type. Because the BHL‐model is used in this first step, V, $D_{u}$, a and b are allowed to vary independently. As a second step, values for the microscopic key parameters ${\bar{P}}_{\mathrm{AH}}$ and $P_{A^{-}}^{b}$ were determined that yield the obtained V, a and b, using the mathematical expressions of the LPTF, shown in Box 2, Panel C.

Note that in this procedure, the microscopic parameters are not used to determine $D_{u}$ through equation (6). We have found empirically that a change in $D_{u}$ could be compensated to some extent by a change in $P_{\mathrm{ru}}^{-}$, which was not apparent in non‐dimensionalisation of the model. It was therefore unexpected and its cause needs further mathematical investigation in future. Therefore, the diffusion constant $D_{u}$ was not fixed and, together with $D_{w}$ and $D_{y}$, it was fitted freely. Expression (6) did give a starting value for $D_{u}$, however.

We used fixed plausible values for the auxiliary parameters: the membrane potential (−120 mV, Raven 1967), cytoplasmic and apoplastic pHs (of 7 and 5, respectively), diffusivity D of IAA in the cytoplasm (2 x 10^−10^ m^2^/s), the average length of PAT cells l (80 μm) and $P_{A^{-}}^{a}$ (1.5 x 10^−8^ m/s). The latter value was used in Boot et al. 2016, also see its supplementary files for argumentation for this value. The computation of a and b from Equation (14) requires a choice for the unknown $S^{o}$, the total length of the interface between U‐ and W‐compartment in a cross section. We take the minimal length for a given enclosed area $S_{u}$, i.e. the length of a circle with area $S_{u}$: $S^{o}=2\sqrt{\pi S_{u}}$. Then, after manually initializing the fitting, automated optimization of the cost‐function was done on the COMSOL‐MATLAB coupled software platform as before and the corresponding microscopic parameters were determined.

To illustrate this mapping of microscopic LPTF parameters to macroscopic BHL parameters we present a numerical example in Table 1. For this example, we took a wild‐type reference batch of ISS and a batch of ISS from a *pin1* mutant line from the new series of PAT donor‐receiver assays. These two served as a running example in the further analysis. In Table 1 from left to right we see the complete set of microscopic parameters that determine the parameters β_1_ to β_4_, which in their turn determine the mesoscopic parameters p and q, which finally give the values for $V_{\mathrm{eff}}$ and $D_{\mathrm{eff}}$ through Equation (6). In the BHL‐model, the advection‐velocity V is then equated to $V_{\mathrm{eff}}$, while $D_{\mathrm{eff}}$ is used as initial value for the diffusion constant $D_{u}$, which is otherwise freely fitted. In addition, the macroscopic exchange parameters a and b are determined by $\beta_{4}$ and $\beta_{3},$ respectively.

**TABLE 1:** **Example of mapping of LPTF‐model parameters to BHL‐model parameters**. For wild type and *pin1* ISS it is shown from left to right how parameter values change with model and spatial scale according to the formulas presented in Box 2, starting from LPTF model parameters. These consist of the complete set of fixed microscopic parameters and the variable parameter $P_{\mathrm{AH}}$. These determine the auxiliary microscopic parameters $\beta_{1}$‐ $\beta_{4}$, which in their turn determine the mesoscopic parameters p and q of the MK‐model. These finally give the parameter values for $V_{\mathrm{eff}}$ and $D_{\mathrm{eff}}$, and the connected advection‐velocity V and the diffusion constant $D_{u}$ of the BHL‐model. In addition, the macroscopic exchange parameters a and b in the BHL‐model are determined by $\beta_{4}$ and $\beta_{3}$ respectively.

LPTF parameters (microscopic)	Auxiliary (microscopic)	MK parameters, (mesoscopic)	BHL parameters (macroscopic)
**Wild type**			
E = *z*V_PM_ = 120 mV	β_1_ = 2.34 x 10^−5^ ms^−1^		$V=V_{\mathrm{eff}}=$ 3.45 x 10^−6^ ms^−1^
pH_cyt_ = 7.0	β_2_ = 1.48 x 10^−6^ ms^−1^	*p* = 1.14 x 10^−5^ ms^−1^	$D_{u}=D_{\mathrm{eff}}=$ 1.19 x 10^−12^ m^2^s^−1^
pH_apo_ = 5.0	β_3_ = 1.35 x 10^−6^ ms^−1^	*q* = 4.88 x 10^−8^ ms^−1^	*a* = 4.44 x 10^−4^ s^−1^
$P_{A^{-}}^{a}$ = 1.5 x 10^−8^ ms^−1^	β_4_ = 9.35 x 10^−8^ ms^−1^		*b* = 3.03 x 10^−3^ s^−1^
$P_{A^{-}}^{b}$= 5 x 10^−6^ ms^−1^			
${\bar{P}}_{\mathrm{AH}}$ = 3.5 x 10^−6^ ms^−1^			
***pin 1***			
E = *z*V_PM_ = 120 mV	β_1_ = 2.39 x 10^−5^ ms^−1^		$V=V_{\mathrm{eff}}=$ 2.94 x 10^−6^ ms^−1^
pH_cyt_ = 7.0	β_2_ = 2.23 x 10^−7^ ms^−1^	*p* = 7.25 x 10^−6^ ms^−1^	$D_{u}=D_{\mathrm{eff}}=$ 1.64 x 10^−12^ m^2^s^−1^
pH_apo_ = 5.0	β_3_ = 9.79 x 10^−8^ ms^−1^	*q* = 5.07 x 10^−8^ ms^−1^	*a* = 3.26 x 10^−4^ s^−1^
$P_{A^{-}}^{a}$ = 1.5 x 10^−8^ ms^−1^	β_4_ = 7.29 x 10^−8^ ms^−1^		*b* = 2.05 x 10^−4^ s^−1^
$P_{A^{-}}^{b}$= 5 x 10^−6^ ms^−1^			
${\bar{P}}_{\mathrm{AH}}$ = 2.5 x 10^−7^ ms^−1^			

**FIGURE 4:**
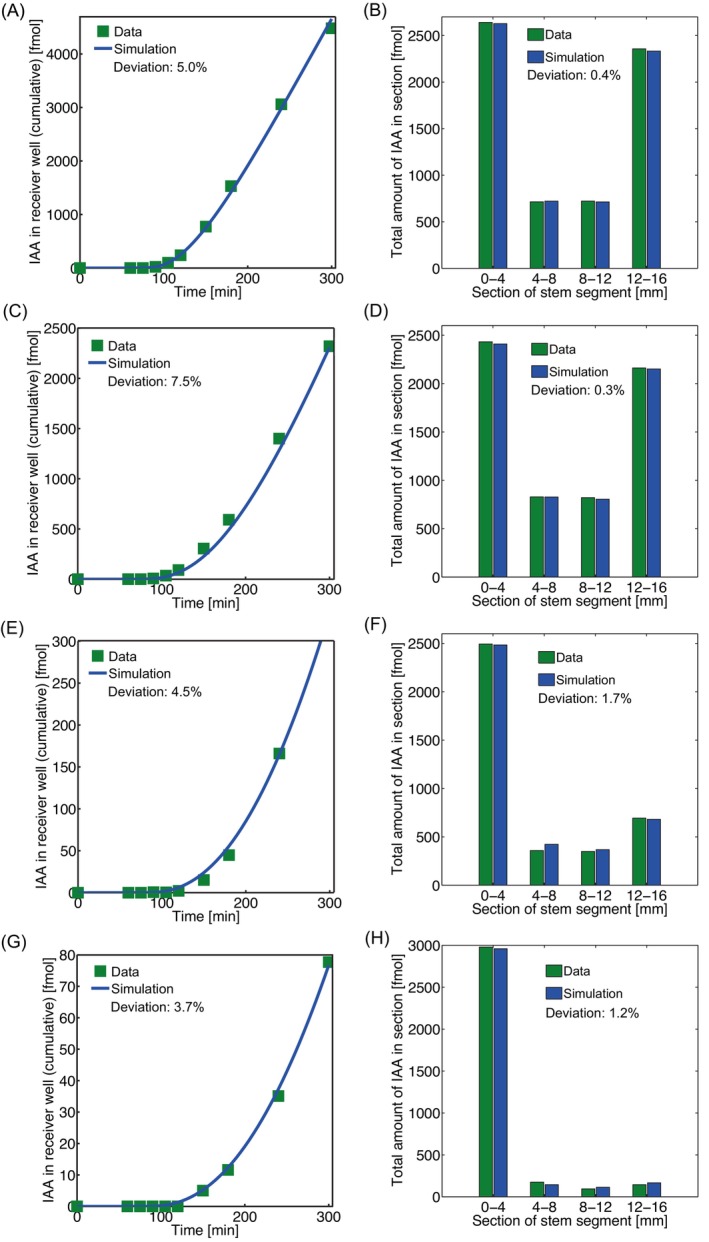
**Examples of quality of fit of BHL‐model to various data sets**. Representative efflux and tissue profiles for pooled data and their simulations with the BHL‐ model for (A,B) wild‐type, (C,D) pML1::PIN1:GFP/pin1 line A, (E,F) pML1::PIN1:GFP/pin1 line B and (G,H) pin1 mutant plant lines. Parameter settings can be found in Table 2(A), denoted by an asterix *) and in the legend of Table 2.

Our above example was taken from a more extensive set of PAT assays in which we compared the analysis of PAT data from wild‐type plants with those from *pML1::PIN1:GFP/pin1* and *pin1* mutants. Only the values of the most important parameters are shown, with a summary of the results in terms of the average parameter values at the bottom panel of Table 2. The results of a representative selection of a set of experiments are shown in Figure 4.

**TABLE 2:** **Overview changes in of main model parameters between wild type, pin1 and aux1/lax1‐3 mutant lines**. Parameter settings were used to simulate pooled PAT data from wild‐type, *pML1::PIN1:GFP/pin1* line A and B and *pin1* (A) and the *aux1/lax1‐3* quadruple mutant (B) with the BHL model with indicated total fitting cost. Obtained values for $a_{0}$ *,* $b_{0}$ *,*and $V_{0}$ yielded the values for ${\bar{P}}_{\mathrm{AH}}$ and $P_{A^{-}}^{b}$ from the LPTF model. Additional parameter settings, which were the same for all the lines analyzed, were *D*=2 x 10^−10^ m^2^s^−1^, *D*_*w*_=7x 10^−10^ m^2^s^−1^,*D*_*y*_=2 x10^−11^ m^2^s^−1^, *κ*_2_=1 x10^−6^ s^−1^, *α*=32%, *E*=120 mV, $P_{A^{-}}^{a}$ =1.5 x 10^−8^ ms^−1^, pH_cyt_ = 7.0 and pH_apo_ = 5.0. $C_{d}$ and anatomical parameters differed between sets. The lower part of (A) gives a summary of the average parameter values. ‘*’ denotes selected plants and parameter values used in Figure 3 (fit of wild type and *pin1* mutant), the simulation of transport data in Figure 4 and sensitivity analysis in Figure 5.

(A)	${\bar{P}}_{\mathrm{AH}}$	$P_{A^{-}}^{b}$ (ms^−1^)	$V_{0}$	$a_{0}$	$b_{0}$	$b_{0}/a_{0}$	$D_{u}$	$P_{\mathrm{du}}^{+}$	${\;P}_{\mathrm{dw}}^{+}$	κ_1_	$P_{\mathrm{ru}}^{-}$	Immob.	Cost	*D*_*u*_ (m^2^s^−1^)
	(x 10^−6^ ms^−1^)	(x 10^−6^ ms^−1^)	(x 10^−6^ ms^−1^)	(x 10^−3^ s^−1^)	(x 10^−3^ s^−1^)		(x 10^−12^ m^2^s^−1^)	(x 10^−6^ ms^−1^)	(x 10^−6^ ms^−1^)	(x 10^−5^ s^−1^)	(x 10^−10^ ms^−1^)			
Wild type	3.5	5.0	3.45	0.444	3.03	6.82	1.19	1.55	1.80	1.11	3.24	13%	7.0%	1.19x10^−12^
Wild type*	3.5	5.0	3.45	0.443	3.04	6.86	1.19	5.00	1.20	0.81	3.46	10%	5.3%	1.19x10^−12^
Wild type	1.4	5.0	3.38	0.336	1.07	3.18	1.13	0.30	0.77	2.00	3.70	18%	3.2%	1.13x10^−12^
*pML1::PIN1:GFP/pin1* line B	0.25	5.0	2.92	0.359	0.224	0.624	1.66	1.90	1.00	3.77	2.45	18%	7.9%	1.66x10^−12^
*pML1::PIN1:GFP/pin1* line B*	0.30	5.0	3.01	0.375	0.280	0.747	1.55	1.26	0.50	2.53	2.94	12%	5.9%	1.55x10^−12^
*pML1::PIN1:GFP/pin1* line A*	0.70	5.0	3.27	0.386	0.649	1.68	1.23	4.70	0.90	1.39	3.49	11%	6.2%	1.23x10^−12^
*pin1*	0.11	5.0	2.41	0.321	0.090	0.280	2.33	0.52	0.153	10.9	13.6	29%	5.8%	2.33x10^−12^
pin1*	0.25	5.0	2.92	0.327	0.204	0.624	1.66	0.20	0.219	4.78	3.66	18%	5.0%	1.66x10^−12^
**Average**	${\bar{P}}_{\mathrm{AH}}$	$P_{A^{-}}^{b}$	$V_{0}$	$a_{0}$	$b_{0}$	$b_{0}/a_{0}$	$D_{u}$	$P_{\mathrm{du}}^{+}$	$P_{\mathrm{dw}}^{+}$	κ_1_	$P_{\mathrm{ru}}^{-}$	Immob.		*D*_*u*_
Wild type	**2.8**	**5.0**	**3.43**	**0.409**	**2.38**	5.82	1.17	2.28	1.26	1.31	3.47	13.7%		1.17x10^−12^
*pML1::PIN1:GFP/pin1*	**0.4**	**5.0**	**3.07**	**0.373**	**0.38**	1.02	1.48	2.62	0.80	2.56	2.96	13.7%		1.48x10^−12^
*pin1*	**0.2**	**5.0**	**2.67**	**0.324**	**0.15**	0.463	1.99	0.36	0.19	7.84	8.63	23.5%		1.99x10^−12^
	**0.2**	**5.0**	**2.81**	**0.275**	**0.14**	0.509	1.81	0.72	0.14	12.9	5.62	38%	9.8%	1.81x10^−12^
**(B)**														
*aux1/lax1‐3* quadruple														

**FIGURE 5:**
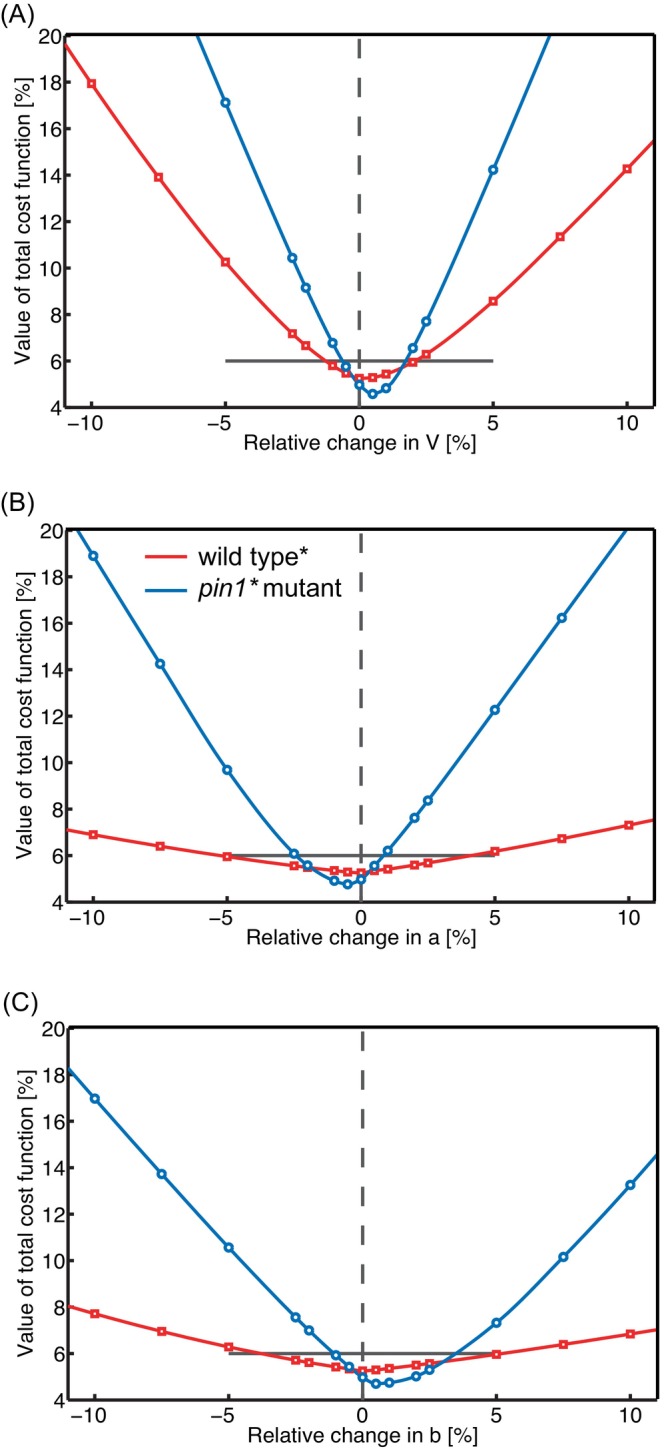
**Parameter sensitivity in the BHL model**. The effect on the value of the total cost function (vertical) is shown of varying the parameter values of the key parameters V (A), a (B) and b (C) in the BHL model separately, relatively around the fitted value, for a selected wild‐type plant and *pin1* mutant (both pooled data, selected batch indicated by * in Table 2). Fitted values around which perturbations are made: for wild type, $V_{0}$ = 3.45 x 10^−6^ ms^−1^, $a_{0}$ = 4.43 x 10^−4^ s^−1^, $b_{0}$ = 3.04 x 10^−3^ s^−1^; for *pin1* mutant: $V_{0}$ = 2.92 x 10^−6^ ms^−1^, $a_{0}$ = 3.27 x 10^−4^ s^−1^, $b_{0}$ = 2.04 x 10^−4^ s^−1^. The horizontal black line shows the horizon of acceptability of a fit, i.e. a total cost function value of 6%.

The presented values for ${\bar{P}}_{\mathrm{AH}}$ and $P_{A^{-}}^{b}$ in Table 2 should be interpreted in the following way, which we illustrate for the wild‐type plant and *pin1* mutant indicated by an asterisk in that table: first, we performed a sensitivity analysis of the dependence of the total cost function on the parameters V, a and b around the obtained value $\left(V_{0},a_{0},b_{0}\right)$, for which the total cost function was below the chosen threshold of acceptability of 6% (Figure 5). It shows, that other values could have been obtained for these parameters that deviate by at most ca. 5% from $\left(V_{0},a_{0},b_{0}\right).$ The ratio b/a is interesting, because it depends on ${\bar{P}}_{\mathrm{AH}}$ only, while the anatomical parameter $S^{0}$, which is difficult to determine, is not involved:

(15) $$\frac{b}{a}=\frac{S_{u}}{S_{w}}\;\frac{\beta_{3}}{\beta_{4}}=\frac{S_{u}}{S_{w}}∙\frac{{\bar{P}}_{\mathrm{AH}}\;\left(1-f_{a}\right)+f_{a}{\;P}_{A^{-}}^{a}\;\varepsilon \;}{{\bar{P}}_{\mathrm{AH}}\;\left(1-f_{c}\right)+f_{c}\;P_{A^{-}}^{a}\;\varepsilon \;e^{\mathrm{EF}/\mathrm{RT}}}$$

As a function of ${\bar{P}}_{\mathrm{AH}}$, it has approximately the shape of a Monod function, in the part where it is still almost linear and not yet saturated (Figure 3B). Its shape is the same for the wild‐type plant and *pin1* mutant, because the auxiliary parameters are assumed the same.

Since b/a depends on both a and b, its value may deviate by at most 10% from the obtain value $b_{0}/a_{0}$. These margins have been indicated by dotted grey lines in Figure 3B and yield a range of acceptable values for ${\bar{P}}_{\mathrm{AH}}$. For the wild‐type plant this is 3.1–3.95 x 10^−6^ ms^−1^, while for the *pin1* mutant it is 2.2–2.7 x 10^−7^ ms^−1^. The fitted value $V_{0}$ for V determines a level‐set in the $\left({\bar{P}}_{\mathrm{AH}},P_{A^{-}}^{b}\right)$‐parameter plane. That is, a set of pairs of values for these parameters that result in the same fixed value $V_{0}$ for V through the equations of the LPTF. As it were, one considers a horizontal slice of the graph of V as function of ${\bar{P}}_{\mathrm{AH}}$ and $P_{A^{-}}^{b}$ (Figure 3A). These level sets for the wild‐type plant and *pin1* mutant are shown in a single plot in Figure 3C. Changing $V_{0}$ by at most 5% results into the grey areas around the level‐sets for $V_{0}$ as shown. These are the regions where the $\left({\bar{P}}_{\mathrm{AH}},P_{A^{-}}^{b}\right)$ value pair can result in a V that deviates no more than 5% from the given $V_{0}$. Superimposing the obtained regions for ${\bar{P}}_{\mathrm{AH}}$ yields *solution spaces* for the pairs of values $\left({\bar{P}}_{\mathrm{AH}},P_{A^{-}}^{b}\right)$ for the wild‐type plant (shown in light red) and the *pin1* mutant (light blue).

We found that, starting from the set of wild‐type parameters, we had to make mostly a major change to the value of the apparent permeability constant of the protonated form ${\bar{P}}_{\mathrm{AH}}$, with an unaltered range for $P_{A^{-}}^{b}$, to obtain an optimal fit of the PAT data from the *pML1::PIN1:GFP/pin1* and *pin1* mutants. In the parameter values in Table 2 we noticed a decrease of ${\bar{P}}_{\mathrm{AH}}$ from 2.8 x 10^−6^ ms^−1^ in the parameter set from wild‐type plants to 0.4 x 10^−6^ ms^−1^ and 0.2 x 10^−6^ ms^−1^ in the parameter set from *pML1::PIN1:GFP/pin1* and *pin1*, respectively, while $P_{A^{-}}^{b}$ was not changed. We could not find other combinations of microscopic parameters that gave comparable good fits. These decreases were associated with a slight decrease of the values of the parameters V, $D_{u}$, and a, but a substantial decrease of parameter b from 2.38 x 10^−3^ s^−1^ in the wild‐type parameter set to 0.38 x 10^−3^ s^−1^ and 0.15 x 10^−3^ s^−1^ in *pML1::PIN1:GFP/pin1* and *pin1* parameter set, respectively. It should be noted that the value for $P_{A^{-}}^{b}$ remains in a small range around 5 x 10^−6^ ms^−1^, which does not differ substantially between the wild‐type plant and the *pin1* mutant (Figure 3C).

The macroscopic exchange rate b controls the rate of uptake of auxin from the W‐compartment into the polarly auxin transporting files of PAT cells (U‐compartment). Therefore, we tentatively conclude that the aberrant transport profiles in the *pML1::PIN1:GFP/pin1* line B and *pin1* mutants and to a lesser extent in the *pML1::PIN1:GFP/pin1* line A, as compared with those from wild‐type plants, may be due to a reduced reflux of auxin into the PAT cells from their direct environment. The net effect of this change is an apparent increase of leaked auxin in this environment. In the *pin1* mutant in particular this appeared to be associated with increased immobilization of auxin as revealed by the TLC analysis and an increase of parameter $\kappa_{1}$.

It is well established that plasma membranes of PAT cells are equipped with AUX1/LAX auxin anion/proton symport carriers which transport the auxin anion together with one proton (Yang et al., 2006; Carrier et al., 2008; Péret et al., 2012; Singh et al., 2018). It is generally believed that these carriers counteract the leak of auxin from its transport channels by reuptake of auxin. Hence, if our interpretation of the aberrant transport profiles of the *pin1* mutants is correct, we predict that the genes encoding these transporters are downregulated in the *pML1::PIN1:GFP/pin1* and *pin1* mutants or that the AUX1/LAX proteins are reduced in number, or somehow inactivated.

### 3.4 Expression of *AUX1/LAX1‐3* genes in the *pML1::PIN1:GFP/pin1* and *pin1* mutants

**FIGURE 6:**
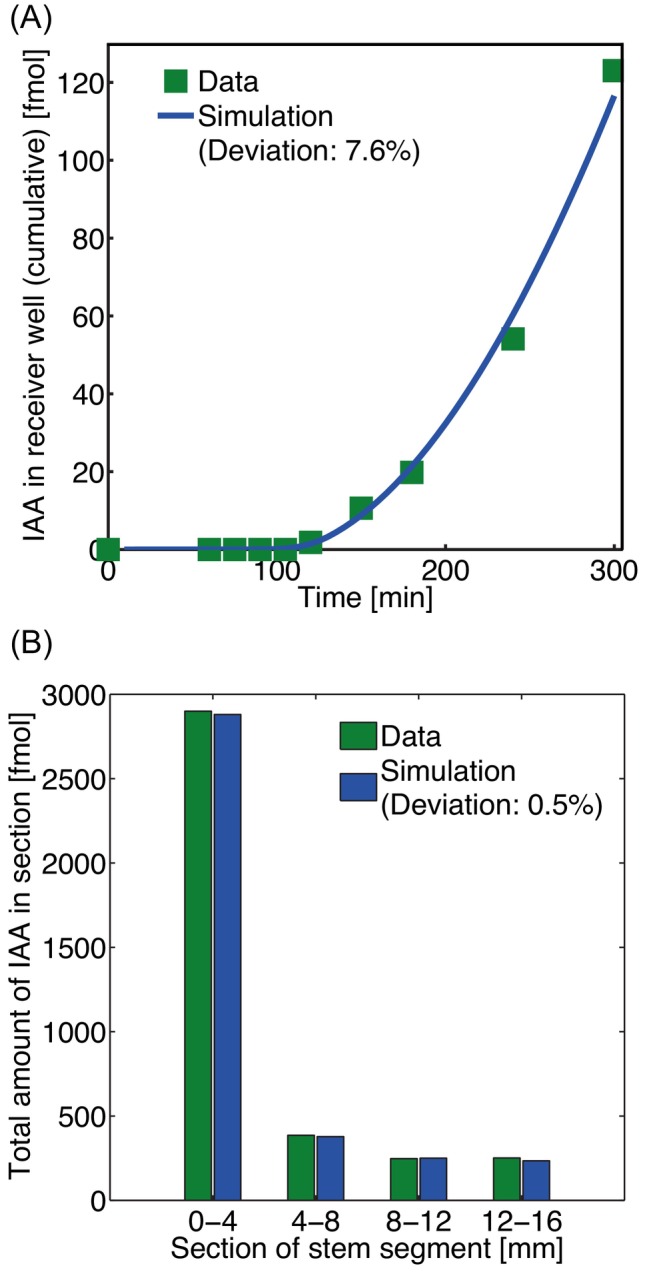
**Efflux and tissue profile and the simulation with the BHL model for an aux1/lax1‐3 quadruple mutant**. (A) shows the efflux profile and (B) the tissue profile. Parameter settings can be found in Table 2 (B) and in the legend of Table 2. Total cost 8.1%.

In order to test the above hypothesis, we estimated parameter values from an experiment using ISS from an Arabidopsis *aux1, lax1,2,3* quadruple mutant (Table 2B). This mutant, in which all four genes encoding auxin‐influx carriers were knocked out, showed a striking resemblance with the *pin1* mutant, not only with respect to the efflux and tissue profiles, but also with respect to the parameters (Figure 6, Table 2B). This observation is in line with our prediction that the aberrant efflux and tissue profiles of the *pin1* mutant are due to an inactivation of the auxin import system, as a result of either a hampered expression of the *AUX/LAX* genes or an inactivation of their protein products. We decided to investigate the former, since it could be tested quite straightforwardly, while testing the latter is technically much more complicated and is beyond the scope of the current study.

**FIGURE 7:**
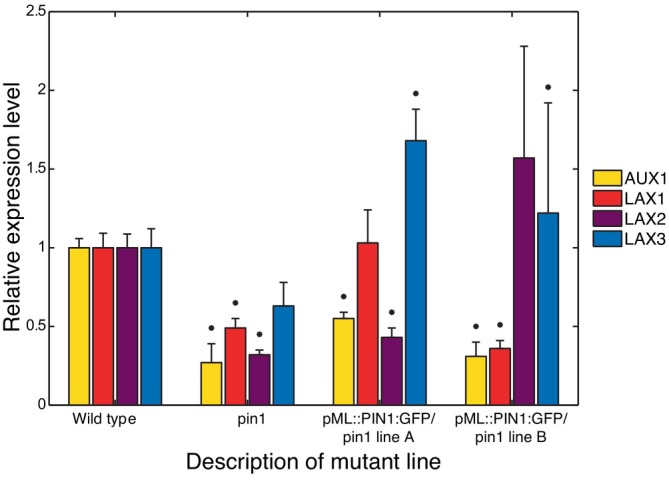
**Expression of the auxin import carrier genes AUX1, LAX1, LAX2 and LAX3 in wild‐type Arabidopsis and pin1 mutant lines**. Expression of *AUX/LAX* genes in basal inflorescence stem segments of the indicated *pin* mutant lines relative to that in wild‐type Arabidopsis as determined by qRT‐PCR. Bars indicate the mean of three independent experiments, error bars indicate the SD, and asterisks denote significant difference of the mean compared to wild type according to the unpaired two‐sided Student's t‐test (supplementary files for details).

Using RT‐qPCR analysis we measured the expression of the *AUX1/LAX1‐3* genes in basal inflorescence stem segments of *pML1::PIN1:GFP/pin1* and *pin1* mutants and compared them with those in wild‐type plants. The results show that in *pin1* mutants the expression of all four *AUX1/LAX1‐3* genes is substantially down regulated as compared to wild‐type plants (Figure 7). In Table 2 we included parameter values from two different lines of the *pML1::PIN1:GFP/pin1* mutant, denoted line A and line B. Figure 7 shows that from the four *AUX1/LAX1‐3* genes in these lines only two were expressed at wild‐type or even higher level, but in different combinations. In the B‐line the combination consisted of the *LAX2* and *LAX 3* genes, whereas in the A‐line it consisted of *LAX1* and *LAX3*. Interestingly, as is shown in Table 2, these differences appeared to be correlated with different ${\bar{P}}_{\mathrm{AH}}$ values: Average ${\bar{P}}_{\mathrm{AH}}$ line B = 3 x 10^−7^ ms^−1^, average ${\bar{P}}_{\mathrm{AH}}$ line A = 7 x 10^−7^ ms^−1^. However, both values were significantly lower than the wild‐type values, which on average were 2.8 x 10^−6^ ms^−1^. Probably, we came upon a subtle interplay of the four *AUX/LAX* genes, which is interesting, but beyond the scope of this article.

**FIGURE 8:**
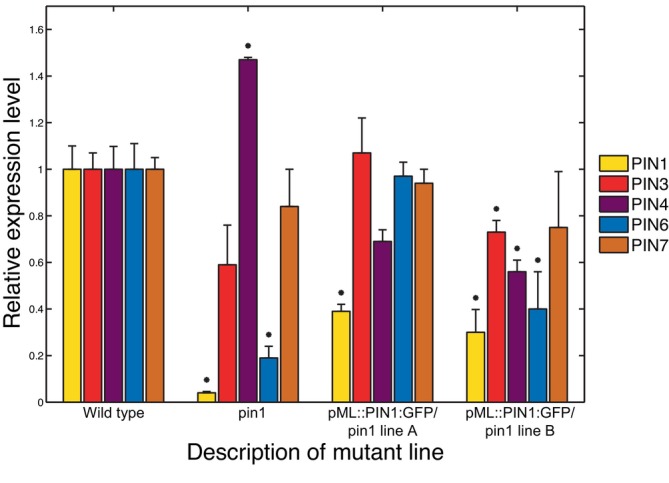
**Expression level of the PIN1, PIN3, PIN4, PIN6 and PIN7 genes in wild‐type Arabidopsis and pin1 mutant lines**. Expression of *PIN* genes in basal inflorescence stem segments of the indicated *pin* mutant lines relative to that in wild‐type Arabidopsis as determined by qRT‐PCR. Bars indicate the mean of three independent experiments, error bars indicate the SD, and asterisks denote the significant difference of the mean expression levels as compared to wild type according to the unpaired two‐sided Student's t‐test (see supplementary files for details).

In order to exclude the possibility that loss‐of‐function of the *PIN1* gene would lead to higher expression levels of other *PIN* genes, which could take over the role of *PIN1* in the *pML1::PIN1:GFP/pin1* and *pin1* lines, we studied the expression levels of *PIN1, PIN3, PIN4, PIN6* and *PIN7* genes in these mutant ISS and compared them to wild‐type plants. Except for a small increase of *PIN4* expression in the *pin1* mutant, none of the other *PIN* genes were expressed at higher levels in the mutant lines (Figure 8). The relative higher expression *of PIN1* in the *pML1::PIN1:GFP/pin1 lines* as compared to the *pin1* mutant is caused by its ectopic expression in epidermis cells from the pML1 promoter (Lu et al., 1996; Sessions et al., 1999).

## 4 DISCUSSION

Fundamental to our approach is the investigation of mathematical models, which are as simple as possible in construction yet capture the essential features necessary to reproduce the biological complexity that is observed. In this spirit, the long‐range PAT theoretical framework (LPTF), as developed in our lab over the past few years, is an attempt to understand long‐range PAT as observed under the controlled experimental conditions of a classical donor‐receiver assay using inflorescence stem segments from Arabidopsis plants. As a merger of three existing models of PAT, the macroscopic BHL‐model, the mesoscopic MK‐model and the microscopic chemi‐osmotic theory, the LPTF captures the essential features necessary to reproduce the experimental long‐range PAT data. In fact, the LPTF was developed out of necessity to understand the aberrant experimental PAT profiles from Arabidopsis *pin1* knock‐out mutant plants. These mutants differed significantly in auxin transport velocity compared to wild‐type plants and other tested mutants in the performed statistical analysis.

The mathematical structure of the LPTF exhibits the dependence of macroscopic key parameters of the BHL‐model, in particular the advection velocity V and the radial‐exchange parameters a andb, on microscopic parameters of the chemi‐osmotic theory with as intermediate step the permeability parameters p and q of the MK‐model (Box 2). We rederived by different argumentation in supplemental material, Section 4.1 the expression (6) for V in terms of mesoscopic parameters as given by Mitchison (1980), which is well‐accepted in the field. However, a reassessment of this expression for the advective velocity in the PAT cell files – and the one derived here for the effective diffusion constant – would be welcome, using the well‐established and mathematically precise technique of asymptotic analysis, similar to the work by Band & King (2012) for the root. The contribution of intracellular auxin transport in the presence of a large vacuole and possible cytoplasmic streaming then deserves particular attention. See also a discussion of these effects in Mitchison (1980), Section 5. As Band & King (2012) shows, this requires an elaborate study that allows for a much more mathematical publication on itself. So, here we felt content with the less precise derivations as provided.

In addition to microscopic parameters such as local pHs, electric membrane potential, and intracellular diffusion constants for total auxin, the most important microscopic parameters are the increased permeability of the basal plasma membrane (PM) domains of the interfaces between PAT cells for auxin anions, represented by $P_{A^{-}}^{b}$ and the *apparent* total permeability ${\bar{P}}_{\mathrm{AH}}$ for the protonated form of auxin, which is assumed to have the same value everywhere around the PM of individual PAT cells. The latter represents the physical transport of the ‘true’ protonated form, together with auxin‐anion/proton symport, which is essentially electrically neutral, resulting into mathematical expressions that appear to represent transport of the protonated form. Recall that these parameters, respectively, represent the abundance of active auxin‐anion export carriers, in particular in the shape of PIN proteins, and the basic permeability of the PM for the protonated form of auxin plus the density of transporters of the AUX1/LAX1‐3 type.

As mentioned in the Introduction, there is consensus that the AUX1/LAX1‐3 family consists of auxin‐anion transporters that operate as symporters with one or more protons. How many precisely is still under debate. Various authors (Steinacher et al. 2012, Singh et al. 2018, Dindas et al. 2018) follow the suggestion of a double‐proton symport suggested by Hertel (1983) that was investigated experimentally by Lomax et al. (1985). The latter experiments are insufficiently conclusive in our opinion to deviate from the simpler one‐proton assumption, which was originally taken by Rubery and Sheldrake (1974) in their formulation of the chemi‐osmotic theory. Moreover, Lomax et al. (1985) draw the conclusion of two‐proton symport with some reservations. No hard experimental evidence has been gathered since, to our knowledge. Kerr and Bennett (2007) state that ‘The general mechanism of transport remains to be elucidated…’. Investigation of this question of stoichiometry is hence still of interest. It lies however outside the scope of this paper. So, in modelling transport of the carriers in the AUX1/LAX1‐3 family we have assumed these to be auxin‐anion/single proton symporters.

All analysed *pin1* mutants showed characteristic auxin transport profiles, marked by relatively low advection velocities, still within the range of wild‐type advection velocities, but with substantial reduced efflux profiles. We found that the LPTF readily offered a straightforward explanation of the characteristic transport profiles of the *pin1* mutants. The dependence of auxin transport velocity V on the microscopic key parameters ${\bar{P}}_{\mathrm{AH}}$, the apparent permeability for the protonated form of IAA, and $P_{A^{-}}^{b},$ the increased permeability for the IAA anion at the basal side of PAT cells, as defined in the LPTF, has been presented in Figure 3. For each observed PAT profile, it results in a solution space of possible values for these key parameters, against a background of a set of plausible values of the other microscopic parameters. In Figure 3 we have indicated the position of a wild‐type and that of a *pin1* mutant giving good reproductions of their PAT profiles. It illustrates how we can go from wild‐type plant to *pin1* mutant in this solution space: it requires only a significant decrease of the value of ${\bar{P}}_{\mathrm{AH}}$, whereas the value for $P_{A^{-}}^{b}$ stays the same. This is what we found in all experiments we performed, of which a summary is given in Tables 2A and 2B. A most obvious explanation for the decrease of ${\bar{P}}_{\mathrm{AH}}$ was given by the assumption that the expression levels of the genes encoding the AUX1/LAX1‐3 proteins were somehow down‐regulated in the *pin1* mutants. This assumption was subsequently supported by our investigation of the expression levels of these genes in the *pin1* mutants.

Moreover, quadruple mutants of the *AUX1/LAX1‐3* genes, resulted in aberrant PAT profiles that were quantitively similar to those of the *pin1* mutants. Of course, one might argue whether in this quadruple mutant, the *PIN1* gene is downregulated or its protein product inactivated. However, we do not think this is likely, because in contrast to the *pin1* mutants, the *aux1/lax1‐3* quadruple mutant has a wild‐type phenotype.

The outcome of our analysis of the experimental PAT data, on the one hand explains the aberrant PAT profiles of *pin1* mutants, on the other hand, it raises a serious question as to the exact role of PIN1 proteins in PAT. So, let us discuss a few questions and how we may seek possible answers. In the realm of speculation, one may think of plasmodesmata as intercellular transport channels in polar auxin transport. There is indeed increasing evidence that auxin may diffuse through plasmodesmata (Han et al., 2014; Deinum et al., 2019; Paterlini 2020). However, sole diffusion is not an option for long‐distance auxin transport, since energy‐requiring advection should at least require active transport through plasmodesmata combined with differential gating to direct unidirectional polarity.

Another question is whether there may exist auxin‐anion transporters other than members of the PIN family. Interestingly, there are a few reports suggesting that members of the so‐called P‐glycoprotein multidrug resistance and ATP‐binding cassette subfamily B of putative auxin transport proteins (ABCBs), such as PGP1 and PGP19, may colocalize with members of the PIN family, such as PIN1, and act synergistically with them as auxin‐anion transporters (Blakeslee et al., 2007, Hilleary, 2022, Deslauriers and Spalding, 2021). Previously, we have shown that addition of the PAT inhibitor NPA to the donor well completely inhibits long distance PAT in inflorescence stems. Since recent structural analysis of PINs and previous studies on ABCBs indicate that NPA targets both PINs and ABCBs (Geisler and Murphy, 2006; Abdollahi Sisi and Růžička, 2020; Yang et al., 2022; Ung et al., 2022), ABCBs are the likely candidates to maintain the permeability at the basal PM.

If in our experimental system such a colocalization took place it would offer an explanation that in wild‐type plants the permeability constants of the basal PM‐domains $P_{A^{-}}^{b}$ (5x10^−6^ ms^−1^) in PAT cells for auxin‐anions has to be at least two orders of magnitude larger than the basic permeability $P_{A^{-}}^{a}$ (1.5x10^−8^ ms^−1^). If we assume that the relative contribution of these transporters varies with different physiological conditions, it is not implausible to assume that the loss of PIN1 proteins in knock‐out mutants, may be causing a kind of stress physiology that brings about an increase of the PGP's as primary ATP‐dependent, powerful transporters, to compensate for the loss of transport activity in the *pin1* mutants. As part of the screening of several *pin* mutants, we also included the *pgp1 pgp19* double mutant. The auxin transport velocity V of these mutants belonged to the lower class which is further occupied by *pin1* mutants. As distinct from the *pin1* mutant, at this stage of our research we did not select the *pgp1 pgp19* mutant for further investigation.

It is well documented that PIN1 proteins are transported by nanovesicles towards the basal PM domains of PAT cells (Adamowski and Friml, 2015, Geldner et al., 2001, Friml and Palme, 2002). However, it is still a matter of debate whether the recycling vesicles transport PINs as cargo, or that the PIN's serve as transporters to pump auxin into the vesicles, which then secrete their cargo by means of exocytosis into the apoplast of the interfaces of the PAT cells. Quite recently, Hille et al. (2018) developed a mathematical model to see whether this mode of intracellular auxin transport could in principle compete with the transport activity of PINs incorporated into the basal PM domains of PAT cells. They concluded that while secretion vesicle‐mediated transport of auxin is a theoretically possible model, it falls short in transport capacity as compared with the estimated transport capacity of PINs incorporated into the basic PM domains of PAT cells. We consider this theoretical exercise a first step for further investigation. Although we find a more detailed discussion of this model beyond the scope of the present study, we will mention at least one point that needs reassessment: the loading of the vesicles. The vesicular transport mode of auxin is analogous to the vesicular transport of neurotransmitters, of which extensive literature shows, among other features, how loading of the vesicles requires relatively high electric potentials over the vesicle membranes and, for example in the case of glutamine transporting vesicles, high‐power glutamine/H^+^ antiporters are coupled to H^+^‐ATPases driving the transport (Edwards, 2007). In this way high accumulation ratios are realized, yielding relatively high quantal size, i.e. the amount of transmitter per vesicle. This aspect was not addressed in the study by Hille et al. (2018). Perhaps we are facing the same problem as discussed above, namely that PINs as such do not have sufficient power to load the vesicles and also may require the ATP‐driven auxin transporters of the PGP type in a synergistic fashion to realize sufficiently high accumulation ratios.

In future research we will revise and incorporate the model described in Hille et al. (2018) into the LPTF, so that it can be tested on real PAT data. We are interested in a vesicle‐mediated intracellular auxin transport mode, because it may solve also a possible shortcoming of the LPTF. In the LPTF it is assumed, in accordance with the MK‐model, that intracellular auxin transport is by linear diffusion gradients. The question is: Is this real? Perhaps it is not, because intracellular mobility of organelles and stirring of the cytosol are real expressions of the intracellular dynamics that mix solutes, thus making a steady gradient impossible. Vesicles with their cargos find their way in this turmoil to their diverse intracellular targets (see Geitmann and Nebenführ, 2015).

Our interest in the intracellular transport component of PAT is further stimulated by our finding that in the green algae *Chara*, the *Charales* belong to the lineage that include the land plant, intracellular polar auxin transport does not occur by simple diffusion or cytoplasmic streaming and therefore strongly suggests that it may be by vesicle trafficking (Boot et al., 2012, Raven, 2013). A further investigation of this possible mode of intracellular auxin transport in *Chara* is in progress, in addition to the investigation of a similar type of intracellular auxin transport in land plants as outlined above.

There are quite a number of (putative) auxin transporters identified, such as members of the PIN family, members of the PGP family, and AUX1/LAX1‐3 transporters. How the expression levels and activities of these (and possibly some still unknown) transporters are coordinated and regulated undoubtfully is one of the questions that need to be resolved. Here, we have shown an interdependence of expression levels of PIN1 proteins, the expression levels of AUX1/LAX1‐3 transporters and possibly members of the PGP family. Hopefully, in the near future a deeper understanding of the exact role of these transporters in long‐range PAT will be discovered.

## AUTHOR CONTRIBUTIONS

K.J.M. B., S.C.H., K.R.L. and M.L.‐N. designed and performed the research, analysed the data and wrote the paper. O.K. performed the research. R.O. designed the research, wrote the paper and did the supervision. B.v.D. wrote the paper and did the supervision.

## Supporting information


**Appendix S1:** Supporting Information

## Data Availability

The data that support the findings of this study are available from the corresponding author upon reasonable request.
