# Supplementary material for: Mathematical analysis of long‐distance polar auxin transport data of pin mutants questions the role of PIN1 as postulated in the chemi‐osmotic theory
Source: Physiol Plant. 2025 Mar 13;177(2):e70139. doi: 10.1111/ppl.70139 (PMC11904757; doi:10.1111/ppl.70139)
Supplement: Supplementary file 1 — Appendix S1: Supporting Information [file PPL-177-e70139-s001.pdf]

Supporting Material to:

'Multi-scale mathematical analysis of long-distance polar auxin transport data of *pin* mutants questions the role of PIN1 as postulated in the chemi-osmotic theory'

Kees J.M. Boot, Sander C. Hille, Kees R. Libbenga,
Marijke Libbenga-Nijkamp, Omid Karami, Bert van Duijn
and Remko Offringa

7th May 2024

Contents

1 The BHL-model revisited **3**
- 1.1 Underpinning of compartmental modelling 4
- 1.2 Overview of BHL-model parameters and values 10
- 1.3 Anatomical parameters 13

2 Comparison of transport velocity **15**
- 2.1 Model-based estimates for PAT velocity 15
- 2.2 Statistical tests 20
- 2.3 Clustering 21

3 Derivation of effective rate of a co-transporter **24**

4 Derivation of equations of the LPTF **29**
- 4.1 Derivation of effective transport velocity and diffusivity . . . 29
- 4.2 Parameters in BHL model expressed in terms of a cell-to-cell microscopic transport model 32
  - 4.2.1 Derivation of expressions for p and q 34
  - 4.2.2 Expressions for a and b 38
  - 4.2.3 Auxiliary parameters values in the LPTF 39

5 Determining LPTF key parameters from macroscopic fit of data 41

6 Expression levels 43
6.1 Overview of primers 43
6.2 Statistical tests 43

1 The BHL-model revisited

A mathematical model to describe the loading of IAA from the donor well, the transport inside the stem segment and the unloading of IAA onto the receiver well has been developed and experimentally validated in Boot *et al.* [1]. There it was called 'Model III'. It has been summarized in the Main Text (MT), Box 1 in particular, where we preferred to name it 'BHL-model'.

The BHL-model as exhibited in Boot *et al.* [1] will be derived again in this section – using a different approach – showing how various assumptions and anatomical structure have been incorporated into the model. This will aid later on in assessing and interpreting changes in values of parameters in the model. Moreover, the BHL-model will be extended by relating parameters to physical and physiological parameters at the microscopic scale of various parts of membranes of PAT cells and carriers contained in them. This extension is called the Long-distance Polar axin Transport Framework (LPTF). It will be discussed in depth in Section 4. The way in which microscopic key parameters can be related to those obtained in a fit of the BHL-model is discussed in Section 5. The established theoretiocal relationships are essential for the interpretation of the various *pin1* mutants that have been subjected to PAT and extended PAT experiments.

Concerning the BHL-model, we want to stress here the point that the detailed anatomy of the cross section of an inflorescence stem segment is taken into account in the BHL-model *implicitly*, i.e. not by employing a three-dimensional spatial model. The influence of anatomical differences is reflected in anatomical parameters that capture the key characteristics of the anatomy, as they appear in the BHL-model derivation below.

Let us elaborate further on this point. Although we start from a three-dimensional spatial model for an inflorescence stem segment (ISS) with local concentration of indole-3-acetic acid (IAA, auxin) at every point in this segment at any time, we reduce this three-dimensional model to a model that describes the average concentration of IAA in a cross-section of the stem segment at position x from the end of the segment that is positioned in the donor well (at $x = 0$). More precisely, in Boot *et al.* [1] we distinguished between four parts of the segment because of their different role and location in long-distance PAT:

(1) The total of PAT cell files, in which auxin is transported polarly, located within the vascular bundles (the **'U-compartment'**),

(2) The total environment of these cell files within the vascular bundles, with transport by diffusion, but no polar transport (the **'W-compartment'**),

(3) Immobilized IAA (the **'Z-compartment'**), assumed in the model to be located within the PAT cell files, and finally,

(4) The remaining totality of tissue of the stem segment outside the vascular bundles, that accumulates auxin through the contact of this tissue with the medium in the donor well (the **'Y-compartment'**).

The Y-compartment may be considered an 'experimental artefact', because the experimental set-up places one end of an inflorescence stem segment in full contact with tritium-labelled IAA in the donor well, over the full cross section of the stem. Fluorescence microscopy with DR5 markers for the presence of auxin did not show substantial amounts of auxin outside the vascular bundles in the main part of the stem segment however [1]. Therefore, accumulation of IAA in the Y-compartment seems to occur due to this direct contact of the tissue with the labelled IAA in the donor well.

The average concentrations within these compartments in a cross section at position x and time t are denoted by $[U](x,t)$, $[W](x,t)$, $[Z](x,t)$ and $[Y](x,t)$. The cross-sectional areas of these four compartment are represented by anatomical parameters S_u, S_w, S_z and S_y. By assumption, $S_z = S_u$, so S_z will not be used in the equations. It is by these parameters and the additional anatomical parameter S^0, which represents the total length of the interface between the U-and W-compartment in a cross section, that the anatomy of the stem segment is taken into account in the BHL-model. See Section 1.3 for further discussion and typical values for the anatomical parameters. We assume these areas and lengths to be constant with position x of the cross section (in first approximation).

1.1 Underpinning of compartmental modelling

The above described approach to modelling by means of compartmentation is a specific instance of a general 'top-down' approach to modelling that we shall now briefly present. By doing so, it can be assessed what (mathematical) conditions ensure that a reasonably well approximation results. To that end, we consider our stem segment as a fixed set Ω of three-dimensional space. We pick, as in the model, an orthogonal coordinate system (x, y, z) such that the x-axis coincides with the central axis of the stem segment, with $x = 0$ located at the end that is in the donor well and $x = L$ at the end

in the receiver well (Figure 1). Thus, at every $0 \le x \le L$ one has a cross section $\Omega(x) := \{(y,z) \in \mathbb{R}^2 : (x,y,z) \in \Omega\}$ of the stem segment.

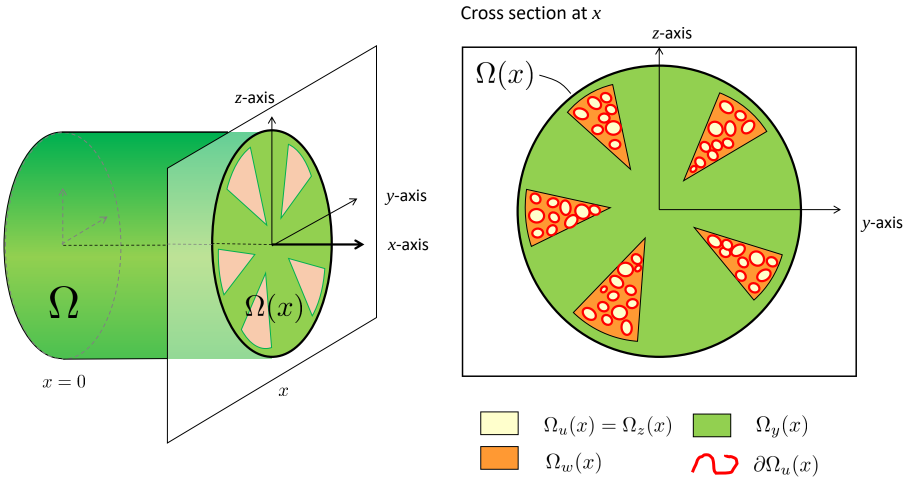

Figure 1: **Schematic overview of the various spatial domains** *in the master transport model. x-axis has been aligned with the centre line of the inflorescence stem segment, such that $x = 0$ corresponds with the end that resides in the donor well. Ω represents the entire stem segment in three dimensions. Ω_u, Ω_w, Ω_y and Ω_z are the spatial regions within Ω of the U-compartment (PAT cell files), W-compartment (remainder of vascular bundles), and Y- and Z-compartents. $\Omega(x)$ and $\Omega_u(x)$, etc., represent the cross-section of these sets perpendicular to the x-axis, at position x. The total interface between the U- and W-compartments in this cross section, $\partial\Omega_u(x)$, is indicated in red.*

Its area, $S = |\Omega(x)|$, is assumed fixed. Within this cross section, we identify the part $\Omega_{vb}(x)$ that constitutes the vascular bundles, of area $S_{vb} = |\Omega_{vb}(x)$, also assumed constant. The Y-compartment at x is then the complement of $\Omega_{vb}(x)$ in $\Omega(x)$. Similarly, one identifies $\Omega_u(x)$ and $\Omega_w(x)$ as complementing subsets of $\Omega_{vb}(x)$ that overlap only by the interface between the U-and W-compartment, $\partial\Omega_u(x)$. S^0 is the length of this interface in the cross-section. Thus:

$$\Omega_{vb}(x) = \Omega_u(x) \cup \Omega_w(x), \qquad \partial\Omega_u(x) = \Omega_u(x) \cap \Omega_w(x),$$
$$S_u = |\Omega_u(x)|,\ S_w = |\Omega_w(x)|,\ S_u + S_w = S_{vb}, \quad S^0 = |\partial\Omega_u(x)|.$$

The distribution of 'free' IAA throughout the stem segment at time t is

given by the concentration function $C(x,y,z,t)$, with $(x,y,z)\in\Omega$. The distribution of immobilized IAA is given by $C_{im}(x,y,z,t)$. Then, the variables in the BHL-model are precisely

$$
\begin{aligned}
[U](x,t) &:= \frac{1}{S_u}\int_{\Omega_u(x)} C(x,y,z,t)\,dydz,\\
[W](x,t) &:= \frac{1}{S_w}\int_{\Omega_w(x)} C(x,y,z,t)\,dydz,\\
[Y](x,t) &:= \frac{1}{S_y}\int_{\Omega_y(x)} C(x,y,z,t)\,dydz,\\
[Z](x,t) &:= \frac{1}{S_u}\int_{\Omega_u(x)} C_{im}(x,y,z,t)\,dydz.
\end{aligned}
$$

That is, they represent the average concentration of IAA in the four compartments. These give a good representation of C (and C_{im}) if fluctuations of this concentration within each of their spatial domains are small compared to the average. It is very hard – if not impossible – with the current state of techniques to measure concentration differences within the stem segment. Therefore, we simply start from embracing this assumption, resulting in a model with the least complexity. If experimental evidence suggests that this assumption is violated, then the model can be adapted in a future modification, with increased complexity.

Write $\mathbf{x}=(x,y,z)$. Seeing that transport of IAA takes place within the stem segment, one can formulate very general 'master equations' for the change in time of $C(\mathbf{x},t)$ and $C_{im}(\mathbf{x},t)$ by (inhomogeneous) diffusion and advection:

$$
\partial_t C = \nabla\cdot\big(D(\mathbf{x})\nabla C\big) - \nabla\cdot\big(V(\mathbf{x})C\big) \;-\; k_{im}^{+}(\mathbf{x})C \;+\; k_{im}^{-}(\mathbf{x})C_{\mathrm{im}}, \quad (1)
$$

$$
\partial_t C_{im} \;=\; k_{im}^{+}(\mathbf{x})C \;-\; k_{im}^{-}(\mathbf{x})C_{im}, \quad (2)
$$

where $k_{im}^{+}(\mathbf{x})$ and $k_{im}^{-}(\mathbf{x})$ are (local) immobilisation and remobilisation rates of auxin at position $\mathbf{x}$, $D(\mathbf{x})$ is the local diffusivity and $V(\mathbf{x})$ the local advective velocity field. It should be clearly realized that equations (1)–(2) are still highly generic. It is by making further assumptions on these local rates, diffusivity and velocity field that our model reduces to the BHL-model.

For example, we assume that $V(\mathbf{x})$ vanishes, except in the U-compartment

$$
\Omega_u = \bigcup_{0\le x\le L}\{x\}\times\Omega_u(x).
$$

Moreover, assuming that $D(\mathbf{x})$ is essentially constant within Ω_u, equal to D_u, with small fluctations that will be neglected, and that $k_{im}^{+}(\mathbf{x}) = \kappa_1$ and $k_{im}^{-}(\mathbf{x}) = \kappa_2$ can also be considered constant for similar reasons on Ω_u and zero elsewhere, we arrive at

$$\partial_t C = D_u \Delta C - \nabla \cdot \big(V(\mathbf{x})C\big) - \kappa_1 C + \kappa_2 C_{im} \qquad \text{on } \Omega_u. \tag{3}$$

Similarly, one obtains

$$\partial_t C = D_w \Delta C \qquad \text{on } \Omega_w, \tag{4}$$

$$\partial_t C = D_y \Delta C \qquad \text{on } \Omega_y, \tag{5}$$

with Ω_w and Ω_y defined analogously to Ω_u.

Subsequent averaging of C over the domains $\Omega_\bullet(x)$, ($\bullet = u, w, y$), yields equations for $[U]$, $[W]$ and $[Y]$, provided the regions $\Omega_\bullet(x)$ do not change too much with x. For example, then – using the Divergence Theorem from vector calculus:

$$\begin{aligned}
\partial_t [W](x,t) &= \frac{1}{S_w} \int_{\Omega_w(x)} \partial_t C(x,y,z,t)\, dydz \\
&= \frac{1}{S_w} \int_{\Omega_w(x)} D_w \Delta C(x,y,z,t)\, dydz \\
&\approx D_w \partial_x^2 \left(\frac{1}{S_w} \int_{\Omega_w(x)} C(x,y,z,t)\, dydz \right) \\
&\quad + \frac{D_w}{S_w} \int_{\Omega_w(x)} \nabla_{y,z} \cdot \nabla_{y,z} C(x,y,z,t)\, dydz \\
&= D_w \partial_x^2 [W](x,t) \;+\; \frac{D_w}{S_w} \int_{\partial\Omega_u(x)} \nabla_{y,z} C(x,\eta,t) \cdot \mathbf{n}_x(\eta)\, d\sigma(\eta),
\end{aligned}$$

in which $\mathbf{n}_x(\eta)$ is the normal vector to the interface $\partial\Omega_u(x)$, pointing out of $\Omega_w(x)$, at the point η of this interface. $d\sigma(\eta)$ denotes integration over this interface. The Fickian diffusive flux density at this interface (in the cross section $\Omega(x)$) at the inside of the W-compartment in the direction of the U-compartment is precisely

$$\big(-D_w \nabla_{y,z} C(x,y,z,t)\big) \cdot \mathbf{n}_x(\eta), \qquad \eta = (y,z). \tag{6}$$

The flux density of IAA through the interface from U- to W-compartment is modelled as being governed by linear dynamics of the form

$$J = k^{+}[U] - k^{-}[W],$$

with $[U]$ and $[W]$ the concentration in the two compartments at the interface. By assumption of (approximate) homogeneity within the compartments, one has $[U] \approx [U](\mathbf{x}, t)$ and $[W] = [W](\mathbf{x}, t)$. Hence, we obtain the condition

$$\big(-D_w \nabla_{y,z} C(x, y, z, t)\big) \cdot \mathbf{n}_x(\eta) = k^-[W](x, t) - k^+[U](x, t). \tag{7}$$

Finally, we arrive at an (approximate) equation for $[W](x, t)$:

$$\partial_t [W](x, t) = D_w \partial_x^2 [W](x, t) - k^- \frac{S^0}{S_w} [W](x, t) + k^+ \frac{S^0}{S_w} [U](x, t). \tag{8}$$

For $[U]$ we can make a similar derivation. Here, we make the assumption that the velocity field is to first approximation entirely in the direction of increasing x, i.e. $V(\mathbf{x}) = V\mathbf{e}_x$, with $\mathbf{e}_x$ the standard basis vector in the direction of increasing x. We then arrive – after some computations – at the equation

$$\partial_t [U] = D_u \partial_x^2 [U] - V \partial_x [U] - k^+ \frac{S^0}{S_u} [U] + k^- \frac{S^0}{S_u} [W] - \kappa_1 [U] + \kappa_2 [Z]. \tag{9}$$

Essentially we used that $V(\mathbf{x})$ must be tangential to the boundary of the U, W-interface at any of its boundary points, since the flow cannot leave the U-compartment, while there is not much deviation in the velocity field $V(\mathbf{x})$ from the constant $V\mathbf{e}_x$ in the interior of the compartment.

Note the difference in the rates in the reaction terms of $[W]$ in the $[U]$ and $[W]$ equations. We define

$$a := k^+ \frac{S^0}{S_u}, \qquad b := k^- \frac{S^0}{S_w}. \tag{10}$$

The rates k^+ and k^- that characterise the rate of transport through the U, W-interface have be related to plasma membrane properties. In the LPTF this will be made specific (see Section 4 and MT, Box 2).

The BHL-model equations for $t > 0$ and $0 < x < L$ then become:

$$\begin{aligned}
\partial_t [U] &= D_u \partial_x^2 [U] - V \partial_x [U] - a[U] + b \frac{S_w}{S_u} [W] - \kappa_1 [U] + \kappa_2 [Z], && (11)\\
\partial_t [W] &= D_w \partial_x^2 [W] \qquad\qquad + a \frac{S_u}{S_w} [U] - b[W], && (12)\\
\partial_t [Y] &= D_y \partial_x^2 [Y], && (13)\\
\partial_t [Z] &= \qquad\qquad\qquad\qquad\qquad \kappa_1 [U] - \kappa_2 [Z]. && (14)
\end{aligned}$$

These need to be complemented by boundary conditions and initial values. The latter are

$$[U](x,0) = 0, \quad [W](x,0) = 0, \quad [Y](x,0) = 0 \quad \text{and} \quad [Z](x,0) = 0,$$

for all $0 < x < L$, because there is no tritium-labelled IAA in the ISS at the start of the experiment. The former are formulated in terms of the net number fluxes of IAA over the boundary separating the interior of the ISS from the donor and receiver well for each of the U-, W- and Y-compartment. For the transport from donor well into these compartments we define the fluxes at any time $t \geq 0$ by

$$J_{du} = P^{+}_{du} S_u C_d(t) - P^{-}_{du} S_u [U](0,t), \tag{15}$$

$$J_{dw} = P^{+}_{dw} S_w C_d(t) - P^{-}_{dw} S_w [W](0,t), \tag{16}$$

$$J_{dy} = P^{+}_{dy} S_y C_d(t) - P^{-}_{du} S_y [Y](0,t), \tag{17}$$

where $S_y := S - S_{vb}$. The $P^{\pm}_{du}$, $P^{\pm}_{dw}$ and $P^{\pm}_{dy}$ are effective permeability constants for these boundary layers. (Superscript '+' refers to uptake into the ISS, while '−' indicates release from the ISS). C_d is the concentration of IAA in the donor well. It changes due to the exchange with the ISS according to the ordinary differential equation (ODE):

$$V_d \frac{dC_d}{dt} = -\big(J_{du} + J_{dw} + J_{dy}\big), \qquad C_d(0) = C_{d,0}. \tag{18}$$

in which V_d is the volume of the donor well. The boundary conditions at $x = 0$ for equations (11)-(13) are then the following – stating that the amount of IAA that passes from donor well into the respective compartment is matching the flux in the interior at the boundary, as prescribed in the equation:

$$-D_u \partial_x [U](0,t) + V[U](0,t) = J_{du}/S_u, \tag{19}$$

$$-D_w \partial_x [W](0,t) = J_{dw}/S_w, \tag{20}$$

$$-D_y \partial_x [Y](0,t) = J_{dy}/S_y. \tag{21}$$

Note that the number fluxes J_{du}, J_{dw} and J_{dy} that appear on the right hand side in (19)-(21), are divided by the respective cross-sectional areas of the compartments, because the indicated fluxes defined by the differential equations are flux *densities*. Consequently, these anatomical parameters do not play a role in the simplified mathematical expressions of the boundary conditions at $x = 0$, as they cancel out in the expressions. They matter for the rate of change of C_d defined by (18).

The number fluxes from each compartment in the ISS into the receiver well are given by

$$J_{ru} = P_{ru}^{-} S_u [U](L, t), \tag{22}$$
$$J_{rw} = P_{rw}^{-} S_w [W](L, t), \tag{23}$$
$$J_{ry} = P_{ry}^{-} S_y [Y](L, t). \tag{24}$$

In these expressions we ignore uptake of IAA from the receiver well. This is done, because in the experimental protocol the receiver well is regularly emptied for measurement of its IAA contents. It is then replaced by fresh medium, without IAA. The uptake of IAA is hence assumed to be negligible. The boundary conditions at $x = L$ then become

$$-D_u \partial_x [U](L, t) + V[U](L, t) = J_{ru}/S_u, \tag{25}$$
$$-D_w \partial_x [W](L, t) = J_{rw}/S_w, \tag{26}$$
$$-D_y \partial_x [Y](L, t) = J_{ry}/S_y. \tag{27}$$

The (cumulative) total amount of IAA that has been transported into the receiver well up to time t, N_r, i.e. the sum of all sampled amounts up that time, then satisfies the ODE:

$$\frac{dN_r}{dt} = J_{ru} + J_{rw} + J_{ry}. \tag{28}$$

To conclude, we like to point out, that the benefit of using a master equation approach as exhibited above is, that it makes explicit what are consequences of particular modelling assumptions like for example the assumed homogeneity of diffusivity throughout U- and W-compartments. If assumptions turn out to be violated, this approach however provides indications what corrections could be made, by making close inspection of the original expressions and seeing how they can be better approximated.

1.2 Overview of BHL-model parameters and values

In Table 1 we provide an overview of all the parameters in the BHL-model with a brief defining description. Some have a specific value indicated. These are parameters that have been fixed to that value in all simulations, including those in the data fitting procedures. These values are the same as those taken in Boot *et al.* [1] Only those parameters without a prescribed value in Table 1 are varied when fitting. For the reader's convenience, we included already the additional auxiliary parameters that occur in the LPTF that

will be defined later. These are mentioned as '*Mesoscopic parameters*' and '*Microscopic parameters*'.

Key parameters of interest in all of this study are the 'free parameters' V, a and b. The auxin transport velocity V is the attribute that is compared in the statistical analysis of wild type and mutant plants in Section 2.1 of MT (and detailed in Section 2 below). Hence, when fitting the auxin transport data of an individual ISS in a specific batch, these key parameters and – additionally – D_u, κ_1, P^+_{du}, P^+_{dw} and P^-_{ru} have been varied.

The anatomical parameters S and S_{vb} have been determined for each ISS separately based upon measurements of the stem segment. An example of typical values for a batch of ISS from a wild type plant and those of a *pin1* mutant plant are given in Section 1.3. The fraction of the cross-sectional area of the vascular bundles that is part of the U-compartment, described by the parameter $\alpha = S_u/S_{vb}$, is kept fixed to $\alpha = 0.32$ in all simulations.

In a fitting procedure, the start values values for a and b were taken as $a = 2 \times 10^{-4}$ s^{-1} and $b = 7 \times 10^{-4}$ s^{-1}. These are viewed as reference wild type values.

In the BHL-model we used for the diffusion constant D_u a value which was slightly lower than the measured diffusivity of IAA in agar: $D = 7 \times 10^{-10}$ ms^{-1} (see Boot *et al.* [1]). Since we found that the parameters D_u and P^-_{ru} can compensate each other's effect to some extent (Boot *et al.*) [1], page 662) we fixed D_u to 2×10^{-10} ms^{-1} in the simulations.

Parameter	Unit	Value	Description
Macroscopic parameters (in BHL model)			
D_u	m^2s^{-1}		Effective diffusivity of IAA in the PAT cell files (*U*-compartment)
D_w	m^2s^{-1}	$7x10^{-11}$	Effective diffusivity of IAA in vascular bundle, outside PAT cell files (*W*-compartment)
D_y	m^2s^{-1}	$2x10^{-11}$	Effective diffusivity of IAA outside the vascular bundles (*Y*-compartment)
V	ms^{-1}		Effective auxin transport velocity in the PAT cell files (*U*-compartment)
a	s^{-1}		Rate of radial exchange of IAA from *U*- to *W*-compartment
b	s^{-1}		Rate of radial exchange of IAA from *W*- to *U*-compartment
κ_1	s^{-1}		Rate of immobilization of IAA within the *U*-compartment
κ_2	s^{-1}	$1x10^{-6}$	Rate of remobilization of IAA within the *U*-compartment
P_{du}^+	ms^{-1}		Effective permeability for IAA transport at the end of the stem segment located in the donor well, for the flux from donor well into *U*-compartment (*influx*)
P_{dw}^+	ms^{-1}		Effective permeability for IAA flux at the donor end for flux from donor well into *W*-compartment (*influx*)
P_{dy}^+	ms^{-1}		Effective permeability for IAA flux at the donor end for flux from donor well into *Y*-compartment (*influx*)
$P_{du}^-, P_{dw}^-, P_{dy}^-$	ms^{-1}	$1.5x10^{-8}$	Effective permeability for IAA transport at the donor end towards the donor well from *U*-, *W*- and *Y*-compartment, respectively (*efflux*)
P_{ru}^-	ms^{-1}		Effective permeability for IAA transport at the end of the stem segment located in the receiver well, from the *U*-compartment towards the receiver well (*efflux*)
P_{rw}^-, P_{ry}^-	ms^{-1}	$1.5x10^{-8}$	Effective permeability for IAA flux at the receiver end from the *W*- and *Y*-compartment towards the receiver well (*efflux*)
S	m^2	*measured*	Cross-sectional area of stem segment
S_{vb}	m^2	*measured*	Total cross-sectional area of vascular bundles in stem segment
α		32%	Fraction of area of vascular bundles that constitutes the PAT cell files
S_u	m^2	αS_u	Total cross-sectional area of PAT cell files within vascular bundles
S_w	m^2	$S_{\mathrm{vb}} - S_u$	Total remaining cross-sectional area in vascular bundles, outside PAT cell files
S_y	m^2	$S - S_{\mathrm{vb}}$	Total remaining cross-sectional area in stem segment, outside vascular bundles
S^0	m	$2\sqrt{\pi S_u}$	Total length of interface between *U*- and *W*- compartment in a cross section
L	mm	16.0	Length of a sample stem segment
C_{d0}	M	*measured*	Concentration of tritium-labelled IAA in donor well at start of experiment
V_d	m^3	$1x10^{-6}$	Volume of donor well
Mesoscopic parameters (in MK-model)			
D	m^2s^{-1}	$2x10^{-10}$	Diffusivity of IAA in cytoplasm of a PAT cell
l	μm	80	Average length of a PAT cell
p	ms^{-1}		Increase in effective permeability for basal-to-apical side IAA transport for the double plasma membrane – apoplast system between two cells in a PAT cell file
q	ms^{-1}		Effective permeability for two-way diffusion-like IAA transport for the double plasma membrane – apoplast system between two cells in a PAT cell file
Microscopic parameters (in extended BHL-model)			
V_{PM}	mV	-120	Plasma membrane potential of PAT cell (Raven, 1967)
z		-1	Valence of IAA anion
E	mV	zV_{PM}	
T	K	293	Absolute temperature
F	$Cmol^{-1}$	$9.648x10^{-4}$	Faraday's constant
R	$J(Kmol)^{-1}$	8.314	Universal gas constant
K_a	M	$1.58x10^{-5}$	Acidity constant of IAA (Mitchison, 1980)
h_a	M	$1x10^{-5}$	Proton (ie. H_3O^+) concentration in apoplast between two PAT cells
h_c	M	$1x10^{-7}$	Proton concentration in cytoplasm of a PAT cell
f_a		61%	Fraction of total IAA in anion form in apoplast
f_c		99%	Fraction of total IAA in anion form in cytoplasm
$P_{\mathrm{AH}}^a, P_{\mathrm{AH}}^b, P_{\mathrm{AH}}$	ms^{-1}		Apparent permeability of the PM for IAA in protonated form at apical side (a), at basal side (b) and transversal side of a PAT cell *(assumed to be equal)*
$P_{A^-}^a$	ms^{-1}	$1.5x10^{-8}$	Permeability of the plasma membrane for IAA in anion form at apical side of a PAT cell, in absence of electric field
$P_{A^-}^b$	ms^{-1}		Permeability of the plasma membrane for IAA in anion form at basal side of a PAT cell, in absence of electric field

Table 1: *Overview of BHL-model parameters and those values that are fixed throughout simulations for determining auxin transport velocity. Meso- and microscopic (auxiliary) parameters have been added, for completeness.*

1.3 Anatomical parameters

For each stem segment that was subjected to PAT assay experimentation a microscopy image of the cross section was photographed and printed on paper. Anatomical structures were identified and the corresponding parts were cut out from the print. The weight of each was determined using a micro-scale. These were compared to the total weight of the excised cross-section of the ISS in the print. The total area of S was determined by means of the weight per unit area of the print paper that was used.

Table 2 shows the result for a batch of wild type plants from which nine individual stem segments were examined. Table 3 shows similar results for a batch of *pin1* mutant plants. The difference in anatomy between wild type plants and *pin1* mutants is illustrated by two typical examples of microscopy images for these (Figure 2).

Sample	No. VBs	Weight of excised parts (g)				Area ($\times 10^{-6}$ m^2)		
		Cortex	Pith	Total VBs	Total	S	S_{vb}	S_{vb}/S (%)
1.	8	0.213	0.761	0.153	1.127	1.42	0.19	13.9
2.	8	0.129	0.655	0.156	0.94	1.18	0.20	16.6
3.	8	0.205	0.837	0.164	1.206	1.52	0.21	13.6
4.	8	0.275	0.906	0.200	1.381	1.74	0.25	14.5
5.	6	0.177	0.721	0.146	1.044	1.31	0.18	14.0
6.	6	0.218	0.752	0.138	1.108	1.39	0.17	12.5
7.	6	0.285	0.888	0.164	1.337	1.68	0.21	12.3
8.	6	0.202	0.657	0.122	0.971	1.22	0.14	11.5
9.	7	0.219	0.707	0.140	1.066	1.34	0.18	13.1
					Average:	1.42	0.19	13.5

Table 2: *Measured anatomical characteristics for samples of individual stem segments of inflorescence stems from a batch of wild type Arabidopsis thaliana plants. VB = Vascular Bundle.*

The value of α, i.e. the fraction of thr area in the cross section of the vascular bundles that constitute the PAT cell files – the so-called 'U-compartment' in the BHL-model, has been based on an analysis of a selection of microscopic images of cross sections of stem segments, together with the experience of giving a proper fit of the experimental data, as found in [1]. The precise anatomical location of the PAT cell files, as postulated in the Michison model for PAT, has not been completely identified. See [1] for a more detailed discussion of this topic.

Sample	No. VBs	Weight of excised parts (g)				Area ($\times 10^{-6}$ m^2)		
		Cortex	Pith	Total VBs	Total	S	S_{vb}	S_{vb}/S (%)
1.	6	0.184	0.478	0.188	0.850	1.07	0.24	22.1
2.	5	0.184	0.382	0.136	0.702	0.883	0.17	19.4
3.	7	0.149	0.388	0.153	0.690	0.868	0.19	22.2
4.	5	0.185	0.403	0.122	0.710	0.893	0.15	17.2
5.	7	0.125	0.424	0.198	0.747	0.940	0.25	26.5
6.	6	0.171	0.348	0.169	0.688	0.866	0.21	24.6
7.	5	0.128	0.332	0.130	0.590	0.742	0.16	22.0
8.	5	0.144	0.401	0.120	0.665	0.837	0.15	18.0
9.	5	0.165	0.376	0.142	0.683	0.859	0.18	20.8
					Average:	0.884	0.19	21.4

Table 3: *Measured anatomical characteristics for samples of individual stem segments of inflorescence stems from a batch of pin1 mutant Arabidopsis thaliana plants. VB = Vascular Bundle.*

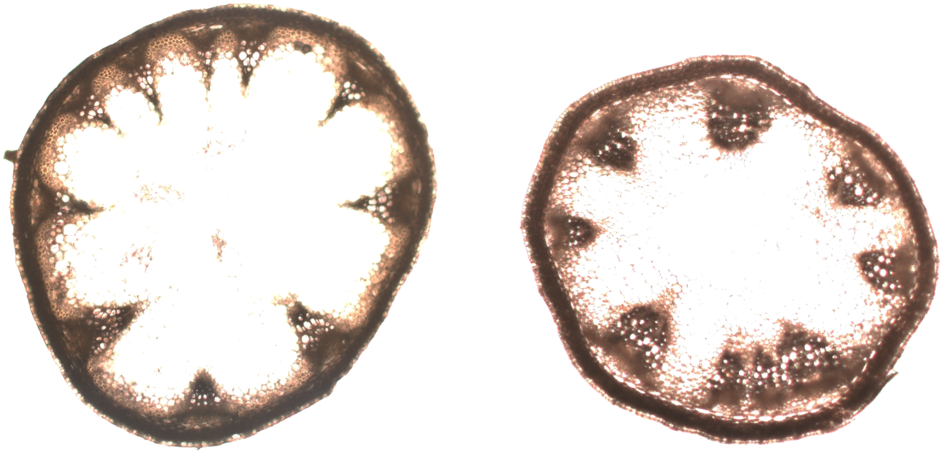

Figure 2: *Example of a microscopy image of a stem segment of an inflorescence stem of Arabidopsis thaliana, used for measuring anatomical characteristics. Left: a wild type plant; right: a pin1 mutant.*

2 Comparison of transport velocity

In one instance of a transport experiment a batch of N (at most nine) ISSs is measured simultaneously, with joint donor well, but individually – each ISS having a separate receiver well. The resulting data set for each ISS is called its *transport profile*. It consists of an *efflux profile* that presents the total amount of IAA that has been transported into the receiver well up to a given time, and a *tissue profile* that shows the accumulation in each of the 4mm sections of the ISS, at the of the experiment. In later experiments, also an *immobilisation ratio* has been determined. See MT for a description of the specific measurement procedure through a TLC analysis.

Two versions of efflux profile have been made: a standard PAT experiment and an extended PAT experiment. In the former, the transport through the ISS is measured for 5 hours, after which the the experiment is stopped and the tissue profile is determined. In the latter, the contents of the donor well has been replaced by fresh medium after 5 hours, after which the measurements continued for another 5 hours. At the end of the efflux measurements, the ISSs are cut into four 4 mm sections. For each of the sections the radioactive content is measured.

In Boot *et al.* [1], SM, we argued that the concept of auxin transport velocity can be properly quantified only within the context of a particular mathematical model (see discussion of this in [1], SM). Here we shall use the BHL-model to quantify auxin transport velocity as the value of the advective velocity V in this model after a fit of the model to the transport profile.

2.1 Model-based estimates for PAT velocity

We examined the PAT velocity of wild-type *Arabidopsis thaliana* plants and eight different mutants, focussing primarily on proteins from the PIN-family:

1.) **pML1::PIN1:GFP/*pin1***, where in *pin1* background plants the PIN1-GFP protein is expressed under the pML promotor. In these plants there is no PIN1 expression in the vacular bundles;

2.) ***pin3* mutant**;

3.) **pPIN::PIN1:GFP/*pin1***, where the PIN1-GFP protein is expressed under the PIN1 promotor in *pin1* mutant background plants, resulting in wild-type plants

4.) ***pin3/pin4/pin7* triple mutant**;

5.) ***pin1* mutant**; this mutant has an aberrant needle-like inflorescence.

6.) **pML::PIN1:GFP/*pin1*,*pin4* double mutant**;

7.) ***pgp1*/*pgp19* double mutant**; Both PGP1 and PGP19 can be putative auxin transport proteins. This mutant has strongly reduced inflorescences;

8.) ***pin5*/*pin6*/*pin8* triple mutant**.

All examined mutants have a wild-type appearance of the inflorescence, except for the *pin1* mutant (No. 5). For each class of mutant an experiment according to the standard auxin transport protocol was performed (PAT) or an extended PAT protocol (Ext. PAT) in which after 5 hours the medium in the donor well, containing radioactively labelled IAA, is replaced with medium without IAA. Accordingly, one measures the 'wash-out' of IAA from the stem segment in the remaining ca. 5 hours of the experiment. In each experiment PAT in N stem segments from inflorescences from plants from the same batch was measured individually.

The fitting procedure that resulted in the values for the PAT transport velocity was the following. We started from a standard parameter setting for wild-type plants as exhibited in [1]. Some of the parameter values were kept fixed, similarly to the fitting procedure in Boot *et al.* [1]. These have been listed in Table 1. The anatomical parameters, i.e. the cross sectional area of the stem segment S and the total area S_{vb} of the vascular bundles within this section, were measured based on microscopy images. Thus, these vary per individual stem segment (see Table 2 and Table 3 for examples).

The boundary conditions in the BHL-model represent all processes that happen at these ends of the stem segment. Anatomically, they constitute a wound layer, caused by the cutting procedure. The details of processes inside this wound layer are not our main interest. However, they must be incorporated phenomenologically in order that loading of IAA into the stem segment is represented well.

The initial conditions were set to $[U](x, 0) = 0$, $[W](x, 0) = 0$, $[Y](x, 0) = 0$ and $[Z](x, 0) = 0$ for all $0 < x < L$, since no radioactively labelled auxin is present in the system at the start of the experiment. $C_d(0) = C_{d0}$, the initial concentration of tritium-labelled IAA in the medium in the donor well. This concentration may vary between experiments. Its value has been determined for each experiment. Roughly, $C_{d0} \sim 1 \times 10^{-4}$ mol/m^{-3} $= 0.1$ μM. The total amount of accumulated IAA in the donor well satisfies $N_r(0) = 0$.

For simulating an extended PAT essay, at $t = 300$ min, C_d is changed instantaneously to 0.

The remaining parameter values were first manually tuned from the initial values to get a fit close to the data. This to prevent the computer-assisted fitting procedure, implemented in COMSOL-MATLAB, to get stuck in a local minimum of the function that measures the deviation of the simulation output from the observed data, the *cost function.*

> *Since transport profile and tissue profile are different type of characteristics of PAT, a quantifier for the* **total** *deviation of simulation from data can be constructed only from* **relative errors** *for each profile separately.*

We denote by $N_{r,i}^{\mathrm{dat}}$ the measured cumulative amounts of IAA in the receiver well at times $t = t_i$, $i = 1, \ldots m$, in the transport profile, and by N_j^{dat}, $j = 1, \ldots, 4$, the total amount of IAA in the j-th 4mm-slice of the stem segment, counting from the apical side. The time of the end of the experiment is T_{end}. We have $N_{r,m}^{\mathrm{dat}}$ amount of IAA in the receiver at this end time. The relative deviation of the efflux profile is then defined by the root total squared deviation over the data points, relative to the total amount that has been transported:

$$D_{\mathrm{effl}} := \frac{1}{N_{r,m}^{\mathrm{dat}}} \left(\sum_{i=1}^{m} \big(N_{r,i}^{\mathrm{dat}} - N_r(t_i) \big)^2 \right)^{1/2}. \tag{29}$$

Similarly, the relative deviation of the tissue profile is defined as

$$D_{\mathrm{tiss}} := \frac{\left(\sum_{j=2}^{4} \big(N_j^{\mathrm{dat}} - I_j \big)^2 \right)^{1/2}}{\sum_{j=2}^{4} N_j^{\mathrm{dat}}}, \tag{30}$$

where I_j is the total IAA content of the j-th 4mm-section in the simulation. That is,

$$I_j := \int_{\frac{1}{4}(j-1)L}^{\frac{1}{4}jL} \big([U] + [W] + [Y] + [Z] \big)(x, T_{\mathrm{end}})\, dx, \qquad j = 1, 2, 3, 4. \tag{31}$$

Note that the first 4mm section is excluded from the relative deviation of the tissue profile in (30). This has been done deliberately, since the amount of IAA in the the first 4mm section turned out to be well-adjustable by means of tuning the parameters D_y and P_{dy}^{+}, independently from the others. Therefore, deviation of the first segment does not provide a good indication of the quality of fit provided by the parameters of interest, V in particular.

Table 4 provides an overview of the experiments that have been performed, providing a statistical summary of the V-values found by fitting the model to the data for each individual stem segment. Unit for the average and standard deviation is 10^{-6} m/s.

In the part of the Main Text in which we investigate the *pin1* and *aux1/lax1-3* mutant plants and the pML1::PIN1:GFP/*pin1* plants with wild type, the experimental procedure has been expanded by including also the measurement of the *immobilisation ratio* by means of TLC analysis (see Main Text, M&M). If we denote the thus measured immobilisation ratio in the middle-two 4mm sections of the stem segment by $\rho_{\text{imm}}^{\text{dat}}$ and the simulated value by ρ_{imm}, then

$$\rho_{\text{imm}} := \frac{1}{I_2 + I_3} \int_{\frac{1}{4}L}^{\frac{3}{4}L} [Z](x, T_{\text{end}})\, dx \tag{32}$$

and the relative deviation of measured and simuated immobilisation ratio is then computed as

$$D_{\text{imm}} := \frac{\left|\rho_{\text{imm}}^{\text{dat}} - \rho_{\text{imm}}\right|}{\rho_{\text{imm}}^{\text{dat}}}. \tag{33}$$

The total deviation, or cost function, that is minimized in the fitting procedure is then the sum of the relative deviations of transport and tissue profile, with that of the immobilisation ratio added when immobilisation data is available. Fitting is considered good when the total deviation droped below 6%. The procedure is then stopped.

Batch No.	Exp. type	N	Average	St. dev.	p-value	p-value (log)
**Wild type:**						
1.	Exp. PAT	9	4.08	0.761	0.184	0.102
2.	PAT	7	3.47	0.541	0.513	0.461
3.	PAT	9	3.38	0.543	0.431	0.497
4.	Exp. PAT	8	3.09	0.610	0.677	0.688
**pML1::PIN1:GFP/*pin1* mutant:**						
5.	PAT	7	1.84	0.360	0.772	0.636
6.	PAT	6	1.92	0.319	0.667	0.6592
7.	Ext. PAT	8	2.86	0.826	0.309	0.473
8.	Ext. PAT	8	2.89	0.566	0.860	0.933
***pin3* mutant:**						
9.	PAT	9	3.63	0.714	0.164	0.265
10.	PAT	9	3.94	0.700	0.151	0.175
11.	Ext. PAT	9	3.36	0.566	0.383	0.354
**pPIN::PIN1:GFP/*pin1* mutant:**						
12.	PAT	8	4.49	0.969	0.203	0.195
***pin3/pin4/pin7* triple mutant:**						
13.	PAT	8	3.40	0.267	0.185	0.173
14.	PAT	8	4.88	1.025	0.309	0.321
***pin1* mutant:**						
15.	PAT	9	2.56	0.260	0.110	0.082
**pML::PIN1:GFP/*pin1,pin4* double mutant:**						
16.	PAT	8	2.51	0.503	0.595	0.719
***pgp1/pgp19* double mutant:**						
17.	PAT	9	2.49	0.289	0.229	0.258
18.	Ext. PAT	9	2.66	0.410	0.063	0.120
***pin5/pin6/pin8* triple mutant:**						
19.	Ext. PAT	9	4.63	0.287	0.0006*	0.0005*

Table 4: *Statistical summary of the V-values for the batches, organized by mutant type. Average V and standard deviation (st. dev.) are stated in units of 10^{-6} m/s. The indicated p-value is the result of the Shapiro-Wilk test for normality for the velocity values for the batch. The last column ('p-value (log)') gives this value for the log-transformed V-values. 'PAT' indicates the use of the standard polar auxin transport protocol in the experiments, while 'Exp. PAT' indicates the use of the extended PAT protocol. N is the number of stem segments (samples) in the batch.*

2.2 Statistical tests

The statistical test reported on in this section have been performed in R, version 4.04 - 64 bit, combined with R Studio v1.41106. A standard confidence level of 5% was taken in all tests.

Our objective is to test the hypothesis that the mean auxin transport velocity V in all experimental batches is the same among the batches. To that end we performed a one-way ANOVA (see eg. [10], Chapter 12). This test assumes the following:

(A1) All observations are independent;

(A2) There are no significant outliers in any group;

(A3) The values are normally distributed within each group;

(A4) The variances in every group are equal.

Independence of observations (Assumption (A1)) is acceptable, since every batch has been treated separately from others, in time or space – using different petri dishes. Though within a batch, the stem segments in a petri dish have a common donor well, they all have an individual receiver well. The volume of the donor well is very large compared to the total amount of auxin transported. Therefore, direct influencing of stem segments is implausible or negligible.

Concerning Assumption (A3) we performed the Shapiro-Wilk test for normality on each of the batches. The resulting p-values are reported in the column 'p-value' of Table 4. The hypothesis of normality of the data had to be rejected only for Batch 19. Ignoring this for the moment, we performed Levene's Median Test (following Hines & Hines [4], see also [3]) to test for equal variance among the 19 batches (Assumption (A4)). This hypthesis had to be rejected ($F(18, 138) = 2.29,\ p = 0.0037$).

Considering the results of these tests and realising that the velocities, as positive quantities, may be considered log-normally distributed rather than normal, we log-transformed the V-data. On each batch we performed again the Shapiro-Wilk test. The corresponding p-values are reported in the last column of Table 4. The hypothesis that the $\ln(V)$-values are normally distributed for each batch is statistically confirmed for all batches, except – again – for Batch 19. In Figure 3 we provide a QQ-normality plot for the log-transformed V-data. By visual inspection we deem the deviation from normality acceptable for all batches.

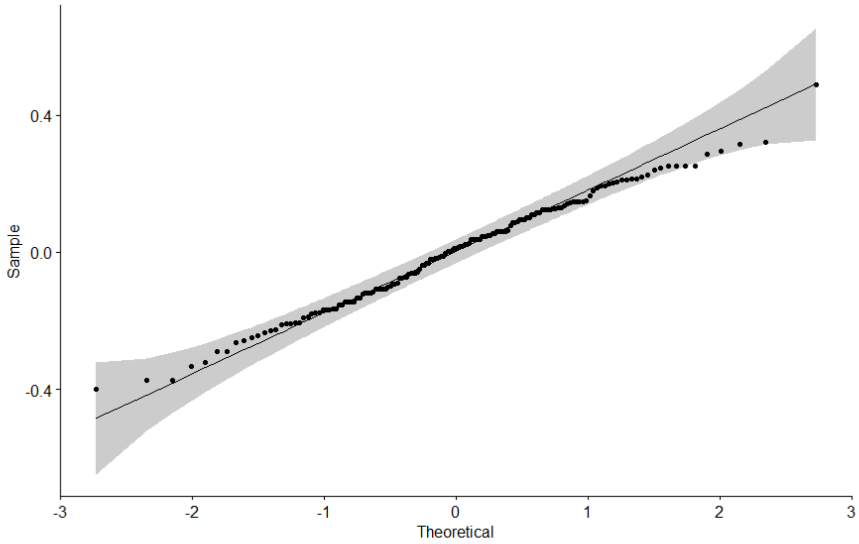

Figure 3: *QQ-normality plot for all log-transformed V-data of the 19 experimental batches.*

We then applied Levene's Median Test to the log-transformed data. This indicated that there is now no reason to reject the hypothesis of equal variance between the batches ($F(18, 138) = 1.32,\ p = 0.185$). A box plot for all batches of the $\ln(V)$-values did not show any significant outliers (Figure 4).

These results support conducting a One-way ANOVA on the log-transformed V-parameter values to evaluate if the the mean transport velocity is the same among the experimental batches, consisting of wild-type plants and mutants. Mean PAT velocity was significantly different between the batches ($F(18, 138) = 19.18,\ p = 4.12 \times 10^{-29}$, generalized eta squared $= 0.71$). A visual inspection of the box plot in Figure 4 confirms this finding.

2.3 Clustering

We further examined how the experimental batches may be clustered such that the log-transformed V-values within a cluster can be considered as having the same mean. We therefore performed a Tukey post-hoc analysis. Inspection of its output indicated four potential clusters of batches on each of which we performed separately a one-way ANOVA to test the hypothesis that within the potential cluster the batches may be considered having the same mean (log-) transport velocity. There was no reason to reject this hypothesis for each cluster. Figure 5 shows the identified clustering in colours.

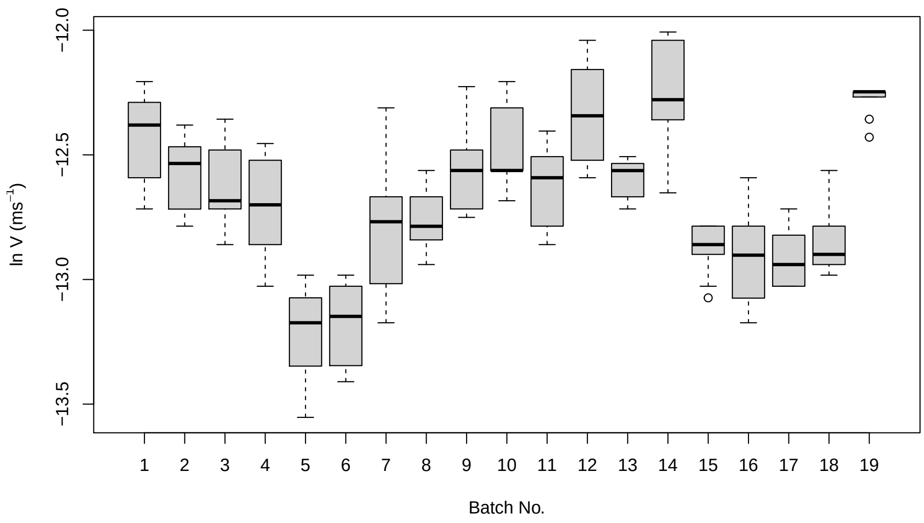

Figure 4: *Box plot of all log-transformed V-data for each of the 19 experimental batches. Batch No. refers to the batch numbers listed in Table 4. V values were expressed in units of* ms^{-1} *before natural logarithm was applied.*

The potential cluster composition and one-way ANOVA results were:

- Cluster '*High*': batches 1, 12, 14 and 19.
  ANOVA: $F(3, 30) = 1.49,\ p = 0.24,\ \text{ges} = 0.13$.
- Cluster '*Middle-top*': batches 2,3,4,9,10,11 and 13.
  ANOVA: $F(6, 52) = 1.77,\ p = 0.12,\ \text{ges} = 0.17$.
- Cluster '*Middle-low*': batches 7,8,15,16,17 and 18.
  ANOVA: $F(5, 45) = 1.06,\ p = 0.39,\ \text{ges} = 0.11$.
- Cluster '*Low*': batches 5 and 6.
  ANOVA: $F(1, 11) = 0.18,\ p = 0.68,\ \text{ges} = 0.016$.

In order to see whether any of the potential clusters could be enlarged without the need to reject the hypothesis of having equal mean (log-) transport velocity, we started with the batches in a cluster and one-by-one added one of the remaining batches and tested the hypothesis of equal mean by performing a one-way ANOVA. Adding any of the High-cluster batches (red) to the Middle-top cluster (blue) resulted in a rejection of the hypothesis

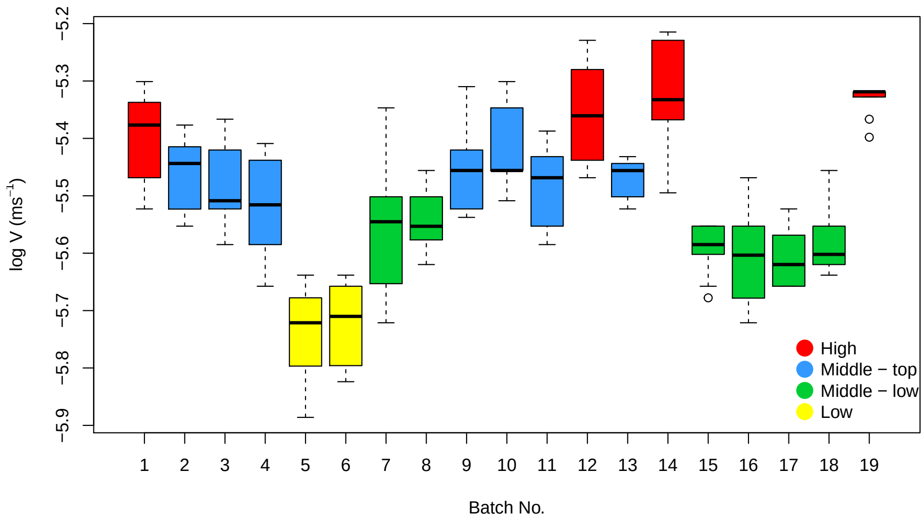

Figure 5: *Box plot of all log-transformed V-data for each of the 19 experimental batches, coloured to indicate the identified clustering. V values were expressed in* ms^{-1} *before 10-base logarithm was applied. Base-10 logarithm has been used here for ease of interpretation by the reader.*

($p = 0.032$, $p = 0.0003$, $p = 0.00016$, $p = 6.7 \times 10^{-5}$ and $p = 3.8 \times 10^{-5}$ for batch no. 1, 12, 14, 15 and 19 respectively). Similarly, adding any of the batches of the Middle-low cluster (green) to the Middle-top cluster resulted in a group for which the hypothesis of equal mean velocity had to be rejected too ($p = 0.021$, $p = 0.014$, $p = 6.7 \times 10^{-5}$, $p = 0.00014$, $p = 1.9 \times 10^{-5}$ and $p = 00050$ for the batches no. 7, 8, 15, 16, 17 and 18 respectively). In a similar fashion, adding any Low-cluster batch to the Middle-low cluster yields a subgroup with significantly unequal mean velocity ($p = 0.00022$ and $p = 0.0020$ for adding batch no. 5 and 6 respectively).

3 Derivation of effective rate of a co-transporter

An expression for the rate of transport of a transmembrane transporter as a function of concentrations of the compounds involved on either side of the membrane may be computed using the King-Altman diagrammatic method, see [6, 2]. It models the transporter (or enzyme) in a finite number of states, numbered by $i = 1, \ldots, n$, and assigns transition rates k_{ij} to the transition from state i to state j. That is, an individual transporter is modelled as a finite state Markov process in continuous time transition probabilities k_{ij} per unit time from state i to j. Thus, the waiting time T_{ij} for the transition from state i to j has (cumulative) probability distribution

$$\mathrm{Prob}\big(T_{ij} \leq t\big) = 1 - e^{-k_{ij}t}, \qquad t \geq 0.$$

Figure 6, Panel A, shows the transition diagram of a six-state ($n = 6$) transmembrane co-transporter that binds its cargo (C) and a compound (S) that is transported together with the cargo on one side and then, through conformational changes driven by thermodynamic fluctuations, releases them on the other side of the membrane. It is the simplest model of such kind. See [11] for more complicated state diagrams for co-transporters and a discussion that those can essentially be reduced to the scheme shown in Panel A. The precise order of binding of cargo C and co-transport S is not relevant to the form of the effective transport rate that will be computed, as will be explained below.

The effective transport rate is computed under the assumption that the fraction $x_i(t)$ of all N transporters in a given state i at time t on the membrane enclosing the 'inside' compartment of volume V has settled to a steady state distribution. This steady state fraction is denoted x_i^*. The total change in concentration of cargo C in the inside compartment, caused by all N transporters, i.e. the overall net transport rate, is then given by

$$R^* = \frac{N}{V}\big[k_{56}x_5^* - k_{65}x_6^*\big]. \tag{34}$$

Here, $N \cdot k_{56}x_5^*$ is the total average number of transitions from state 5 to 6 per unit time in the whole population of transporters, resulting into an influx of C into the inside compartment, while $N \cdot k_{65}x_6^*$ represents the total average number of transitions from state 6 to 5 per unit time, which each picks up 1 cargo molecule from the inside compartment. The transition rate k_{65} depends on the concentration of cargo on the inside, like other transition

rates that correspond to binding of compound in the diagram. We use mass-action kinetics for the description of these transition rates:

$$k_{12} = k^*_{12}[C]_{\text{out}}, \quad k_{23} = k^*_{23}[S]_{\text{out}}, \quad k_{65} = k^*_{65}[C]_{\text{in}}, \quad k_{54} = k^*_{54}[S]_{\text{in}}.$$

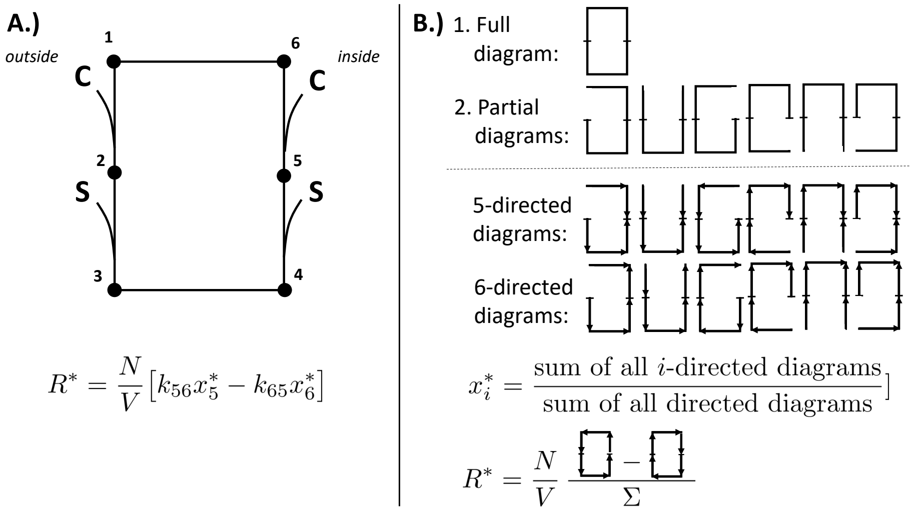

Figure 6: **Panel A**. *Six-state diagram for a transmembrane transporter hat carries its main cargo C and the compound S that is transported in the same direction together with C (i.e. the symported compound) from 'outside' to 'inside'. All state transitions are assumed reversible.* k_{ij} *is the rate of transition from state* i *to* j*.* x^*_i *denotes the fraction of transporters in state* i *at steady state.* R^* *is the net steady state rate of transport of C to the inside (change of concentration), when* N *transporters are present on the membrane and the inner compartment has volume* V*.* **Panel B.** *Computation of* R^* *by means of the King-Altman diagrammatic method.* Σ *denotes the sum of all directed diagrams. A directed diagram represent the product of rate constants* k_{ij} *for those pairs* (i, j) *for which there is an arrow from state* i *to* j *in the diagram.*

The King-Altman diagramatic method amounts to computing the steady state fractions of transporters in state i, x^*_i, or – put differently – the steady state probability that a single transporter will be in state i according to the Markov model, by solving the corresponding linear steady state equation essentially through Cramer's Rule, but employing a diagrammatic calculus. Our exposition is based on [6, 2]. The calculus works as follows.

First one determines all *partial diagrams* from the full state diagram, by removing a minimal number of transitions (edges) such that the resulting

diagram no longer contains cycles. In this case, only one transition needs to be removed, what can be done in six different ways (see Figure 6, Panel B). Then one determines for each $i = 1, \ldots, n$ all i-directed diagrams: one gives each transition a direction, indicated by an arrow, such that the resulting path flows towards state i. We have shown all 5- and 6-directed diagrams.

> *In the diagrammatic calculus, a directed diagram corresponds to a product of precisely those transition rates k_{ij} for which there is an arrow pointing from state i to state j in the diagram.*

Accordingly, one gets

$$x_i^* = \frac{\text{‘sum of all } i\text{-directed diagrams’}}{\text{‘sum of all directed diagrams’}}. \tag{35}$$

The sum of all diagrams is a complicated expression, which is commonly denoted by

$$\Sigma = \text{‘sum of all directed diagrams’},$$

which involves in its expression the concentrations of C and S on inside and outside. By inspection partial diagrams, it can be reasoned that Σ must be of the form

$$\begin{aligned}\Sigma = \hat{k}_0 + \hat{k}_1[C]_{\text{in}} + \hat{k}_2[S]_{\text{in}} + \hat{k}_3[C]_{\text{out}} + \hat{k}_4[S]_{\text{out}} \\ + \hat{k}_5[C]_{\text{in}}[S]_{\text{in}} + \hat{k}_6[C]_{\text{out}}[S]_{\text{out}} \\ + \hat{k}_7[C]_{\text{in}}[S]_{\text{in}}[S]_{\text{out}} + \hat{k}_8[C]_{\text{out}}[S]_{\text{out}}[S]_{\text{in}} \\ + \hat{k}_9[C]_{\text{in}}[S]_{\text{in}}[C]_{\text{out}} + \hat{k}_{10}[C]_{\text{out}}[S]_{\text{out}}[C]_{\text{in}} \\ + \hat{k}_{11}[C]_{\text{out}}[S]_{\text{out}}[C]_{\text{in}}[S]_{\text{in}}\end{aligned}$$

For example, the triple factor of concentrations in the term with constant $\hat{k}_7$ occurs e.g. as the 4-directed diagram in which transition 1-2 has been removed. That corresponding to k_{10} occurs e.g in the 2-directed diagram that fails transition 1-2. The last term, with constant $\hat{k}_{11}$ occurs as the the 4-directed diagram with 1-6 removed. If one whishes, one may express explicitly the parameters $\hat{k}_0, \ldots, \hat{k}_{11}$ as a function of the transition rates that are ‘concentration-free’, which we call the *elementary transition rates*, i.e. the k_{ij} or k_{ij}^* if the corresponding k_{ij} depends on the concentration of C or S.

Combining expression (34) for the net transport rate R^*, i.e. the change of concentration of the cargo C in the inside compartment, and the expression for the steady state fraction of transporters in a particular state (35) yields

through diagrammatic calculus the diagrammatic expression for R^* as shown in Figure 6, Panel B.

Next, the elementary transition rates cannot be arbitrary. They must satisfy the so-called *thermodynamic constraints*. That is, for any cycle in the full diagram, the product of all elementary transition rates corresponding to a walk along the cycle in one direction must equal the product along a walk in the opposite direction. For our diagram there is one cylce only, the full diagram, and the thermodynamic constraint amounts to the condition

$$k_{12}^* k_{23}^* k_{34} k_{45} k_{56} k_{61} \;=\; k_{16} k_{65}^* k_{54}^* k_{43} k_{32} k_{21}. \tag{36}$$

If we indicate this common value by k, then we finally obtain from the diagrammatic expression for R^* in Figure 6, Panel B:

$$R^* = \frac{N}{V} \frac{k\big([C]_{\text{out}}[S]_{\text{out}} - [C]_{\text{in}}[S]_{\text{in}}\big)}{\Sigma} \tag{37}$$

Note that a different order of binding of C and S will not change the overall shape of the steady state transport rate R^* as presented in (37).

Working with the full expression for Σ as function of the concentrations of cargo C an co-transported compound S is cumbersome. The various parameters $k_0, \ldots, k_{12}$ are very hard to determine. We shall ignore the dependence of Σ on these concentrations. Thus, we assume that concentrations remain in a range that the value of Σ may be considered aproximately constant in comparison to the other terms in the expression for R^*:

$$R^* \approx k^{\text{co}}\big([C]_{\text{out}}[S]_{\text{out}} - [C]_{\text{in}}[S]_{\text{in}}\big). \tag{38}$$

In our model for PAT we incorporate the effect of auxin anion/H^+ co-transporters, in particular those of the AUX1/LAX1-LAX3 family. Then the cargo is the auxin anion A^- and S is a proton. Moreover, auxin is a weak acid, so there is an equilibrium attained between anion and protonated form,

$$\text{A}^- \;+\; \text{H}^+ \;\rightleftarrows\; \text{AH},$$

with equilibrium constant – the *acidity constant*:

$$K_a \;=\frac{[\text{A}^-][\text{H}^+]}{[\text{AH}]} \;=\; 1.58 \times 10^{-5}\ \text{M} \tag{39}$$

(see Mitchison [8]). Therefore, in (38)

$$[\text{C}][\text{S}] \;=\; [\text{A}^-][\text{H}^+] \;=\; K_a[\text{AH}].$$

That is, the transport rate of the auxin anion through the AUX1/LAX1-LAX3 symporters may be represented in the model by

$$R^* \;=\; k^{\mathrm{co}} K_a \left([\mathrm{AH}]_{\mathrm{out}} - [\mathrm{AH}]_{\mathrm{in}}\right). \tag{40}$$

That is, *by an apparent transport of the protonated form.*

4 Derivation of equations of the LPTF

The Long-range Polar auxin Transport Framework (LPTF) connects the macroscopic BHL-model, through the intermediary mesoscopic Mitchison-Kramer (MK) model to microscopic physiological and auxiliary physical parameters that can be interpreted in view of the chemi-osmotic theory and changes due to mutations.

In the BHL-model there appear four main *effective* parameters of which the first three are key in the LPTF: the effective auxin transport velocity V, the exchange rates a and b from the U-compartment to the W-compartment and *vice versa.* The fourth parameter is the apparent diffusivity of auxin in the U-compartment, D_u. Its value is less relevant in the argumentation, because changes in its value can be compensated, to a large extent, by appropriate modifications of the parameter P_{ru}^- that controls the efflux from the stem segment, from the U-compartment, at the receiver well, as was found already in Boot *et al.* [1].

In the Main Text (see Box 2), we provided expressions for these key parameters in terms of underlying physical and physiological parameters of the cellular PAT system in terms of the unidirectional permeabilities $\beta_1, \ldots, \beta_4$. An expression for V was obtained using the intermediate mesoscopic level description provided by the MK-model of Mitchison [8] and Kramer [7] and its central parameters p and q as an intermediate step. In this section we shall provide mathematical details of the derivation of these expressions.

4.1 Derivation of effective transport velocity and diffusivity

In order to substantiate the theoretical foundation of the BHL-model for interpreting PAT data from *pin* mutants of *Arabidopsis thaliana*, we will here describe how the parameters D_u and V are linked to D_{eff} and V_{eff} in the equations described by Mitchison [8] and Kramer [7] in the MK-model. We derive afresh Mitchison's equation for the effective velocity V in terms of 'mesoscopic permeability parameters' p and q (see Main Text, Box 2) and obtain an expression for the effective diffusivity D_u as well, which differs from that determined by Kramer [7].

Fundamental building block in the MK-model is a single longitudinal cell array of polarly auxin-transporting cells (MT Box 2, Panel C). Auxin is free to travel by diffusion through the interior of these cells, with diffusion constant D, while the flux density ($\text{mol}\,\text{m}^{-2}\text{s}^{-1}$) of auxin between two adjacent

cells is postulated to have the form:

$$J = pa_1 + q(a_1 - a_2) \tag{41}$$

where a_1 and a_2 are the cytoplasmic auxin concentrations on either side of the interface between two neighbouring cells. In correspondence with the observed net flow of auxin from apical to the basal side in inflorescence stems, a_1 refers to the concentration of auxin at the basal side of a cell, and a_2 to the concentration of auxin at the apical side of the neighbouring cell. Here, p and q are (effective) permeability constants. Equation (41) reflects the assumption made in the chemi-osmotic theory of PAT that, in addition to the basic flux through the interfaces of neighbouring PAT cells governed by a permeability constant denoted q, there is an extra unidirectional polar orientated compound, governed by a permeability constant denoted by p. We assume that all cells in an array have equal (average) length l. We shall compute with a cell length $l = 80$ μm.

For ease of computation, we consider an infinitely extended cell array with coordinate x in longitudinal direction, taking all real values. This expresses mathematically, that the cell array contains so many cells, that effects on auxin transport at both ends of the array will hardly have an influence in the middle. In addition, we assume that the whole system is in a steady-state, such that there is a flux J through each cell and through each barrier between neighbouring cells. This assumption implies that inside say the i-th cell in the array, extending from position $x = x_{i-1/2}$ (the apical end point) to $x = x_{i+1/2}$ (the basal end point), the average concentration of auxin in a cross section of this cell file at x, $C_i(x)$, has a constant gradient. Therefore,

$$C_i(x) = -\frac{J}{D}(x - x_i) + U_i, \qquad x_{i-1/2} < x < x_{i+1/2}, \tag{42}$$

where U_i is the average concentration over the whole i-th cell in the array, which is obtained at the midpoint $x_i = \frac{1}{2}(x_{i-1/2} + x_{i+1/2})$. At steady state, the flux inside the i-th cell, which can be expressed as:

$$J = 2\frac{D}{l}\big(U_i - C_i(x_{i+1/2})\big) \tag{43}$$

must equal the flux over the barrier between the i-th cell and the $(i+1)$-th cell, which according to eq. (41) is given by:

$$J = (p+q)C_i(x_{i+1/2}) - qC_{i+1}(x_{i+1/2}). \tag{44}$$

The flux within the $(i+1)$-th cell must also equal J and can be written as:

$$J = 2\frac{D}{l}(C_{i+1}(x_{i+1/2})) - U_{i+1}. \tag{45}$$

By multiplying eq.(43) with $(p+q)$ and eq.(44) by $2D/l$ and eq.(45) by q and then summing the results one obtains:

$$\big(p + 2q + 2\frac{D}{l}\big)J = 2\frac{D}{l}\big[(p+q)U_i - qU_{i+1}\big]. \tag{46}$$

That is,

$$J = -D_{eff}\frac{U_{i+1} - U_i}{\ell} + V_{eff}U_i, \tag{47}$$

where

$$V_{eff} = \frac{p}{p + 2q + 2\frac{D}{\ell}} \cdot 2\frac{D}{\ell} \tag{48}$$

and

$$D_{eff} = \frac{q}{(1/2p + q + \frac{D}{\ell}} \cdot D. \tag{49}$$

We now want to relate the cell-based model to the steady-state solution of the macroscopic advection-diffusion equation for $[U](x)$ as part of the BHL-model:

$$D_u\frac{d[U]^2}{dx^2} - V\frac{d[U]}{dx} = 0,$$

where the flux is J at every point:

$$-D_u\frac{d[U]}{dx}(x) + V[U](x) = J, \qquad \text{for all } x. \tag{50}$$

The connection is made by considering spatial averages of the function $C(x)$, defined for all positions x as

$$C(x) := C_i(x), \qquad \text{when } x_{i-1/2} < x < x_{i+1/2}.$$

That is, we define the spatial average over length ℓ:

$$\bar{u}_l(x) := \frac{1}{\ell}\int_{x-\frac{1}{2}\ell}^{x+\frac{1}{2}\ell} C(\xi)d\xi. \tag{51}$$

It can be readily verified using eq.(42) that $\bar{u}_l(x)$ is the linear interpolation between the values U_i and U_{i+1} on the interval $[x_i, x_{i+1}]$. In particular, $\bar{u}_l(x_i) = U_i$ for all i. Rewriting (47) yields:

$$\frac{U_{i+1} - U_i}{\ell} = \frac{V_{eff}}{D_{eff}}U_i - \frac{J}{D_{eff}}. \tag{52}$$

Note that eq.(52) can be viewed as a discretization of the differential equation (50) for $[U](x)$ at the points x_i that are positioned at equal distance ℓ, where $V = V_{eff}$ and $D_u = D_{eff}$. Thus, $\bar{u}_l(x) \approx [U](x)$ for ℓ sufficiently small. Mitchison [8] obtained the expression for $V = V_{eff}$ as presented here in (48) as did Kramer [7], both by different arguments. Kramer also obtained an expression for D_{eff}, which differs from our eq.(49), namely:

$$D_{u,\mathrm{Kramer}} = \frac{\frac{1}{2}p + q}{\frac{1}{2}p + q + \frac{D}{l}} \cdot D. \tag{53}$$

We use $D = 2 \times 10^{-10}$ ms^{-1} and $\ell = 80$ μm in this study, see Section 1.2. Thus, according to (48),

$$V \;\leq\; V_{\max} := \frac{2D}{\ell} \;=\; 5 \times 10^{-6}\ \mathrm{ms}^{-1}. \tag{54}$$

With the introduction of the LPTF with a modified expression for the effective diffusion constant (49), we obtained a lower D_u as computed by this expression than $D_{u,Kramer}$. We realized that the double plasma membrane barrier between two neighbouring cells in a PAT cell file, forming the U-compartment, is a far greater limiting factor for the diffusion of auxin. Thus, $D_{u,\mathrm{Kramer}}$ gives a too high estimate of the effective diffusion constant, in our opinion.

4.2 Parameters in BHL model expressed in terms of a cell-to-cell microscopic transport model

We shall now consider how the parameters p and q of the MK-model can be expressed in terms of characteristics of the basal and apical cell membranes of PAT cells and the intermediate apoplast. Similarly, we shall determine expressions for a and b, thus linking the effective parameters of the BHL-model (i.e. V, D_u, a and b) to microscopic parameters that can be interpreted in terms of changes physical and physiological properties caused by mutations in the *pin* genes.

The mesoscopic parameters p and q will turn out to be expressible in terms of permeabilities $\beta_1, \ldots, \beta_4$ for the plasma membrane at apical and basal side of PAT-cells in the cell file that are determined by physical and physiological properties of these membranes, see Main Text, Box 2. For the macroscopic parameters a and b a similar approach can be taken, essentially using the parameters β_3 and β_4.

Starting point are the expressions for the β_i, $i = 1, \ldots, 4$, as exhibited and explained in the Main Text, Box 2, Panel C, which we repeat here – without derivation – for the reader's convenience.

$$\beta_1 = \bar{P}^b_{\mathrm{AH}}(1 - f_c) \; + \; P^b_{\mathrm{A}^-} f_c \frac{EF}{RT} \frac{1}{1 - e^{-EF/RT}}, \tag{55}$$

$$\beta_2 = \bar{P}^b_{\mathrm{AH}}(1 - f_a) + P^b_{\mathrm{A}^-} f_a \frac{EF}{RT} \frac{e^{-EF/RT}}{1 - e^{-EF/RT}}, \tag{56}$$

$$\beta_3 = \bar{P}^a_{\mathrm{AH}}(1 - f_a) \; + \; P^a_{\mathrm{A}^-} f_a \frac{EF}{RT} \frac{e^{-EF/RT}}{1 - e^{-EF/RT}}, \tag{57}$$

$$\beta_4 = \bar{P}^a_{\mathrm{AH}}(1 - f_c) + P^a_{\mathrm{A}^-} f_c \frac{EF}{RT} \frac{1}{1 - e^{-EF/RT}}, \tag{58}$$

Key idea in the derivation of the expressions for $\beta_1, \ldots, \beta_4$ is:

> *Separate transport of IAA over the membrane into fluxes of anion and protonated form and obtain expressions for each. The transport of the anion is sensitive – in principle – to the electric field over the membrane, due to the cross membrane potential. Electrically neutral co-transport (as in the A^-/H^+ symporters of the AUX1/LAX1-LAX3 protein family) will be effectively insensitive to this field. According to the mathematical expressions for the corresponding flux, this type of transport apparently behaves like transport of the protonated form.*

In the above expressions, E is the difference in electric potential over the plasma membrane that is felt by the IAA anion, $E = zV_{\mathrm{PM}} = -V_{\mathrm{PM}}$, where V_{PM} is the cell's membrane potential. We take $V_{\mathrm{PM}} = -120$ mV, see [9]. f_c and f_a are the fraction of total IAA in anion form in cytoplasm and apoplast, respectively, at equilibrium, which are determined by the cytoplasmic and apoplastic pH. We take the values pH = 7 and pH = 5 for these in our computations. Using the value $K_a = 1.58 \times 10^{-5}$ M (pK_a = 4.8) for the acidity constant for IAA (cf. [8]), one gets

$$f_c = \frac{K_a}{h_c + K_a} \; = \; 99\%, \qquad f_a = \frac{K_a}{h_a + K_a} \; = \; 61\%, \tag{59}$$

where h_c and h_a are the proton concentrations in cytoplasm and apoplast. F is Faraday's constant, R is the universal gas constant and T the absolute temperature (see Table 1 for values). At 20° C, the various factors occurring the expressions for the β_i then take the value:

$$\frac{EF}{RT} = 4.75, \quad e^{-EF/RT} = 8.65 \times 10^{-3}, \quad \frac{EF/RT}{1 - e^{-EF/RT}} = 4.79. \tag{60}$$

The parameters $\bar{P}^a_{\mathrm{AH}}$ and $\bar{P}^b_{\mathrm{AH}}$ are the apparent permeability constants for the plasma membrane for IAA in protonated form ('AH') at apical and basal side of the cell, respectively.

$$\bar{P}^a_{\mathrm{AH}} = P_{AH} + k^a_{co} K_a, \tag{61}$$

where k^a_{co} is a parameter of the co-transporter, see Section **??**. A similar expression holds for $\bar{P}^b_{\mathrm{AH}}$. In the LPTF (61) and the experession for $\bar{P}^b_{\mathrm{AH}}$ is used only to furnish a reasonable lower bound ($P_A H$) for $\bar{P}^a_{\mathrm{AH}}$ and $\bar{P}^b_{\mathrm{AH}}$. The latter are taken as 'free' parameters that are determined by fitting.

Similarly, $P^a_{\mathrm{A}^-}$ and $P^b_{\mathrm{A}^-}$ represent the permeability constants of these membranes for IAA in anion form ('A^-') in absense of an electric field over the membrane.

4.2.1 Derivation of expressions for p and q

We provide here some further details of the derivation of the expression for p and do the same here for the (cumbersome) expression for q, which is not shown in the Main Text.

First, we provide some more detail on the derivation of the fundamental expressions of p and q in terms of the $\beta_1, \ldots \beta_4$, stated in the Main Text, Box 2, Panel C:

$$p = \frac{\beta_1\beta_3 - \beta_2\beta_4}{\beta_2 + \beta_3}, \qquad q = \frac{\beta_2\beta_4}{\beta_2 + \beta_3}. \tag{62}$$

Let U^{n-1}_l, denote the auxin concentration at the basal side of cell $n-1$ in a file of PAT cells and let U^n_0 denote the auxin concentration at the apical side of cell n. We assume that the apoplast has thickness h and the auxin diffuses through the apoplast with diffusion constant D_{apo}. We assume that auxin is neither produced nor degraded in the apoplast. The transport of auxin through the plasma membranes at basal and apical side can be described mathematically by:

$$J^b = \beta_1 U^{n-1}_l - \beta_2 C(0), \tag{63}$$
$$J^a = \beta_3 C(h) - \beta_4 U^n_0, \tag{64}$$

where J^b is the auxin flux density through the basal plasma membrane and J^a through the apical plasma membrane. The coefficients $\beta_1, \ldots \beta_4$ are permeability constants, $C(x,t)$ represents the longitudinal auxin density

distribution inside the apoplast at position x $(0 < x < h)$ at time t. It satisfies the diffusion equation:

$$\frac{\partial C}{\partial t} = D_{\text{apo}} \frac{\partial^2 C}{\partial x^2} \qquad \text{on } (0, h) \tag{65}$$

with boundary conditions:

$$-D_{\text{apo}} \partial_x C(0) = J^b, \tag{66}$$
$$-D_{\text{apo}} \partial_x C(h) = J^a. \tag{67}$$

We assume that the diffusion in the apoplast is much faster than the transport across the plasma membranes, so that (65) is in quasi-steady state. This implies that the auxin-density profile in the apoplast is linear on $(0, h)$ and that the fluxes through the plasma membranes satisfy $J^b = J^a$ and are equal to the flux through the apoplast, J. Then we have:

$$J = \beta_1 U_l^{n-1} - \beta_2 C(0), \tag{68}$$
$$J = -D_{\text{apo}} \frac{C(h) - C(0)}{h}, \tag{69}$$
$$J = \beta_3 C(h) - \beta_4 U_0^n. \tag{70}$$

By successively eliminating the apoplastic concentrations we finally obtain:

$$J = \left[\frac{\beta_1 \beta_3}{\beta_2 + \beta_3 + \frac{h\beta_2 \beta 3}{D_{\text{apo}}}} \right] U_l^{n-1} \; - \; \left[\frac{\beta_2 \beta_4}{\beta_2 + \beta_3 + \frac{h\beta_2 \beta_3}{D_{\text{apo}}}} \right] U_0^n. \tag{71}$$

Continuing as in the Main Text, we observe that the quasi-steady state assumption is valid under the conditions

$$\frac{\beta_2 h}{D_{\text{apo}}} \ll 1 \qquad \text{and} \qquad \frac{\beta_3 h}{D_{\text{apo}}} \ll 1. \tag{72}$$

That is, the diffusive transport velocity D_{apo}/h is large compared to both permeabilities β_2 and β_3, which correspond to the fluxes of auxin from the apoplast into the cytoplasm. Taking $D_{\text{apo}} = 2 \times 10^{-10}\text{m}^2\text{s}{-1}$ and $h = 2\ \mu\text{m}$, we get

$$\frac{D_{\text{apo}}}{h} \approx \frac{2 \times 10^{-10}\text{m}^2\text{s}^{-1}}{2\ \mu\text{m}} = 1 \times 10^{-4}\ \text{ms}^{-1},$$

which is indeed about 2 orders of magnitude larger than the 'usual' permeabilities of a membrane for IAA (anion or protonated form).

Thus, using these conditions, (71) simplifies to

$$J = \left[\frac{\beta_1\beta_3}{\beta_2+\beta_3}\right] U_l^{n-1} \;-\; \left[\frac{\beta_2\beta_4}{\beta_2+\beta_3}\right] U_0^n. \tag{73}$$

The expression for J in the MK-model is – by definition:

$$J = pU_l^{n-1} + q\big(U_l^{n-1} - U_0^n\big) \;=\; (p+q)U_l^{n-1} - qU_0^n. \tag{74}$$

Comparing (73) to (74) immediately yields the expressions for p and q stated in (62).

One may now combine these preliminary expressions for p and q with (55) – (58) and obtain an expression for p and q in terms of physical and physiological quantities. At first sight it seems that equations become cumbersome. However, we make the physiologically reasonable assumption that apical and basal side are similar in terms of transport of auxin in protonated form and *apparent transport* of the anion form through essentially electrically neutral symporters:

$$\bar{P}^a_{\mathrm{AH}} = \bar{P}^b_{\mathrm{AH}} = \bar{P}^{rad}_{AH} \;=:\; \bar{P}_{\mathrm{AH}}. \tag{75}$$

This results in simpler expressions. In fact, under this assumption,

$$\begin{aligned}
\beta_2+\beta_3 &= 2\bar{P}_{\mathrm{AH}}(1-f_a) + (P^a_{\mathrm{A}^-}+P^b_{\mathrm{A}^-}) f_a \frac{EF}{RT}\,\frac{e^{-EF/RT}}{1-e^{-EF/RT}} \\
&= 2(1-f_a)\left[\bar{P}_{\mathrm{AH}} + \frac{K_a}{2h_a}(P^a_{\mathrm{A}^-}+P^b_{\mathrm{A}^-})\frac{EF}{RT}\,\frac{e^{-EF/RT}}{1-e^{-EF/RT}}\right] \\
&= 2\frac{h_a}{h_a+K_a}\bar{P}_{\mathrm{AH}}\left[1 + \frac{P^a_{\mathrm{A}^-}+P^b_{\mathrm{A}^-}}{2\bar{P}_{\mathrm{AH}}}\,\frac{K_a}{h_a}\,\frac{EF}{RT}\,\frac{e^{-EF/RT}}{1-e^{-EF/RT}}\right].
\end{aligned} \tag{76}$$

Under assumption (75) we also obtain

$$
\begin{aligned}
\beta_1\beta_3 - \beta_2\beta_4 \;=\;& \bar{P}_{\mathrm{AH}}(P^b_{\mathrm{A}^-} - P^a_{\mathrm{A}^-})(1-f_a)f_c\frac{EF}{RT}\frac{1}{1-e^{-EF/RT}} \\
&+ \bar{P}_{\mathrm{AH}}(P^a_{\mathrm{A}^-} - P^b_{\mathrm{A}^-})(1-f_c)f_a\frac{EF}{RT}\frac{e^{-EF/RT}}{1-e^{-EF/RT}} \qquad (77)\\
=\;& \bar{P}_{\mathrm{AH}}\frac{EF}{RT}\frac{e^{-EF/RT}}{1-e^{-EF/RT}}(P^b_{\mathrm{A}^-} - P^a_{\mathrm{A}^-}) \\
&\times\left[(1-f_a)f_c e^{EF/RT} - (1-f_c)f_a\right] \qquad (78)\\
=\;& \bar{P}_{\mathrm{AH}}\frac{EF}{RT}\frac{e^{-EF/RT}}{1-e^{-EF/RT}}(P^b_{\mathrm{A}^-} - P^a_{\mathrm{A}^-}) \\
&\times\frac{K_a}{(h_a+K_a)(h_c+K_a)}\left[h_a e^{EF/RT} - h_c\right]. \qquad (79)
\end{aligned}
$$

Finally, we get

$$
p = \left(P^b_{\mathrm{A}^-} - P^a_{\mathrm{A}^-}\right)\cdot\frac{1}{2}\frac{K_a}{h_c+K_a}\left[1+\frac{K_a}{h_a}\frac{P^b_{\mathrm{A}^-}+P^a_{\mathrm{A}^-}}{2\bar{P}_{\mathrm{AH}}}\frac{\frac{EF}{RT}e^{-EF/RT}}{1-e^{-EF/RT}}\right]^{-1}\times
\left[e^{EF/RT}-\frac{h_c}{h_a}\right]\cdot\frac{\frac{EF}{RT}e^{-EF/RT}}{1-e^{-EF/RT}}. \qquad (80)
$$

To simplify further, let

$$
\varepsilon := \frac{\frac{EF}{RT}e^{-EF/RT}}{1-e^{-EF/RT}} \qquad \text{and} \qquad M := \frac{h_a}{K_a}\frac{2\bar{P}_{\mathrm{AH}}}{P^b_{\mathrm{A}^-}+P^a_{\mathrm{A}^-}}.
$$

Then,

$$
\begin{aligned}
p &= \left(P^b_{\mathrm{A}^-} - P^a_{\mathrm{A}^-}\right)\left[e^{EF/RT}-\frac{h_c}{h_a}\right]\cdot\frac{1}{2}\frac{K_a}{h_c+K_a}\cdot\frac{\varepsilon}{1+\varepsilon M^{-1}} \\
&= \left(P^b_{\mathrm{A}^-} - P^a_{\mathrm{A}^-}\right)\left[e^{EF/RT}-\frac{h_c}{h_a}\right]\cdot\frac{h_a}{h_c+K_a}\cdot\frac{\bar{P}_{\mathrm{AH}}}{P^b_{\mathrm{A}^-}+P^a_{\mathrm{A}^-}}\frac{\varepsilon}{M+\varepsilon} \\
&= p_{\max}\cdot\frac{P^b_{\mathrm{A}^-}-P^a_{\mathrm{A}^-}}{P^b_{\mathrm{A}^-}+P^a_{\mathrm{A}^-}}\cdot\frac{\varepsilon}{M+\varepsilon},
\end{aligned}
$$

where the maximal value for p,

$$
p_{\max} = \bar{P}_{\mathrm{AH}}\left[e^{EF/RT}-\frac{h_c}{h_a}\right]\frac{h_a}{h_c+K_a}, \qquad (81)
$$

is controlled by membrane permeability for IAA in protonated form (P_{AH}), and combined electrophysiological-acidic properties: the proton motive force (second factor) and the acidity difference between apoplast and cytoplasm.

The derivation of an expression for $q = \beta_2\beta_4/(\beta_2 + \beta_3)$ is similar. Computations – again using the assumption that $\bar{P}^a_{\mathrm{AH}} = \bar{P}^b_{\mathrm{AH}} = \bar{P}_{\mathrm{AH}}$ – yield

$$\beta_2\beta_4 = \frac{K_a^2}{(h_a + K_a)(h_c + K_a)}\bar{P}^2_{\mathrm{AH}} \times \left[\frac{h_a}{K_a} + \frac{P^b_{\mathrm{A}^-}}{\bar{P}_{\mathrm{AH}}}\frac{EF}{RT}\frac{E^{-EF/RT}}{1 - e^{-EF/RT}}\right] \times \left[\frac{h_c}{K_a} + \frac{P^a_{\mathrm{A}^-}}{\bar{P}_{\mathrm{AH}}}e^{EF/RT}\,\frac{EF}{RT}\frac{E^{-EF/RT}}{1 - e^{-EF/RT}}\right]. \tag{82}$$

The expression for $\beta_2 + \beta_3$ is provided in (76). Putting the two together, we obtain

$$q = \bar{P}_{\mathrm{AH}} \cdot \frac{1}{2}\frac{K_a}{h_c + K_a}\left[1 + \frac{K_a}{h_a}\,\frac{P^b_{\mathrm{A}^-} + P^a_{\mathrm{A}^-}}{2\bar{P}_{\mathrm{AH}}}\,\frac{\frac{EF}{RT}\,e^{-EF/RT}}{1 - e^{-EF/RT}}\right]^{-1} \times \frac{h_c}{K_a}\left[1 + \frac{K_a}{h_a}\frac{P^b_{\mathrm{A}^-}}{\bar{P}_{\mathrm{AH}}}\,\frac{\frac{EF}{RT}\,e^{-EF/RT}}{1 - e^{-EF/RT}}\right] \cdot \left[1 + \frac{K_a}{h_c} \cdot \frac{P^a_{\mathrm{A}^-}}{\bar{P}_{\mathrm{AH}}}e^{EF/RT}\,\frac{\frac{EF}{RT}\,e^{-EF/RT}}{1 - e^{-EF/RT}}\right]. \tag{83}$$

We shall not try to further simplify this expression for q.

4.2.2 Expressions for a and b

Expressions for the rate parameters a and b for transversal transport of IAA between the U- and W-compartments have been derived in the Main Text:

$$a = \frac{S^0}{S_u}\beta_4, \qquad b = \frac{S^0}{S_w}\beta_3. \tag{84}$$

Here - recall - S^0 represents the total circumference of the curve that separates the U- and W-compartment in a cross section of the stem segment, S_u is the total area of the U-compartment in all vascular bundles together, and S_w is the area of its complement, that of the W-compartment. If S_{vb} is the total cross sectional area of all vascular bundles, then $S_w = S_{vb} - S_u$. Recall the expressions provided for a and b in Section 1.1, equation (10). Expressions (84) are the consequence of equating permeabilities k^+ to β_4 and k^- to β_3.

The anatomical parameter S_{vb} has been measured from a microscope image of a cross section of the inflorescence stem segment. The exact location of the PAT cell files within the vascular bundles is harder to determine. Which cells constitute these files is not yet fully clear, although in Boot *et al.* [1] putative cells have been indicated. Based on these indications the ratio $\alpha := S_u/S_{vb}$ has been estimated in various sample stem segments, resulting in a value $\alpha = 32\%$ that we used in all simulations. Note that

$$S_w = \frac{1-\alpha}{\alpha} \cdot S_u.$$

For the same reason, the circumference S^0 cannot be estimated well either. In the simulations we work with the *minimal* value for S^0, which corresponds to the circumference of a circle with area S_u. Thus, the geometrical part of a and b can be written as:

$$\frac{S^0}{S_u} = \frac{2}{\sqrt{S_u/\pi}} \qquad \text{and} \qquad \frac{S^0}{S_w} = \frac{\alpha}{1-\alpha}\frac{2}{\sqrt{S_u/\pi}}. \tag{85}$$

This can easily be verified, since $S^0 = 2\pi R_u$ and $S_u = \pi R_u^2$, where R_u is the radius of a circle with area S_u: $R_u = \sqrt{S_u/\pi}$. Consequently, the exchange rates a and b were related to β_3 and β_4 through

$$a = \frac{2}{\sqrt{S_u/\pi}} \cdot \beta_4 \qquad \text{and} \qquad b = \frac{2}{\sqrt{S_u/\pi}}\frac{\alpha}{1-\alpha} \cdot \beta_3 \tag{86}$$

Note that the ratio

$$\frac{b}{a} = \frac{\beta_3}{\beta_4}\frac{\alpha}{1-\alpha} \tag{87}$$

is independent of the value S^0 that is hard to determine. It is reminiscent to an accumulation ratio.

4.2.3 Auxiliary parameters values in the LPTF

In the quadrupple AUX1/LAX1-3 mutant the (apparent) permeability for the protonated form is supposed to have been reduced to its minimal value, which is that of 'unmediated' transport of the protonated form P_{AH}. This value is – according to fitting of the quadrupple – of the order of magnitude of 1.5×10^{-7} m/s. The LPTF works with the apparent permeability for the protonated form:

$$\bar{P}_{AH} = P_{AH} + k^{co}K_a,$$

which is varied in the fitting procedure. It varies in the range of roughly 2×10^{-7} ms^{-1} to 5×10^{-6} ms^{-1}.

We take one order of magnitude smaller than P_{AH} as permeability $P^a_{A^-}$ for the transport of the anion through the lipid bilayer of the plasma membrane – in absence of electric field, i.e.

$$P^a_{A^-} = 1.5 \times 10^{-8}\ \mathrm{ms}^{-1}.$$

This reflects the observation that the charged IAA anion is expected to be attracted still to the fatty-acid molecules in the plasma membrane.

5 Determining LPTF key parameters from macroscopic fit of data

The microscopic key parameters $\bar{P}_{AH}$ and P^b_{A-} in the LPTF model map to the macroscopic parameters V, a, b and D_u in the BHL model. This is effected by means of the expressions for the $\beta_1, \ldots \beta_4$ in (55)–(58), the expressions for p and q, and a and b, in terms of these β_i in Equations (62) and (84), and finally the expression of V and D_u in p and q in Equations (48) and (49).

In Figure 7 we illustrated schematically how this map transforms a rectangular part of the $(\bar{P}_{AH}, P^b_{A-})$-parameter space into a the three-dimensional (V, a, b)-part of BHL parameter space.

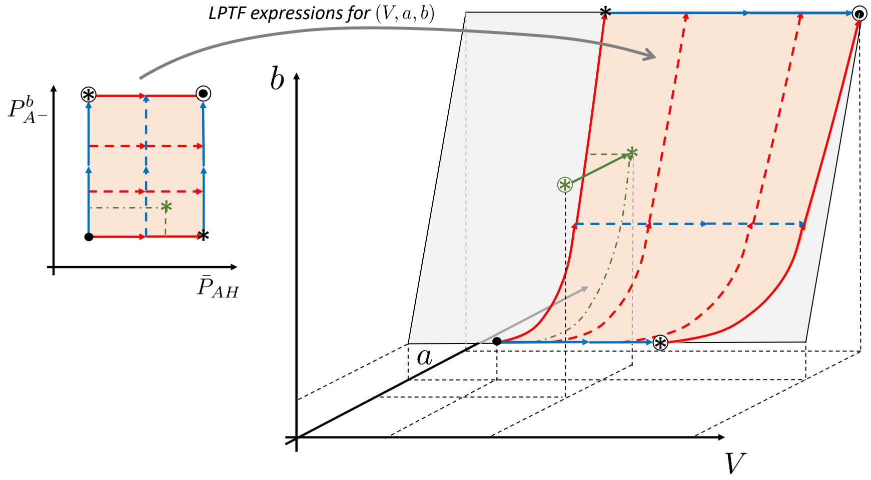

Figure 7: *Schematic presentation of the map that sends the key parameters $\bar{P}_{AH}$ and P^b_{A-} of the LPTF model into the three-dimensional (V, a, b)-part of BHL parameter space. Corresponding corner points under the mapping are indicated by similar symbols. The green encircled asterix is a parameter value in BHL parameter space. It can be projected onto the image of LPTF paramater space (light orange), e.g. by keeping V and b constant and only varying a. This results in the projection and corresponding LPTF parameter pair indicated by a green asterix.*

Of the LPTF key parameters, β_3 and β_4 depend on $\bar{P}_{AH}$ only, in an affine linear fashion, see (57) and (58). Therefore, the image of a part of $(\bar{P}_{AH}, P^b_{A-})$-parameter space lies in plane, parallel to the V-axis (indicated in light grey

in Figure 7). This plane has a large slope in the (a,b)-coordinate plane:

$$\frac{\Delta b}{\Delta a} = \frac{S_u \Delta \beta_3}{S_w \Delta \beta_4} = \frac{\alpha}{1-\alpha}\frac{1-f_a}{1-f_c} \approx 20. \tag{88}$$

$\bar{P}_{AH}$ is expected to vary in the range $1 \times 10^{-7} - 5 \times 10^{-6}$ ms^{-1}. With the selected (fixed) auxiliary microscopic parameters in the LPTF model (see Table 1), this amounts to a range of variantion for a of about $3.4 \times 10^{-4} - 5.8 \times 10^{-4}$ s^{-1}, while b varies in the range $8.7 \times 10^{-5} - 4.4 \times 10^{-3}$ s^{-1}. Note that a varies by roughly a factor 2, while b varies by almost two orders of magnitude. Since a and b (through β_4 and β_3) do not depend on $P^b_{A^-}$, only V will change with increasing $P^b_{A^-}$. It is then increasing as well, for fixed (a,b)-value (see blue arrows in Figure 7).

A parameter triple (V,a,b) in the BHL-model (green encircled astrix in Figure 7) can be projected onto the plane (light grey) in which the image under the parameter map (light orange) lies. This can be done by orthogonal projection. However, since the plane has a steep slope in the (a,b)-coordinate plane, this orthogonal projection does not deviate much from a simple projection in which the (V,b)-coordinates are kept fixed, for points close to the plane. Since a good fit to the BHL model is expected to be close to a fit with the LPTF model, we employed the latter, simpler, projection to determine from a fit to the BHL model appropriate key parameter values in the LPTF model (indicated by a green asterix).

6 Expression levels

In this section we present the supporting material for Figure 7 and Figure 8 of the Main Text, presenting the expression levels of the AUX1/LAX1-3 genes and PIN1, PIN3, PIN4, PIN6 and PIN7 genes in wild type and various mutant plants.

6.1 Overview of primers

Table 5 gives an overview of the primers used.

**Primers**		**Amplification factor**
PIN1 FW :	TCCTCCTCCATGTTGCCATTATC	2.03
PIN1 RV :	TGTAGTAGAGAAGAGTTATGGGCA	
PIN3 FW :	AAGGCGGAAGATCTGACCAAGG	1.77
PIN3 RV :	TGCTGGATGAGCTACAGCTTTG	
PIN4 FW :	ACAACGTGGCAACGGAACAATC	1.97
PIN4 RV :	GCCGATATCATCACCACCACTC	
PIN6 FW:	TGGGCCGTTTTCTTCAAAGC	2.10
PIN6 RV :	GATTGATCCGGCTGCTTGAC	
PIN7 FW:	CGTGTGGCCATTGTTCAAGCTG	1.96
PIN7 RV :	CCCTGTACTCAAGATTGCGGGATG	
AUX1 FW:	ATGACAACGGAACAGATCAG	2.03
AUX1 RV:	GTGCCATAGGAAATTGCTTAG	
LAX1 FW:	TACTCCGAGACCTTCCAACTACG	2.13
LAX1 RV:	TCCACCGCCACCACTTCC	
LAX2 FW:	GGAGAACGGTGAGAAAGC	2.12
LAX2 RV:	TCAGATAGCTTAGATTTGATGTC	
LAX3 FW:	TCACCATTGCTTCACTCCTTC	2.04
LAX3 RV:	AAGCACCATTGTGGTTGGAC	
qβ-Tubulin-6 FW:	TGGGAACTCTGCTCATATCT	2.05
qβ-Tubulin-6 RV:	GAAAGGAATGAGGTTCACTG	

Table 5: *List of primers used for the RT-qPCR analysis of the PINs 1,3,4,6 and 7 and the AUX1, LAX1,2,3 genes and the control gene β-Tubulin-6.*

6.2 Statistical tests

We checked the significance of differences in mean expression level of the AUX1, LAX1-LAX3 genes and those of PIN1, PIN3, PIN4, PIN6 and PIN7

in wild type plants and the mutant plants: *pin1*, pML::PIN1:GFP/*pin1* line A and pML::PIN1:GFP/*pin1* line B. We used a standard confidence level of 5%. The unpaired two-sided Student's t-test was used to test the null hypothesis that the mean gene expression of the indicated gene in the mutant plant experiments is equal to that in wild type plants. Table 6 and Table 7 presents the p-values for the result of the t-test for the AUX1/LAX1-LAX3 genes and PIN genes, respectively.

Mutant plant:	p-value for gene: AUX1	LAX1	LAX2	LAX3
pin1	0.0053*	0.0282*	<0.001*	0.1127
pML::PIN1:GFP/*pin1* line A	0.0109*	0.9271	0.0077*	0.0375*
pML::PIN1:GFP/*pin1* line B	<0.001*	0.0032*	0.2475	0.6387

Table 6: *p-values of mean expression levels of AUX1 and LAX1-LAX3 in mutant plants as indicated, compared to the mean expression levels in wild type plants, according to the unpaired two-sided Student's t-test.*

Mutant plant:	p-value for gene: PIN1	PIN3	PIN4	PIN6	PIN7
pin1	0.0007*	0.0775	0.0330*	0.0024*	0.3779
pML::PIN1:GFP/*pin1* line A	0.0214*	0.6711	0.0858	0.8030	0.5086
pML::PIN1:GFP/*pin1* line B	0.0080*	0.0248*	0.0151*	0.0380*	0.3588

Table 7: *p-values of mean expression levels of PIN1, PIN3, PIN4, PIN6 and PIN7 in mutant plants as indicated, compared to the mean expression levels in wild type plants, according to the unpaired two-sided Student's t-test.*

.

References

[1] Boot, K.J.M., Hille, S.C., Libbenga, K.R., Peletier L.A., van Spronsen, P.C., van Duijn, B., Offringa, R. (2016), Modelling the dynamics of polar auxin transport in inflorescence stems of *Arabidopsis thaliana*. *J. Exp. Botany* **67**, pp. 649-666. doi.org/10.1093/jxb/erv471.

[2] Fall, Ch.P., Marland, E.S., Wagner, J.M., Tyson, J.J. (eds.) (2005). *Computational Cell Biology*, Interdisciplinary Applied Mathematics, Vol. 20, Springer.

[3] Gastwirth, J.L., Gel, Y.R., Miao, W. (2009), The impact of Levene's Test of equality of variances on statistical theory and practice. *Statistical Science* **24**(3), pp.343–360.

[4] Hines, W.G.S., O'Hara Hines, R.J. (2000), Increased power with modified forms of the Levene (Med) Test for heterogeneity of variance. *Biometrics* **56**, pp. 451–454.

[5] Keener, J., Sneyd, J. (2009), *Mathematical Physiology; I: Cellular Physiology*, 2nd Ed., Springer Science+Business Media LLC: New York.

[6] King, E.L., Altman, C.(1956). A schematic method for deriving the rate laws for enzyme-catalyzed reactions. *J. Phys. Chem.* **60**, pp. 1375–1378.

[7] Kramer, E.M. (2002). A mathematical model of pattern formation in the vascular cambium of trees, *J. Theor. Biol.* **216**, 147–158.

[8] Mitchison, G.J. (1980). The dynamics of auxin transport, *Proc. R. Soc. Lond. B* **209**, 489–511.

[9] Raven, J.A. (1967). Ion transport in *Hydrodictyon africanum*, *J. gen. Physiol.* **50**(6), 1607–1625.

[10] Rice, J.A. (1995), *Mathematical Statistics and Data Analysis*, 2nd Ed., Duxbury Press, Wadsworth Inc.: Belmont.

[11] Sanders, D., Hansen, U.-P., Gradmann, D., Slayman, C.L. (1984). Generalized kinetic analysis of ion-driven cotransport systems: a unified interpretation of selective ionic effects on Michaelis parameters, *J. Membrane Biol.* **77**, pp. 123–152.
